# Supplementary material for: Optimizing breeding strategies for early-maturing white maize through genetic diversity and population structure
Source: PLoS One. 2025 Feb 24;20(2):e0316793. doi: 10.1371/journal.pone.0316793 (PMC11849899; doi:10.1371/journal.pone.0316793)
Supplement: S1 File — (DOCX) [file pone.0316793.s001.docx]

Table S1: Population stratification based on Bayesian statistics approach implemented in STRUCTURE revealed two genetic groups with 9.5% level of admixture

| Geno | Class | Q1 | Q2 | Cluster |
| --- | --- | --- | --- | --- |
| TZEI 2231 | TZEI 65 x ENT 11 | 0.95 | 0.05 | 1 |
| TZEI 2233 | TZEI 65 x ENT 11 | 1.00 | 0.00 | 1 |
| TZEI 2234 | TZEI 65 x ENT 11 | 1.00 | 0.00 | 1 |
| TZEI 2235 | TZEI 65 x ENT 11 | 0.99 | 0.01 | 1 |
| TZEI 2239 | TZEI 65 x ENT 11 | 1.00 | 0.00 | 1 |
| TZEI 2240 | TZEI 65 x ENT 11 | 1.00 | 0.00 | 1 |
| TZEI 2241 | TZEI 65 x ENT 11 | 0.92 | 0.08 | 1 |
| TZEI 2242 | TZEI 65 x ENT 11 | 1.00 | 0.00 | 1 |
| TZEI 2243 | TZEI 65 x ENT 11 | 0.99 | 0.01 | 1 |
| TZEI 2245 | TZEI 65 x ENT 11 | 0.99 | 0.01 | 1 |
| TZEI 2246 | TZEI 65 x ENT 11 | 1.00 | 0.00 | 1 |
| TZEI 2247 | TZEI 65 x ENT 11 | 1.00 | 0.00 | 1 |
| TZEI 2252 | TZEI 65 x ENT 11 | 1.00 | 0.00 | 1 |
| TZEI 2253 | TZEI 65 x ENT 11 | 1.00 | 0.00 | 1 |
| TZEI 2254 | TZEI 65 x ENT 11 | 1.00 | 0.00 | 1 |
| TZEI 2255 | TZEI 65 x ENT 11 | 1.00 | 0.00 | 1 |
| TZEI 2256 | TZEI 65 x ENT 11 | 0.96 | 0.05 | 1 |
| TZEI 2258 | TZEI 65 x ENT 11 | 1.00 | 0.00 | 1 |
| TZEI 2259 | TZEI 65 x ENT 11 | 1.00 | 0.00 | 1 |
| TZEI 2264 | TZEI 65 x ENT 11 | 1.00 | 0.00 | 1 |
| TZEI 2265 | TZEI 65 x ENT 11 | 1.00 | 0.00 | 1 |
| TZEI 2266 | TZEI 65 x ENT 11 | 0.94 | 0.06 | 1 |
| TZEI 2267 | TZEI 65 x ENT 11 | 1.00 | 0.00 | 1 |
| TZEI 2268 | TZEI 65 x ENT 11 | 1.00 | 0.00 | 1 |
| TZEI 2270 | TZEI 65 x ENT 11 | 1.00 | 0.00 | 1 |
| TZEI 2271 | TZEI 65 x ENT 11 | 1.00 | 0.00 | 1 |
| TZEI 2275 | TZEI 65 x ENT 11 | 1.00 | 0.00 | 1 |
| TZEI 2276 | TZEI 65 x ENT 11 | 1.00 | 0.00 | 1 |
| TZEI 2277 | TZEI 65 x ENT 11 | 1.00 | 0.00 | 1 |
| TZEI 2278 | TZEI 65 x ENT 11 | 1.00 | 0.00 | 1 |
| TZEI 2279 | TZEI 65 x ENT 11 | 0.96 | 0.04 | 1 |
| TZEI 2281 | TZEI 65 x ENT 11 | 1.00 | 0.00 | 1 |
| TZEI 2286 | TZEI 65 x ENT 11 | 1.00 | 0.00 | 1 |
| TZEI 2287 | TZEI 65 x ENT 11 | 1.00 | 0.00 | 1 |
| TZEI 2288 | TZEI 65 x ENT 11 | 1.00 | 0.00 | 1 |
| TZEI 2289 | TZEI 65 x ENT 11 | 1.00 | 0.00 | 1 |
| TZEI 2290 | TZEI 65 x ENT 11 | 1.00 | 0.00 | 1 |
| TZEI 2536 | DTE STR-W Syn Pop C4 | 1.00 | 0.00 | 1 |
| TZEI 2537 | DTE STR-W Syn Pop C4 | 1.00 | 0.00 | 1 |
| TZEI 2538 | DTE STR-W Syn Pop C4 | 1.00 | 0.00 | 1 |
| TZEI 2543 | DTE STR-W Syn Pop C4 | 1.00 | 0.00 | 1 |
| TZEI 2544 | DTE STR-W Syn Pop C4 | 1.00 | 0.00 | 1 |
| TZEI 2545 | DTE STR-W Syn Pop C4 | 1.00 | 0.00 | 1 |
| TZEI 2546 | DTE STR-W Syn Pop C4 | 1.00 | 0.00 | 1 |
| TZEI 2548 | DTE STR-W Syn Pop C4 | 1.00 | 0.00 | 1 |
| TZEI 2549 | DTE STR-W Syn Pop C4 | 1.00 | 0.00 | 1 |
| TZEI 2550 | DTE STR-W Syn Pop C4 | 0.99 | 0.01 | 1 |
| TZEI 2555 | DTE STR-W Syn Pop C4 | 1.00 | 0.00 | 1 |
| TZEI 2556 | DTE STR-W Syn Pop C4 | 1.00 | 0.00 | 1 |
| TZEI 2557A | DTE STR-W Syn Pop C4 | 1.00 | 0.00 | 1 |
| TZEI 2557B | DTE STR-W Syn Pop C4 | 1.00 | 0.00 | 1 |
| TZEI 2559 | DTE STR-W Syn Pop C4 | 1.00 | 0.01 | 1 |
| TZEI 2560 | DTE STR-W Syn Pop C4 | 0.99 | 0.01 | 1 |
| TZEI 2561 | DTE STR-W Syn Pop C4 | 1.00 | 0.00 | 1 |
| TZEI 2566 | DTE STR-W Syn Pop C4 | 1.00 | 0.00 | 1 |
| TZEI 2567 | DTE STR-W Syn Pop C4 | 0.99 | 0.01 | 1 |
| TZEI 2568 | DTE STR-W Syn Pop C4 | 1.00 | 0.00 | 1 |
| TZEI 2238 | TZEI 65 x ENT 11 | 0.18 | 0.82 | 2 |
| TZEI 2263 | TZEI 65 x ENT 11 | 0.06 | 0.94 | 2 |
| TZEI 2273 | TZEI 65 x ENT 11 | 0.16 | 0.84 | 2 |
| TZEI 2274 | TZEI 65 x ENT 11 | 0.07 | 0.93 | 2 |
| TZEI 2285 | TZEI 65 x ENT 11 | 0.19 | 0.81 | 2 |
| TZEI 2429 | DTE STR-W Syn Pop C4 | 0.16 | 0.84 | 2 |
| TZEI 2430 | DTE STR-W Syn Pop C4 | 0.00 | 1.00 | 2 |
| TZEI 2431 | DTE STR-W Syn Pop C4 | 0.07 | 0.93 | 2 |
| TZEI 2432 | DTE STR-W Syn Pop C4 | 0.00 | 1.00 | 2 |
| TZEI 2433 | DTE STR-W Syn Pop C4 | 0.00 | 1.00 | 2 |
| TZEI 2434 | DTE STR-W Syn Pop C4 | 0.02 | 0.98 | 2 |
| TZEI 2437 | DTE STR-W Syn Pop C4 | 0.05 | 0.96 | 2 |
| TZEI 2438 | DTE STR-W Syn Pop C4 | 0.04 | 0.96 | 2 |
| TZEI 2439 | DTE STR-W Syn Pop C4 | 0.00 | 1.00 | 2 |
| TZEI 2440 | DTE STR-W Syn Pop C4 | 0.01 | 0.99 | 2 |
| TZEI 2441 | DTE STR-W Syn Pop C4 | 0.13 | 0.87 | 2 |
| TZEI 2442 | DTE STR-W Syn Pop C4 | 0.11 | 0.89 | 2 |
| TZEI 2443 | DTE STR-W Syn Pop C4 | 0.20 | 0.80 | 2 |
| TZEI 2444 | DTE STR-W Syn Pop C4 | 0.04 | 0.96 | 2 |
| TZEI 2445 | DTE STR-W Syn Pop C4 | 0.14 | 0.87 | 2 |
| TZEI 2446 | DTE STR-W Syn Pop C4 | 0.16 | 0.84 | 2 |
| TZEI 2447 | DTE STR-W Syn Pop C4 | 0.04 | 0.96 | 2 |
| TZEI 2448 | DTE STR-W Syn Pop C4 | 0.14 | 0.87 | 2 |
| TZEI 2449 | DTE STR-W Syn Pop C4 | 0.14 | 0.86 | 2 |
| TZEI 2450 | DTE STR-W Syn Pop C4 | 0.12 | 0.88 | 2 |
| TZEI 2451 | DTE STR-W Syn Pop C4 | 0.11 | 0.89 | 2 |
| TZEI 2452 | DTE STR-W Syn Pop C4 | 0.01 | 0.99 | 2 |
| TZEI 2453 | DTE STR-W Syn Pop C4 | 0.19 | 0.82 | 2 |
| TZEI 2455 | DTE STR-W Syn Pop C4 | 0.18 | 0.82 | 2 |
| TZEI 2456 | DTE STR-W Syn Pop C4 | 0.02 | 0.98 | 2 |
| TZEI 2457 | DTE STR-W Syn Pop C4 | 0.20 | 0.80 | 2 |
| TZEI 2459 | DTE STR-W Syn Pop C4 | 0.09 | 0.92 | 2 |
| TZEI 2460 | DTE STR-W Syn Pop C4 | 0.13 | 0.87 | 2 |
| TZEI 2461 | DTE STR-W Syn Pop C4 | 0.12 | 0.88 | 2 |
| TZEI 2462 | DTE STR-W Syn Pop C4 | 0.11 | 0.89 | 2 |
| TZEI 2463 | DTE STR-W Syn Pop C4 | 0.13 | 0.88 | 2 |
| TZEI 2464 | DTE STR-W Syn Pop C4 | 0.01 | 0.99 | 2 |
| TZEI 2465 | DTE STR-W Syn Pop C4 | 0.04 | 0.96 | 2 |
| TZEI 2466 | DTE STR-W Syn Pop C4 | 0.05 | 0.95 | 2 |
| TZEI 2467 | DTE STR-W Syn Pop C4 | 0.18 | 0.82 | 2 |
| TZEI 2468 | DTE STR-W Syn Pop C4 | 0.01 | 0.99 | 2 |
| TZEI 2469 | DTE STR-W Syn Pop C4 | 0.01 | 0.99 | 2 |
| TZEI 2470 | DTE STR-W Syn Pop C4 | 0.10 | 0.90 | 2 |
| TZEI 2471 | DTE STR-W Syn Pop C4 | 0.10 | 0.90 | 2 |
| TZEI 2472 | DTE STR-W Syn Pop C4 | 0.10 | 0.90 | 2 |
| TZEI 2473 | DTE STR-W Syn Pop C4 | 0.13 | 0.87 | 2 |
| TZEI 2474 | DTE STR-W Syn Pop C4 | 0.10 | 0.90 | 2 |
| TZEI 2475 | DTE STR-W Syn Pop C4 | 0.06 | 0.94 | 2 |
| TZEI 2476 | DTE STR-W Syn Pop C4 | 0.01 | 0.99 | 2 |
| TZEI 2477 | DTE STR-W Syn Pop C4 | 0.04 | 0.96 | 2 |
| TZEI 2478 | DTE STR-W Syn Pop C4 | 0.05 | 0.95 | 2 |
| TZEI 2479 | DTE STR-W Syn Pop C4 | 0.20 | 0.80 | 2 |
| TZEI 2480 | DTE STR-W Syn Pop C4 | 0.01 | 0.99 | 2 |
| TZEI 2481 | DTE STR-W Syn Pop C4 | 0.13 | 0.87 | 2 |
| TZEI 2482 | DTE STR-W Syn Pop C4 | 0.04 | 0.96 | 2 |
| TZEI 2483 | DTE STR-W Syn Pop C4 | 0.12 | 0.88 | 2 |
| TZEI 2484 | DTE STR-W Syn Pop C4 | 0.02 | 0.98 | 2 |
| TZEI 2485 | DTE STR-W Syn Pop C4 | 0.15 | 0.85 | 2 |
| TZEI 2486 | DTE STR-W Syn Pop C4 | 0.12 | 0.88 | 2 |
| TZEI 2489 | DTE STR-W Syn Pop C4 | 0.01 | 0.99 | 2 |
| TZEI 2490 | DTE STR-W Syn Pop C4 | 0.04 | 0.96 | 2 |
| TZEI 2491 | DTE STR-W Syn Pop C4 | 0.05 | 0.95 | 2 |
| TZEI 2493 | DTE STR-W Syn Pop C4 | 0.01 | 0.99 | 2 |
| TZEI 2494 | DTE STR-W Syn Pop C4 | 0.05 | 0.95 | 2 |
| TZEI 2495 | DTE STR-W Syn Pop C4 | 0.12 | 0.88 | 2 |
| TZEI 2496 | DTE STR-W Syn Pop C4 | 0.12 | 0.88 | 2 |
| TZEI 2497 | DTE STR-W Syn Pop C4 | 0.07 | 0.93 | 2 |
| TZEI 2498 | DTE STR-W Syn Pop C4 | 0.16 | 0.84 | 2 |
| TZEI 2499 | DTE STR-W Syn Pop C4 | 0.14 | 0.87 | 2 |
| TZEI 2500 | DTE STR-W Syn Pop C4 | 0.08 | 0.92 | 2 |
| TZEI 2501 | DTE STR-W Syn Pop C4 | 0.01 | 0.99 | 2 |
| TZEI 2502 | DTE STR-W Syn Pop C4 | 0.17 | 0.83 | 2 |
| TZEI 2503 | DTE STR-W Syn Pop C4 | 0.07 | 0.94 | 2 |
| TZEI 2505 | DTE STR-W Syn Pop C4 | 0.08 | 0.92 | 2 |
| TZEI 2506 | DTE STR-W Syn Pop C4 | 0.07 | 0.93 | 2 |
| TZEI 2507 | DTE STR-W Syn Pop C4 | 0.08 | 0.92 | 2 |
| TZEI 2508 | DTE STR-W Syn Pop C4 | 0.13 | 0.87 | 2 |
| TZEI 2509 | DTE STR-W Syn Pop C4 | 0.01 | 0.99 | 2 |
| TZEI 2510 | DTE STR-W Syn Pop C4 | 0.12 | 0.88 | 2 |
| TZEI 2511 | DTE STR-W Syn Pop C4 | 0.06 | 0.94 | 2 |
| TZEI 2512 | DTE STR-W Syn Pop C4 | 0.10 | 0.90 | 2 |
| TZEI 2513 | DTE STR-W Syn Pop C4 | 0.01 | 0.99 | 2 |
| TZEI 2514 | DTE STR-W Syn Pop C4 | 0.11 | 0.89 | 2 |
| TZEI 2515 | DTE STR-W Syn Pop C4 | 0.20 | 0.80 | 2 |
| TZEI 2516 | DTE STR-W Syn Pop C4 | 0.06 | 0.94 | 2 |
| TZEI 2517 | DTE STR-W Syn Pop C4 | 0.13 | 0.87 | 2 |
| TZEI 2518 | DTE STR-W Syn Pop C4 | 0.04 | 0.96 | 2 |
| TZEI 2519 | DTE STR-W Syn Pop C4 | 0.13 | 0.87 | 2 |
| TZEI 2520 | DTE STR-W Syn Pop C4 | 0.01 | 0.99 | 2 |
| TZEI 2521 | DTE STR-W Syn Pop C4 | 0.13 | 0.87 | 2 |
| TZEI 2522 | DTE STR-W Syn Pop C4 | 0.05 | 0.95 | 2 |
| TZEI 2523 | DTE STR-W Syn Pop C4 | 0.06 | 0.94 | 2 |
| TZEI 2524 | DTE STR-W Syn Pop C4 | 0.01 | 0.99 | 2 |
| TZEI 2525 | DTE STR-W Syn Pop C4 | 0.12 | 0.88 | 2 |
| TZEI 2526 | DTE STR-W Syn Pop C4 | 0.19 | 0.81 | 2 |
| TZEI 2528 | DTE STR-W Syn Pop C4 | 0.18 | 0.82 | 2 |
| TZEI 2529 | DTE STR-W Syn Pop C4 | 0.07 | 0.94 | 2 |
| TZEI 2530 | DTE STR-W Syn Pop C4 | 0.15 | 0.85 | 2 |
| TZEI 2531 | DTE STR-W Syn Pop C4 | 0.02 | 0.99 | 2 |
| TZEI 2532 | DTE STR-W Syn Pop C4 | 0.15 | 0.85 | 2 |
| TZEI 2533 | DTE STR-W Syn Pop C4 | 0.05 | 0.95 | 2 |
| TZEI 2534 | DTE STR-W Syn Pop C4 | 0.04 | 0.96 | 2 |
| TZEI 2542 | DTE STR-W Syn Pop C4 | 0.16 | 0.84 | 2 |
| TZEI 2554 | DTE STR-W Syn Pop C4 | 0.09 | 0.91 | 2 |
| TZEI 2565 | DTE STR-W Syn Pop C4 | 0.11 | 0.89 | 2 |
| TZEI 2695 | TZE-W Pop DT C5 STR C5 | 0.01 | 0.99 | 2 |
| TZEI 2696 | TZE-W Pop DT C5 STR C5 | 0.06 | 0.94 | 2 |
| TZEI 2697 | TZE-W Pop DT C5 STR C5 | 0.02 | 0.98 | 2 |
| TZEI 2698 | TZE-W Pop DT C5 STR C5 | 0.05 | 0.95 | 2 |
| TZEI 2699 | TZE-W Pop DT C5 STR C5 | 0.02 | 0.98 | 2 |
| TZEI 2700 | TZE-W Pop DT C5 STR C5 | 0.05 | 0.95 | 2 |
| TZEI 2701 | TZE-W Pop DT C5 STR C5 | 0.01 | 0.99 | 2 |
| TZEI 2703 | TZE-W Pop DT C5 STR C5 | 0.05 | 0.95 | 2 |
| TZEI 2704 | TZE-W Pop DT C5 STR C5 | 0.01 | 0.99 | 2 |
| TZEI 2705 | TZE-W Pop DT C5 STR C5 | 0.06 | 0.94 | 2 |
| TZEI 2706 | TZE-W Pop DT C5 STR C5 | 0.01 | 1.00 | 2 |
| TZEI 2707 | TZE-W Pop DT C5 STR C5 | 0.01 | 0.99 | 2 |
| TZEI 2708 | TZE-W Pop DT C5 STR C5 | 0.05 | 0.95 | 2 |
| TZEI 2710 | TZE-W Pop DT C5 STR C5 | 0.02 | 0.98 | 2 |
| TZEI 2711 | TZE-W Pop DT C5 STR C5 | 0.06 | 0.94 | 2 |
| TZEI 2712 | TZE-W Pop DT C5 STR C5 | 0.12 | 0.88 | 2 |
| TZEI 2713 | TZE-W Pop DT C5 STR C5 | 0.07 | 0.93 | 2 |
| TZEI 2714 | TZE-W Pop DT C5 STR C5 | 0.02 | 0.98 | 2 |
| TZEI 2715 | TZE-W Pop DT C5 STR C5 | 0.10 | 0.91 | 2 |
| TZEI 2716 | TZE-W Pop DT C5 STR C5 | 0.00 | 1.00 | 2 |
| TZEI 2717 | TZE-W Pop DT C5 STR C5 | 0.01 | 0.99 | 2 |
| TZEI 2718 | TZE-W Pop DT C5 STR C5 | 0.12 | 0.88 | 2 |
| TZEI 2719 | TZE-W Pop DT C5 STR C5 | 0.05 | 0.95 | 2 |
| TZEI 2720 | TZE-W Pop DT C5 STR C5 | 0.01 | 0.99 | 2 |
| TZEI 2722 | TZE-W Pop DT C5 STR C5 | 0.01 | 0.99 | 2 |
| TZEI 2724 | TZE-W Pop DT C5 STR C5 | 0.01 | 0.99 | 2 |
| TZEI 2725A | TZE-W Pop DT C5 STR C5 | 0.00 | 1.00 | 2 |
| TZEI 2725B | TZE-W Pop DT C5 STR C5 | 0.01 | 0.99 | 2 |
| TZEI 2726A | TZE-W Pop DT C5 STR C5 | 0.07 | 0.93 | 2 |
| TZEI 2726B | TZE-W Pop DT C5 STR C5 | 0.00 | 1.00 | 2 |
| TZEI 2727 | TZE-W Pop DT C5 STR C5 | 0.01 | 0.99 | 2 |
| TZEI 2728 | TZE-W Pop DT C5 STR C5 | 0.00 | 1.00 | 2 |
| TZEI 2729 | TZE-W Pop DT C5 STR C5 | 0.00 | 1.00 | 2 |
| TZEI 2730 | TZE-W Pop DT C5 STR C5 | 0.08 | 0.92 | 2 |
| TZEI 2731 | TZE-W Pop DT C5 STR C5 | 0.06 | 0.94 | 2 |
| TZEI 2732 | TZE-W Pop DT C5 STR C5 | 0.01 | 0.99 | 2 |
| TZEI 2733 | TZE-W Pop DT C5 STR C5 | 0.07 | 0.93 | 2 |
| TZEI 2734 | TZE-W Pop DT C5 STR C5 | 0.10 | 0.90 | 2 |
| TZEI 2735A | TZE-W Pop DT C5 STR C5 | 0.01 | 1.00 | 2 |
| TZEI 2735B | TZE-W Pop DT C5 STR C5 | 0.01 | 0.99 | 2 |
| TZEI 2736A | TZE-W Pop DT C5 STR C5 | 0.00 | 1.00 | 2 |
| TZEI 2736B | TZE-W Pop DT C5 STR C5 | 0.00 | 1.00 | 2 |
| TZEI 2737 | TZE-W Pop DT C5 STR C5 | 0.14 | 0.86 | 2 |
| TZEI 2738 | TZE-W Pop DT C5 STR C5 | 0.00 | 1.00 | 2 |
| TZEI 2739 | TZE-W Pop DT C5 STR C5 | 0.03 | 0.97 | 2 |
| TZEI 2740 | TZE-W Pop DT C5 STR C5 | 0.02 | 0.98 | 2 |
| TZEI 2741A | TZE-W Pop DT C5 STR C5 | 0.02 | 0.98 | 2 |
| TZEI 2741B | TZE-W Pop DT C5 STR C5 | 0.03 | 0.97 | 2 |
| TZEI 2742 | TZE-W Pop DT C5 STR C5 | 0.06 | 0.94 | 2 |
| TZEI 2743 | TZE-W Pop DT C5 STR C5 | 0.11 | 0.90 | 2 |
| TZEI 2744 | TZE-W Pop DT C5 STR C5 | 0.04 | 0.96 | 2 |
| TZEI 2745 | TZE-W Pop DT C5 STR C5 | 0.01 | 0.99 | 2 |
| TZEI 2746A | TZE-W Pop DT C5 STR C5 | 0.00 | 1.00 | 2 |
| TZEI 2746B | TZE-W Pop DT C5 STR C5 | 0.01 | 0.99 | 2 |
| TZEI 2747 | TZE-W Pop DT C5 STR C5 | 0.12 | 0.88 | 2 |
| TZEI 2748 | TZE-W Pop DT C5 STR C5 | 0.00 | 1.00 | 2 |
| TZEI 2749 | TZE-W Pop DT C5 STR C5 | 0.04 | 0.96 | 2 |
| TZEI 2750 | TZE-W Pop DT C5 STR C5 | 0.08 | 0.92 | 2 |
| TZEI 2751 | TZE-W Pop DT C5 STR C5 | 0.02 | 0.98 | 2 |
| TZEI 2752 | TZE-W Pop DT C5 STR C5 | 0.01 | 0.99 | 2 |
| TZEI 2753 | TZE-W Pop DT C5 STR C5 | 0.01 | 0.99 | 2 |
| TZEI 2754 | TZE-W Pop DT C5 STR C5 | 0.00 | 1.00 | 2 |
| TZEI 2755 | TZE-W Pop DT C5 STR C5 | 0.01 | 0.99 | 2 |
| TZEI 2756 | TZE-W Pop DT C5 STR C5 | 0.01 | 0.99 | 2 |
| TZEI 2757 | TZE-W Pop DT C5 STR C5 | 0.01 | 0.99 | 2 |
| TZEI 2758 | TZE-W Pop DT C5 STR C5 | 0.01 | 0.99 | 2 |
| TZEI 2759 | TZE-W Pop DT C5 STR C5 | 0.07 | 0.93 | 2 |
| TZEI 2760 | TZE-W Pop DT C5 STR C5 | 0.00 | 1.00 | 2 |
| TZEI 2761 | TZE-W Pop DT C5 STR C5 | 0.01 | 1.00 | 2 |
| TZEI 2762 | TZE-W Pop DT C5 STR C5 | 0.02 | 0.98 | 2 |
| TZEI 2763 | TZE-W Pop DT C5 STR C5 | 0.09 | 0.91 | 2 |
| TZEI 2764 | TZE-W Pop DT C5 STR C5 | 0.00 | 1.00 | 2 |
| TZEI 2765 | TZE-W Pop DT C5 STR C5 | 0.01 | 0.99 | 2 |
| TZEI 2766 | TZE-W Pop DT C5 STR C5 | 0.00 | 1.00 | 2 |
| TZEI 2767 | TZE-W Pop DT C5 STR C5 | 0.03 | 0.97 | 2 |
| TZEI 2768 | TZE-W Pop DT C5 STR C5 | 0.02 | 0.98 | 2 |
| TZEI 2769 | TZE-W Pop DT C5 STR C5 | 0.00 | 1.00 | 2 |
| TZEI 2770 | TZE-W Pop DT C5 STR C5 | 0.07 | 0.93 | 2 |
| TZEI 2771 | TZE-W Pop DT C5 STR C5 | 0.01 | 0.99 | 2 |
| TZEI 2772 | TZE-W Pop DT C5 STR C5 | 0.01 | 1.00 | 2 |
| TZEI 2773 | TZE-W Pop DT C5 STR C5 | 0.02 | 0.98 | 2 |
| TZEI 2774 | TZE-W Pop DT C5 STR C5 | 0.11 | 0.89 | 2 |
| TZEI 2775 | TZE-W Pop DT C5 STR C5 | 0.03 | 0.97 | 2 |
| TZEI 2776 | TZE-W Pop DT C5 STR C5 | 0.03 | 0.97 | 2 |
| TZEI 2777 | TZE-W Pop DT C5 STR C5 | 0.02 | 0.98 | 2 |
| TZEI 2778 | TZE-W Pop DT C5 STR C5 | 0.06 | 0.94 | 2 |
| TZEI 2779 | TZE-W Pop DT C5 STR C5 | 0.02 | 0.98 | 2 |
| TZEI 2780 | TZE-W Pop DT C5 STR C5 | 0.00 | 1.00 | 2 |
| TZEI 2781 | TZE-W Pop DT C5 STR C5 | 0.02 | 0.98 | 2 |
| TZEI 2782 | TZE-W Pop DT C5 STR C5 | 0.01 | 0.99 | 2 |
| TZEI 2783 | TZE-W Pop DT C5 STR C5 | 0.00 | 1.00 | 2 |
| TZEI 2784 | TZE-W Pop DT C5 STR C5 | 0.07 | 0.93 | 2 |
| TZEI 2785 | TZE-W Pop DT C5 STR C5 | 0.09 | 0.91 | 2 |
| TZEI 2786 | TZE-W Pop DT C5 STR C5 | 0.03 | 0.97 | 2 |
| TZEI 2787 | TZE-W Pop DT C5 STR C5 | 0.03 | 0.98 | 2 |
| TZEI 2788 | TZE-W Pop DT C5 STR C5 | 0.00 | 1.00 | 2 |
| TZEI 2789 | TZE-W Pop DT C5 STR C5 | 0.16 | 0.84 | 2 |
| TZEI 2790 | TZE-W Pop DT C5 STR C5 | 0.14 | 0.86 | 2 |
| TZEI 2791 | TZE-W Pop DT C5 STR C5 | 0.00 | 1.00 | 2 |
| TZEI 2792 | TZE-W Pop DT C5 STR C5 | 0.02 | 0.98 | 2 |
| TZEI 2793 | TZE-W Pop DT C5 STR C5 | 0.00 | 1.00 | 2 |
| TZEI 2794 | TZE-W Pop DT C5 STR C5 | 0.02 | 0.98 | 2 |
| TZEI 2795 | TZE-W Pop DT C5 STR C5 | 0.06 | 0.94 | 2 |
| TZEI 2796 | TZE-W Pop DT C5 STR C5 | 0.03 | 0.98 | 2 |
| TZEI 2797 | TZE-W Pop DT C5 STR C5 | 0.01 | 1.00 | 2 |
| TZEI 2798 | TZE-W Pop DT C5 STR C5 | 0.01 | 0.99 | 2 |
| TZEI 2799 | TZE-W Pop DT C5 STR C5 | 0.06 | 0.94 | 2 |
| TZEI 2800 | TZE-W Pop DT C5 STR C5 | 0.01 | 0.99 | 2 |
| TZEI 2801 | TZE-W Pop DT C5 STR C5 | 0.17 | 0.83 | 2 |
| TZEI 2802 | TZE-W Pop DT C5 STR C5 | 0.00 | 1.00 | 2 |
| TZEI 2803 | TZE-W Pop DT C5 STR C5 | 0.00 | 1.00 | 2 |
| TZEI 2804 | TZE-W Pop DT C5 STR C5 | 0.00 | 1.00 | 2 |
| TZEI 2805 | TZE-W Pop DT C5 STR C5 | 0.00 | 1.00 | 2 |
| TZEI 2806 | TZE-W Pop DT C5 STR C5 | 0.02 | 0.98 | 2 |
| TZEI 2807 | TZE-W Pop DT C5 STR C5 | 0.01 | 0.99 | 2 |
| TZEI 2808 | TZE-W Pop DT C5 STR C5 | 0.04 | 0.96 | 2 |
| TZEI 2809 | TZE-W Pop DT C5 STR C5 | 0.00 | 1.00 | 2 |
| TZEI 2810 | TZE-W Pop DT C5 STR C5 | 0.01 | 1.00 | 2 |
| TZEI 2811 | TZE-W Pop DT C5 STR C5 | 0.10 | 0.90 | 2 |
| TZEI 2812 | TZE-W Pop DT C5 STR C5 | 0.01 | 0.99 | 2 |
| TZEI 2813 | TZE-W Pop DT C5 STR C5 | 0.02 | 0.98 | 2 |
| TZEI 2814 | TZE-W Pop DT C5 STR C5 | 0.00 | 1.00 | 2 |
| TZEI 2815 | TZE-W Pop DT C5 STR C5 | 0.00 | 1.00 | 2 |
| TZEI 2816 | TZE-W Pop DT C5 STR C5 | 0.00 | 1.00 | 2 |
| TZEI 2817 | TZE-W Pop DT C5 STR C5 | 0.00 | 1.00 | 2 |
| TZEI 2818 | TZE-W Pop DT C5 STR C5 | 0.00 | 1.00 | 2 |
| TZEI 2819 | TZE-W Pop DT C5 STR C5 | 0.06 | 0.94 | 2 |
| TZEI 2820 | TZE-W Pop DT C5 STR C5 | 0.07 | 0.93 | 2 |
| TZEI 2821 | TZE-W Pop DT C5 STR C5 | 0.01 | 0.99 | 2 |
| TZEI 2822 | TZE-W Pop DT C5 STR C5 | 0.00 | 1.00 | 2 |
| TZEI 2823 | TZE-W Pop DT C5 STR C5 | 0.04 | 0.96 | 2 |
| TZEI 2824 | TZE-W Pop DT C5 STR C5 | 0.07 | 0.94 | 2 |
| TZEI 2830 | TZE-W Pop DT C5 STR C5 | 0.02 | 0.98 | 2 |
| TZEI 2831 | TZE-W Pop DT C5 STR C5 | 0.00 | 1.00 | 2 |
| TZEI 2832 | TZE-W Pop DT C5 STR C5 | 0.02 | 0.98 | 2 |
| TZEI 2833 | TZE-W Pop DT C5 STR C5 | 0.00 | 1.00 | 2 |
| TZEI 2834 | TZE-W Pop DT C5 STR C5 | 0.01 | 0.99 | 2 |
| TZEI 2835 | TZE-W Pop DT C5 STR C5 | 0.01 | 1.00 | 2 |
| TZEI 2836 | TZE-W Pop DT C5 STR C5 | 0.01 | 0.99 | 2 |
| TZEI 2837 | TZE-W Pop DT C5 STR C5 | 0.01 | 0.99 | 2 |
| TZEI 2838 | TZE-W Pop DT C5 STR C5 | 0.00 | 1.00 | 2 |
| TZEI 2839 | TZE-W Pop DT C5 STR C5 | 0.03 | 0.97 | 2 |
| TZEI 2840 | TZE-W Pop DT C5 STR C5 | 0.11 | 0.89 | 2 |
| TZEI 2841 | TZE-W Pop DT C5 STR C5 | 0.07 | 0.93 | 2 |
| TZEI 2842 | TZE-W Pop DT C5 STR C5 | 0.02 | 0.98 | 2 |
| TZEI 2843 | TZE-W Pop DT C5 STR C5 | 0.00 | 1.00 | 2 |
| TZEI 2844 | TZE-W Pop DT C5 STR C5 | 0.00 | 1.00 | 2 |
| TZEI 2845 | TZE-W Pop DT C5 STR C5 | 0.00 | 1.00 | 2 |
| TZEI 2846 | TZE-W Pop DT C5 STR C5 | 0.01 | 1.00 | 2 |
| TZEI 2847 | TZE-W Pop DT C5 STR C5 | 0.00 | 1.00 | 2 |
| TZEI 2848 | TZE-W Pop DT C5 STR C5 | 0.01 | 0.99 | 2 |
| TZEI 2849 | TZE-W Pop DT C5 STR C5 | 0.01 | 0.99 | 2 |
| TZEI 2850 | TZE-W Pop DT C5 STR C5 | 0.00 | 1.00 | 2 |
| TZEI 2851 | TZE-W Pop DT C5 STR C5 | 0.06 | 0.95 | 2 |
| TZEI 2852 | TZE-W Pop DT C5 STR C5 | 0.08 | 0.92 | 2 |
| TZEI 2853 | TZE-W Pop DT C5 STR C5 | 0.11 | 0.89 | 2 |
| TZEI 2854 | TZE-W Pop DT C5 STR C5 | 0.01 | 0.99 | 2 |
| TZEI 2855 | TZE-W Pop DT C5 STR C5 | 0.00 | 1.00 | 2 |
| TZEI 2856 | TZE-W Pop DT C5 STR C5 | 0.00 | 1.00 | 2 |
| TZEI 2857 | TZE-W Pop DT C5 STR C5 | 0.00 | 1.00 | 2 |
| TZEI 2858 | TZE-W Pop DT C5 STR C5 | 0.01 | 0.99 | 2 |
| TZEI 2859 | TZE-W Pop DT C5 STR C5 | 0.01 | 0.99 | 2 |
| TZEI 2860 | TZE-W Pop DT C5 STR C5 | 0.02 | 0.98 | 2 |
| TZEI 2861 | TZE-W Pop DT C5 STR C5 | 0.01 | 1.00 | 2 |
| TZEI 2862 | TZE-W Pop DT C5 STR C5 | 0.00 | 1.00 | 2 |
| TZEI 2863 | TZE-W Pop DT C5 STR C5 | 0.02 | 0.98 | 2 |
| TZEI 2864 | TZE-W Pop DT C5 STR C5 | 0.03 | 0.98 | 2 |
| TZEI 2865 | TZE-W Pop DT C5 STR C5 | 0.12 | 0.88 | 2 |
| TZEI 2866 | TZE-W Pop DT C5 STR C5 | 0.01 | 0.99 | 2 |
| TZEI 2867 | TZE-W Pop DT C5 STR C5 | 0.00 | 1.00 | 2 |
| TZEI 2868 | TZE-W Pop DT C5 STR C5 | 0.00 | 1.00 | 2 |
| TZEI 2869 | TZE-W Pop DT C5 STR C5 | 0.04 | 0.96 | 2 |
| TZEI 2870 | TZE-W Pop DT C5 STR C5 | 0.10 | 0.90 | 2 |
| TZEI 2871 | TZE-W Pop DT C5 STR C5 | 0.04 | 0.96 | 2 |
| TZEI 2872 | TZE-W Pop DT C5 STR C5 | 0.03 | 0.97 | 2 |
| TZEI 2873 | TZE-W Pop DT C5 STR C5 | 0.00 | 1.00 | 2 |
| TZEI 2874 | TZE-W Pop DT C5 STR C5 | 0.03 | 0.97 | 2 |
| TZEI 2875 | TZE-W Pop DT C5 STR C5 | 0.02 | 0.98 | 2 |
| TZEI 2232 | TZEI 65 x ENT 11 | 0.41 | 0.59 | admix |
| TZEI 2236 | TZEI 65 x ENT 11 | 0.39 | 0.62 | admix |
| TZEI 2237 | TZEI 65 x ENT 11 | 0.33 | 0.67 | admix |
| TZEI 2244 | TZEI 65 x ENT 11 | 0.40 | 0.61 | admix |
| TZEI 2248 | TZEI 65 x ENT 11 | 0.37 | 0.63 | admix |
| TZEI 2249 | TZEI 65 x ENT 11 | 0.34 | 0.66 | admix |
| TZEI 2250 | TZEI 65 x ENT 11 | 0.21 | 0.79 | admix |
| TZEI 2257 | TZEI 65 x ENT 11 | 0.43 | 0.57 | admix |
| TZEI 2260 | TZEI 65 x ENT 11 | 0.73 | 0.27 | admix |
| TZEI 2261 | TZEI 65 x ENT 11 | 0.41 | 0.59 | admix |
| TZEI 2262 | TZEI 65 x ENT 11 | 0.37 | 0.63 | admix |
| TZEI 2269 | TZEI 65 x ENT 11 | 0.39 | 0.61 | admix |
| TZEI 2272 | TZEI 65 x ENT 11 | 0.35 | 0.65 | admix |
| TZEI 2280 | TZEI 65 x ENT 11 | 0.44 | 0.56 | admix |
| TZEI 2282 | TZEI 65 x ENT 11 | 0.65 | 0.35 | admix |
| TZEI 2283 | TZEI 65 x ENT 11 | 0.40 | 0.60 | admix |
| TZEI 2284 | TZEI 65 x ENT 11 | 0.36 | 0.64 | admix |
| TZEI 2454 | DTE STR-W Syn Pop C4 | 0.23 | 0.77 | admix |
| TZEI 2458 | DTE STR-W Syn Pop C4 | 0.22 | 0.78 | admix |
| TZEI 2487 | DTE STR-W Syn Pop C4 | 0.24 | 0.76 | admix |
| TZEI 2492 | DTE STR-W Syn Pop C4 | 0.38 | 0.62 | admix |
| TZEI 2504 | DTE STR-W Syn Pop C4 | 0.40 | 0.60 | admix |
| TZEI 2527 | DTE STR-W Syn Pop C4 | 0.24 | 0.77 | admix |
| TZEI 2535 | DTE STR-W Syn Pop C4 | 0.44 | 0.56 | admix |
| TZEI 2539 | DTE STR-W Syn Pop C4 | 0.43 | 0.57 | admix |
| TZEI 2540 | DTE STR-W Syn Pop C4 | 0.38 | 0.62 | admix |
| TZEI 2541 | DTE STR-W Syn Pop C4 | 0.28 | 0.72 | admix |
| TZEI 2547 | DTE STR-W Syn Pop C4 | 0.39 | 0.61 | admix |
| TZEI 2551 | DTE STR-W Syn Pop C4 | 0.42 | 0.58 | admix |
| TZEI 2552 | DTE STR-W Syn Pop C4 | 0.42 | 0.58 | admix |
| TZEI 2553 | DTE STR-W Syn Pop C4 | 0.25 | 0.75 | admix |
| TZEI 2558 | DTE STR-W Syn Pop C4 | 0.38 | 0.62 | admix |
| TZEI 2562 | DTE STR-W Syn Pop C4 | 0.36 | 0.64 | admix |
| TZEI 2563 | DTE STR-W Syn Pop C4 | 0.36 | 0.64 | admix |
| TZEI 2564 | DTE STR-W Syn Pop C4 | 0.33 | 0.67 | admix |
| TZEI 2702 | TZE-W Pop DT C5 STR C5 | 0.21 | 0.79 | admix |

Table S2: Population stratification based on Bayesian statistics approach implemented in STRUCTURE revealed six genetic groups with 33 % level of admixture

| Geno | Q1 | Q2 | Q3 | Q4 | Q5 | Q6 | Cluster |
| --- | --- | --- | --- | --- | --- | --- | --- |
| TZEI 2440 | 1.00 | 0.00 | 0.00 | 0.00 | 0.00 | 0.00 | 1.00 |
| TZEI 2444 | 1.00 | 0.00 | 0.00 | 0.00 | 0.00 | 0.00 | 1.00 |
| TZEI 2452 | 1.00 | 0.00 | 0.00 | 0.00 | 0.00 | 0.00 | 1.00 |
| TZEI 2456 | 1.00 | 0.00 | 0.00 | 0.00 | 0.00 | 0.00 | 1.00 |
| TZEI 2464 | 1.00 | 0.00 | 0.00 | 0.00 | 0.00 | 0.00 | 1.00 |
| TZEI 2468 | 1.00 | 0.00 | 0.00 | 0.00 | 0.00 | 0.00 | 1.00 |
| TZEI 2476 | 1.00 | 0.00 | 0.00 | 0.00 | 0.00 | 0.00 | 1.00 |
| TZEI 2480 | 1.00 | 0.00 | 0.00 | 0.00 | 0.00 | 0.00 | 1.00 |
| TZEI 2489 | 1.00 | 0.00 | 0.00 | 0.00 | 0.00 | 0.00 | 1.00 |
| TZEI 2493 | 1.00 | 0.00 | 0.00 | 0.00 | 0.00 | 0.00 | 1.00 |
| TZEI 2501 | 0.99 | 0.00 | 0.00 | 0.00 | 0.00 | 0.00 | 1.00 |
| TZEI 2513 | 1.00 | 0.00 | 0.00 | 0.00 | 0.00 | 0.00 | 1.00 |
| TZEI 2524 | 1.00 | 0.00 | 0.00 | 0.00 | 0.00 | 0.00 | 1.00 |
| TZEI 2854 | 1.00 | 0.00 | 0.00 | 0.00 | 0.00 | 0.00 | 1.00 |
| TZEI 2432 | 0.00 | 1.00 | 0.00 | 0.00 | 0.00 | 0.00 | 2.00 |
| TZEI 2793 | 0.00 | 1.00 | 0.00 | 0.00 | 0.00 | 0.00 | 2.00 |
| TZEI 2804 | 0.00 | 1.00 | 0.00 | 0.00 | 0.00 | 0.00 | 2.00 |
| TZEI 2805 | 0.00 | 1.00 | 0.00 | 0.00 | 0.00 | 0.00 | 2.00 |
| TZEI 2816 | 0.00 | 1.00 | 0.00 | 0.00 | 0.00 | 0.00 | 2.00 |
| TZEI 2817 | 0.00 | 0.99 | 0.00 | 0.00 | 0.00 | 0.00 | 2.00 |
| TZEI 2833 | 0.00 | 0.99 | 0.00 | 0.01 | 0.00 | 0.00 | 2.00 |
| TZEI 2844 | 0.00 | 1.00 | 0.00 | 0.00 | 0.00 | 0.00 | 2.00 |
| TZEI 2845 | 0.00 | 1.00 | 0.00 | 0.00 | 0.00 | 0.00 | 2.00 |
| TZEI 2856 | 0.00 | 1.00 | 0.00 | 0.00 | 0.00 | 0.00 | 2.00 |
| TZEI 2857 | 0.00 | 1.00 | 0.00 | 0.00 | 0.00 | 0.00 | 2.00 |
| TZEI 2867 | 0.00 | 1.00 | 0.00 | 0.00 | 0.00 | 0.00 | 2.00 |
| TZEI 2868 | 0.01 | 0.99 | 0.00 | 0.00 | 0.00 | 0.00 | 2.00 |
| TZEI 2231 | 0.06 | 0.00 | 0.93 | 0.00 | 0.00 | 0.00 | 3.00 |
| TZEI 2233 | 0.00 | 0.00 | 0.99 | 0.00 | 0.00 | 0.00 | 3.00 |
| TZEI 2234 | 0.00 | 0.00 | 1.00 | 0.00 | 0.00 | 0.00 | 3.00 |
| TZEI 2235 | 0.00 | 0.00 | 0.95 | 0.01 | 0.00 | 0.04 | 3.00 |
| TZEI 2239 | 0.00 | 0.00 | 1.00 | 0.00 | 0.00 | 0.00 | 3.00 |
| TZEI 2240 | 0.00 | 0.00 | 1.00 | 0.00 | 0.00 | 0.00 | 3.00 |
| TZEI 2241 | 0.00 | 0.02 | 0.87 | 0.01 | 0.01 | 0.08 | 3.00 |
| TZEI 2242 | 0.00 | 0.00 | 1.00 | 0.00 | 0.00 | 0.00 | 3.00 |
| TZEI 2243 | 0.00 | 0.00 | 0.97 | 0.01 | 0.00 | 0.01 | 3.00 |
| TZEI 2245 | 0.02 | 0.00 | 0.96 | 0.00 | 0.00 | 0.01 | 3.00 |
| TZEI 2246 | 0.00 | 0.00 | 0.99 | 0.00 | 0.00 | 0.00 | 3.00 |
| TZEI 2247 | 0.00 | 0.00 | 0.97 | 0.00 | 0.00 | 0.03 | 3.00 |
| TZEI 2252 | 0.00 | 0.00 | 1.00 | 0.00 | 0.00 | 0.00 | 3.00 |
| TZEI 2253 | 0.00 | 0.00 | 1.00 | 0.00 | 0.00 | 0.00 | 3.00 |
| TZEI 2254 | 0.00 | 0.00 | 1.00 | 0.00 | 0.00 | 0.00 | 3.00 |
| TZEI 2255 | 0.00 | 0.00 | 0.99 | 0.00 | 0.00 | 0.00 | 3.00 |
| TZEI 2256 | 0.04 | 0.00 | 0.95 | 0.00 | 0.00 | 0.00 | 3.00 |
| TZEI 2258 | 0.00 | 0.00 | 1.00 | 0.00 | 0.00 | 0.00 | 3.00 |
| TZEI 2259 | 0.00 | 0.00 | 1.00 | 0.00 | 0.00 | 0.00 | 3.00 |
| TZEI 2264 | 0.00 | 0.00 | 1.00 | 0.00 | 0.00 | 0.00 | 3.00 |
| TZEI 2265 | 0.00 | 0.00 | 1.00 | 0.00 | 0.00 | 0.00 | 3.00 |
| TZEI 2266 | 0.00 | 0.03 | 0.91 | 0.03 | 0.00 | 0.03 | 3.00 |
| TZEI 2267 | 0.00 | 0.00 | 1.00 | 0.00 | 0.00 | 0.00 | 3.00 |
| TZEI 2268 | 0.00 | 0.00 | 0.98 | 0.00 | 0.00 | 0.02 | 3.00 |
| TZEI 2270 | 0.00 | 0.00 | 0.99 | 0.00 | 0.00 | 0.00 | 3.00 |
| TZEI 2271 | 0.00 | 0.00 | 1.00 | 0.00 | 0.00 | 0.00 | 3.00 |
| TZEI 2275 | 0.00 | 0.00 | 1.00 | 0.00 | 0.00 | 0.00 | 3.00 |
| TZEI 2276 | 0.00 | 0.00 | 1.00 | 0.00 | 0.00 | 0.00 | 3.00 |
| TZEI 2277 | 0.00 | 0.00 | 1.00 | 0.00 | 0.00 | 0.00 | 3.00 |
| TZEI 2278 | 0.00 | 0.00 | 1.00 | 0.00 | 0.00 | 0.00 | 3.00 |
| TZEI 2279 | 0.04 | 0.00 | 0.94 | 0.00 | 0.01 | 0.01 | 3.00 |
| TZEI 2281 | 0.00 | 0.00 | 0.99 | 0.00 | 0.00 | 0.00 | 3.00 |
| TZEI 2286 | 0.00 | 0.00 | 1.00 | 0.00 | 0.00 | 0.00 | 3.00 |
| TZEI 2287 | 0.00 | 0.00 | 1.00 | 0.00 | 0.00 | 0.00 | 3.00 |
| TZEI 2288 | 0.00 | 0.00 | 1.00 | 0.00 | 0.00 | 0.00 | 3.00 |
| TZEI 2289 | 0.00 | 0.00 | 1.00 | 0.00 | 0.00 | 0.00 | 3.00 |
| TZEI 2290 | 0.00 | 0.00 | 1.00 | 0.00 | 0.00 | 0.00 | 3.00 |
| TZEI 2536 | 0.00 | 0.00 | 1.00 | 0.00 | 0.00 | 0.00 | 3.00 |
| TZEI 2537 | 0.00 | 0.00 | 0.95 | 0.00 | 0.00 | 0.05 | 3.00 |
| TZEI 2538 | 0.00 | 0.00 | 1.00 | 0.00 | 0.00 | 0.00 | 3.00 |
| TZEI 2543 | 0.00 | 0.00 | 1.00 | 0.00 | 0.00 | 0.00 | 3.00 |
| TZEI 2544 | 0.00 | 0.00 | 0.99 | 0.00 | 0.00 | 0.01 | 3.00 |
| TZEI 2545 | 0.00 | 0.00 | 1.00 | 0.00 | 0.00 | 0.00 | 3.00 |
| TZEI 2546 | 0.00 | 0.00 | 0.94 | 0.00 | 0.00 | 0.06 | 3.00 |
| TZEI 2548 | 0.00 | 0.00 | 1.00 | 0.00 | 0.00 | 0.00 | 3.00 |
| TZEI 2549 | 0.00 | 0.00 | 1.00 | 0.00 | 0.00 | 0.00 | 3.00 |
| TZEI 2550 | 0.00 | 0.00 | 0.95 | 0.00 | 0.01 | 0.04 | 3.00 |
| TZEI 2555 | 0.00 | 0.00 | 1.00 | 0.00 | 0.00 | 0.00 | 3.00 |
| TZEI 2556 | 0.00 | 0.00 | 0.99 | 0.00 | 0.00 | 0.00 | 3.00 |
| TZEI 2557A | 0.00 | 0.00 | 1.00 | 0.00 | 0.00 | 0.00 | 3.00 |
| TZEI 2557B | 0.00 | 0.00 | 0.95 | 0.00 | 0.00 | 0.05 | 3.00 |
| TZEI 2559 | 0.00 | 0.00 | 0.99 | 0.00 | 0.00 | 0.00 | 3.00 |
| TZEI 2560 | 0.00 | 0.00 | 0.95 | 0.00 | 0.00 | 0.05 | 3.00 |
| TZEI 2561 | 0.00 | 0.00 | 1.00 | 0.00 | 0.00 | 0.00 | 3.00 |
| TZEI 2566 | 0.00 | 0.00 | 1.00 | 0.00 | 0.00 | 0.00 | 3.00 |
| TZEI 2567 | 0.00 | 0.00 | 0.99 | 0.01 | 0.00 | 0.00 | 3.00 |
| TZEI 2568 | 0.00 | 0.00 | 0.99 | 0.00 | 0.00 | 0.01 | 3.00 |
| TZEI 2430 | 0.01 | 0.00 | 0.00 | 0.98 | 0.00 | 0.00 | 4.00 |
| TZEI 2437 | 0.03 | 0.02 | 0.01 | 0.87 | 0.01 | 0.06 | 4.00 |
| TZEI 2438 | 0.00 | 0.06 | 0.01 | 0.93 | 0.01 | 0.00 | 4.00 |
| TZEI 2439 | 0.00 | 0.02 | 0.00 | 0.96 | 0.01 | 0.00 | 4.00 |
| TZEI 2471 | 0.10 | 0.00 | 0.08 | 0.80 | 0.01 | 0.01 | 4.00 |
| TZEI 2695 | 0.00 | 0.10 | 0.00 | 0.90 | 0.00 | 0.00 | 4.00 |
| TZEI 2696 | 0.06 | 0.01 | 0.02 | 0.87 | 0.03 | 0.01 | 4.00 |
| TZEI 2697 | 0.01 | 0.02 | 0.01 | 0.96 | 0.01 | 0.00 | 4.00 |
| TZEI 2699 | 0.00 | 0.12 | 0.01 | 0.84 | 0.03 | 0.00 | 4.00 |
| TZEI 2700 | 0.01 | 0.00 | 0.01 | 0.93 | 0.01 | 0.04 | 4.00 |
| TZEI 2701 | 0.05 | 0.00 | 0.00 | 0.94 | 0.01 | 0.00 | 4.00 |
| TZEI 2704 | 0.01 | 0.01 | 0.00 | 0.88 | 0.10 | 0.00 | 4.00 |
| TZEI 2705 | 0.02 | 0.01 | 0.05 | 0.92 | 0.01 | 0.00 | 4.00 |
| TZEI 2706 | 0.05 | 0.00 | 0.00 | 0.94 | 0.01 | 0.00 | 4.00 |
| TZEI 2707 | 0.00 | 0.13 | 0.00 | 0.87 | 0.00 | 0.00 | 4.00 |
| TZEI 2710 | 0.00 | 0.00 | 0.00 | 0.89 | 0.10 | 0.00 | 4.00 |
| TZEI 2711 | 0.01 | 0.07 | 0.01 | 0.81 | 0.05 | 0.05 | 4.00 |
| TZEI 2712 | 0.01 | 0.03 | 0.05 | 0.82 | 0.01 | 0.08 | 4.00 |
| TZEI 2713 | 0.01 | 0.00 | 0.01 | 0.92 | 0.00 | 0.05 | 4.00 |
| TZEI 2714 | 0.09 | 0.00 | 0.00 | 0.90 | 0.01 | 0.00 | 4.00 |
| TZEI 2716 | 0.00 | 0.01 | 0.00 | 0.99 | 0.00 | 0.00 | 4.00 |
| TZEI 2717 | 0.01 | 0.01 | 0.00 | 0.87 | 0.11 | 0.00 | 4.00 |
| TZEI 2718 | 0.01 | 0.01 | 0.07 | 0.87 | 0.00 | 0.05 | 4.00 |
| TZEI 2719 | 0.05 | 0.00 | 0.02 | 0.93 | 0.00 | 0.00 | 4.00 |
| TZEI 2720 | 0.05 | 0.12 | 0.01 | 0.82 | 0.01 | 0.00 | 4.00 |
| TZEI 2722 | 0.02 | 0.01 | 0.00 | 0.96 | 0.00 | 0.01 | 4.00 |
| TZEI 2724 | 0.02 | 0.00 | 0.00 | 0.95 | 0.03 | 0.00 | 4.00 |
| TZEI 2725A | 0.00 | 0.10 | 0.00 | 0.89 | 0.00 | 0.00 | 4.00 |
| TZEI 2725B | 0.01 | 0.02 | 0.00 | 0.94 | 0.03 | 0.00 | 4.00 |
| TZEI 2726A | 0.00 | 0.00 | 0.04 | 0.92 | 0.01 | 0.03 | 4.00 |
| TZEI 2726B | 0.10 | 0.00 | 0.00 | 0.89 | 0.00 | 0.00 | 4.00 |
| TZEI 2727 | 0.07 | 0.03 | 0.00 | 0.83 | 0.06 | 0.00 | 4.00 |
| TZEI 2728 | 0.00 | 0.01 | 0.00 | 0.98 | 0.00 | 0.00 | 4.00 |
| TZEI 2729 | 0.02 | 0.05 | 0.00 | 0.92 | 0.00 | 0.01 | 4.00 |
| TZEI 2730 | 0.02 | 0.01 | 0.07 | 0.89 | 0.01 | 0.00 | 4.00 |
| TZEI 2731 | 0.03 | 0.00 | 0.03 | 0.94 | 0.00 | 0.00 | 4.00 |
| TZEI 2734 | 0.00 | 0.05 | 0.04 | 0.88 | 0.01 | 0.03 | 4.00 |
| TZEI 2735A | 0.01 | 0.09 | 0.00 | 0.89 | 0.01 | 0.00 | 4.00 |
| TZEI 2735B | 0.01 | 0.01 | 0.00 | 0.93 | 0.05 | 0.00 | 4.00 |
| TZEI 2736A | 0.00 | 0.06 | 0.00 | 0.93 | 0.00 | 0.00 | 4.00 |
| TZEI 2736B | 0.10 | 0.00 | 0.00 | 0.89 | 0.00 | 0.00 | 4.00 |
| TZEI 2738 | 0.00 | 0.01 | 0.00 | 0.98 | 0.00 | 0.01 | 4.00 |
| TZEI 2739 | 0.06 | 0.08 | 0.01 | 0.84 | 0.01 | 0.00 | 4.00 |
| TZEI 2740 | 0.03 | 0.00 | 0.01 | 0.95 | 0.01 | 0.00 | 4.00 |
| TZEI 2741A | 0.04 | 0.01 | 0.01 | 0.86 | 0.09 | 0.00 | 4.00 |
| TZEI 2741B | 0.01 | 0.10 | 0.01 | 0.87 | 0.02 | 0.00 | 4.00 |
| TZEI 2743 | 0.00 | 0.02 | 0.02 | 0.90 | 0.00 | 0.06 | 4.00 |
| TZEI 2744 | 0.00 | 0.06 | 0.00 | 0.91 | 0.00 | 0.02 | 4.00 |
| TZEI 2745 | 0.00 | 0.03 | 0.00 | 0.94 | 0.03 | 0.00 | 4.00 |
| TZEI 2746A | 0.01 | 0.04 | 0.00 | 0.95 | 0.00 | 0.00 | 4.00 |
| TZEI 2746B | 0.10 | 0.00 | 0.00 | 0.90 | 0.00 | 0.00 | 4.00 |
| TZEI 2747 | 0.04 | 0.00 | 0.11 | 0.82 | 0.03 | 0.00 | 4.00 |
| TZEI 2748 | 0.00 | 0.04 | 0.00 | 0.96 | 0.00 | 0.00 | 4.00 |
| TZEI 2749 | 0.03 | 0.08 | 0.04 | 0.85 | 0.00 | 0.00 | 4.00 |
| TZEI 2750 | 0.03 | 0.00 | 0.05 | 0.91 | 0.01 | 0.00 | 4.00 |
| TZEI 2751 | 0.05 | 0.01 | 0.01 | 0.86 | 0.08 | 0.00 | 4.00 |
| TZEI 2752 | 0.01 | 0.05 | 0.00 | 0.90 | 0.04 | 0.00 | 4.00 |
| TZEI 2753 | 0.00 | 0.15 | 0.01 | 0.84 | 0.00 | 0.00 | 4.00 |
| TZEI 2754 | 0.03 | 0.05 | 0.00 | 0.91 | 0.01 | 0.00 | 4.00 |
| TZEI 2755 | 0.02 | 0.02 | 0.00 | 0.95 | 0.00 | 0.01 | 4.00 |
| TZEI 2756 | 0.00 | 0.04 | 0.00 | 0.92 | 0.03 | 0.00 | 4.00 |
| TZEI 2757 | 0.00 | 0.09 | 0.00 | 0.90 | 0.00 | 0.00 | 4.00 |
| TZEI 2758 | 0.09 | 0.00 | 0.00 | 0.91 | 0.00 | 0.00 | 4.00 |
| TZEI 2760 | 0.00 | 0.01 | 0.00 | 0.98 | 0.00 | 0.00 | 4.00 |
| TZEI 2763 | 0.04 | 0.00 | 0.06 | 0.88 | 0.02 | 0.00 | 4.00 |
| TZEI 2764 | 0.00 | 0.09 | 0.00 | 0.81 | 0.09 | 0.00 | 4.00 |
| TZEI 2765 | 0.00 | 0.13 | 0.00 | 0.85 | 0.01 | 0.00 | 4.00 |
| TZEI 2766 | 0.01 | 0.04 | 0.00 | 0.93 | 0.02 | 0.00 | 4.00 |
| TZEI 2768 | 0.00 | 0.06 | 0.00 | 0.93 | 0.00 | 0.00 | 4.00 |
| TZEI 2769 | 0.08 | 0.00 | 0.00 | 0.91 | 0.00 | 0.00 | 4.00 |
| TZEI 2770 | 0.03 | 0.01 | 0.06 | 0.84 | 0.06 | 0.01 | 4.00 |
| TZEI 2771 | 0.00 | 0.11 | 0.00 | 0.87 | 0.00 | 0.02 | 4.00 |
| TZEI 2775 | 0.00 | 0.11 | 0.02 | 0.84 | 0.03 | 0.00 | 4.00 |
| TZEI 2776 | 0.01 | 0.03 | 0.00 | 0.82 | 0.06 | 0.07 | 4.00 |
| TZEI 2777 | 0.00 | 0.15 | 0.01 | 0.83 | 0.01 | 0.00 | 4.00 |
| TZEI 2778 | 0.01 | 0.00 | 0.01 | 0.92 | 0.01 | 0.05 | 4.00 |
| TZEI 2779 | 0.01 | 0.08 | 0.01 | 0.90 | 0.00 | 0.00 | 4.00 |
| TZEI 2780 | 0.09 | 0.00 | 0.00 | 0.91 | 0.00 | 0.00 | 4.00 |
| TZEI 2781 | 0.00 | 0.11 | 0.00 | 0.81 | 0.06 | 0.02 | 4.00 |
| TZEI 2782 | 0.01 | 0.00 | 0.00 | 0.88 | 0.11 | 0.00 | 4.00 |
| TZEI 2785 | 0.01 | 0.00 | 0.04 | 0.80 | 0.14 | 0.00 | 4.00 |
| TZEI 2786 | 0.06 | 0.07 | 0.02 | 0.81 | 0.02 | 0.01 | 4.00 |
| TZEI 2787 | 0.04 | 0.01 | 0.01 | 0.89 | 0.06 | 0.00 | 4.00 |
| TZEI 2788 | 0.00 | 0.15 | 0.00 | 0.83 | 0.01 | 0.00 | 4.00 |
| TZEI 2790 | 0.00 | 0.00 | 0.01 | 0.82 | 0.06 | 0.10 | 4.00 |
| TZEI 2791 | 0.01 | 0.01 | 0.00 | 0.99 | 0.00 | 0.00 | 4.00 |
| TZEI 2794 | 0.08 | 0.00 | 0.02 | 0.86 | 0.03 | 0.00 | 4.00 |
| TZEI 2795 | 0.01 | 0.01 | 0.04 | 0.84 | 0.10 | 0.00 | 4.00 |
| TZEI 2796 | 0.02 | 0.03 | 0.01 | 0.89 | 0.01 | 0.05 | 4.00 |
| TZEI 2797 | 0.00 | 0.11 | 0.00 | 0.88 | 0.01 | 0.00 | 4.00 |
| TZEI 2798 | 0.07 | 0.09 | 0.00 | 0.81 | 0.01 | 0.02 | 4.00 |
| TZEI 2799 | 0.05 | 0.02 | 0.04 | 0.84 | 0.05 | 0.00 | 4.00 |
| TZEI 2800 | 0.00 | 0.14 | 0.00 | 0.81 | 0.05 | 0.00 | 4.00 |
| TZEI 2802 | 0.00 | 0.00 | 0.00 | 1.00 | 0.00 | 0.00 | 4.00 |
| TZEI 2803 | 0.00 | 0.00 | 0.00 | 1.00 | 0.00 | 0.00 | 4.00 |
| TZEI 2806 | 0.04 | 0.00 | 0.01 | 0.89 | 0.06 | 0.00 | 4.00 |
| TZEI 2809 | 0.00 | 0.12 | 0.00 | 0.87 | 0.00 | 0.00 | 4.00 |
| TZEI 2810 | 0.02 | 0.14 | 0.00 | 0.80 | 0.04 | 0.00 | 4.00 |
| TZEI 2811 | 0.02 | 0.01 | 0.10 | 0.86 | 0.02 | 0.00 | 4.00 |
| TZEI 2812 | 0.00 | 0.12 | 0.00 | 0.82 | 0.05 | 0.00 | 4.00 |
| TZEI 2814 | 0.00 | 0.00 | 0.00 | 1.00 | 0.00 | 0.00 | 4.00 |
| TZEI 2815 | 0.00 | 0.00 | 0.00 | 1.00 | 0.00 | 0.00 | 4.00 |
| TZEI 2819 | 0.04 | 0.01 | 0.02 | 0.81 | 0.12 | 0.00 | 4.00 |
| TZEI 2821 | 0.00 | 0.12 | 0.00 | 0.88 | 0.00 | 0.00 | 4.00 |
| TZEI 2822 | 0.00 | 0.11 | 0.00 | 0.89 | 0.00 | 0.00 | 4.00 |
| TZEI 2824 | 0.05 | 0.00 | 0.04 | 0.88 | 0.01 | 0.02 | 4.00 |
| TZEI 2831 | 0.00 | 0.00 | 0.00 | 1.00 | 0.00 | 0.00 | 4.00 |
| TZEI 2832 | 0.05 | 0.02 | 0.02 | 0.90 | 0.01 | 0.01 | 4.00 |
| TZEI 2836 | 0.03 | 0.00 | 0.00 | 0.87 | 0.10 | 0.00 | 4.00 |
| TZEI 2837 | 0.04 | 0.11 | 0.00 | 0.81 | 0.02 | 0.02 | 4.00 |
| TZEI 2838 | 0.00 | 0.12 | 0.00 | 0.88 | 0.00 | 0.00 | 4.00 |
| TZEI 2839 | 0.07 | 0.03 | 0.02 | 0.88 | 0.00 | 0.00 | 4.00 |
| TZEI 2841 | 0.03 | 0.05 | 0.07 | 0.83 | 0.01 | 0.01 | 4.00 |
| TZEI 2843 | 0.00 | 0.00 | 0.00 | 1.00 | 0.00 | 0.00 | 4.00 |
| TZEI 2849 | 0.00 | 0.16 | 0.00 | 0.83 | 0.00 | 0.00 | 4.00 |
| TZEI 2850 | 0.00 | 0.11 | 0.00 | 0.87 | 0.00 | 0.01 | 4.00 |
| TZEI 2851 | 0.03 | 0.01 | 0.06 | 0.87 | 0.03 | 0.00 | 4.00 |
| TZEI 2852 | 0.00 | 0.01 | 0.06 | 0.93 | 0.00 | 0.00 | 4.00 |
| TZEI 2855 | 0.00 | 0.00 | 0.00 | 0.99 | 0.00 | 0.00 | 4.00 |
| TZEI 2859 | 0.08 | 0.09 | 0.00 | 0.82 | 0.00 | 0.00 | 4.00 |
| TZEI 2860 | 0.10 | 0.01 | 0.01 | 0.85 | 0.02 | 0.02 | 4.00 |
| TZEI 2861 | 0.01 | 0.13 | 0.00 | 0.86 | 0.00 | 0.00 | 4.00 |
| TZEI 2862 | 0.00 | 0.10 | 0.00 | 0.89 | 0.00 | 0.00 | 4.00 |
| TZEI 2864 | 0.01 | 0.05 | 0.02 | 0.92 | 0.00 | 0.00 | 4.00 |
| TZEI 2866 | 0.01 | 0.00 | 0.00 | 0.99 | 0.00 | 0.01 | 4.00 |
| TZEI 2871 | 0.10 | 0.01 | 0.01 | 0.80 | 0.04 | 0.04 | 4.00 |
| TZEI 2872 | 0.00 | 0.10 | 0.01 | 0.89 | 0.00 | 0.00 | 4.00 |
| TZEI 2873 | 0.00 | 0.11 | 0.00 | 0.88 | 0.00 | 0.00 | 4.00 |
| TZEI 2874 | 0.01 | 0.02 | 0.01 | 0.94 | 0.01 | 0.00 | 4.00 |
| TZEI 2875 | 0.03 | 0.02 | 0.01 | 0.90 | 0.04 | 0.00 | 4.00 |
| TZEI 2449 | 0.00 | 0.00 | 0.00 | 0.00 | 1.00 | 0.00 | 5.00 |
| TZEI 2450 | 0.00 | 0.00 | 0.00 | 0.00 | 1.00 | 0.00 | 5.00 |
| TZEI 2460 | 0.00 | 0.00 | 0.00 | 0.00 | 0.99 | 0.00 | 5.00 |
| TZEI 2461 | 0.00 | 0.00 | 0.00 | 0.00 | 1.00 | 0.00 | 5.00 |
| TZEI 2462 | 0.00 | 0.00 | 0.00 | 0.00 | 1.00 | 0.00 | 5.00 |
| TZEI 2472 | 0.00 | 0.00 | 0.00 | 0.00 | 1.00 | 0.00 | 5.00 |
| TZEI 2473 | 0.00 | 0.00 | 0.00 | 0.00 | 1.00 | 0.00 | 5.00 |
| TZEI 2485 | 0.00 | 0.00 | 0.00 | 0.00 | 0.99 | 0.00 | 5.00 |
| TZEI 2498 | 0.00 | 0.00 | 0.01 | 0.00 | 0.99 | 0.00 | 5.00 |
| TZEI 2510 | 0.00 | 0.00 | 0.00 | 0.00 | 1.00 | 0.00 | 5.00 |
| TZEI 2521 | 0.00 | 0.00 | 0.00 | 0.00 | 1.00 | 0.00 | 5.00 |
| TZEI 2532 | 0.00 | 0.00 | 0.00 | 0.00 | 1.00 | 0.00 | 5.00 |
| TZEI 2232 | 0.00 | 0.00 | 0.00 | 0.00 | 0.00 | 1.00 | 6.00 |
| TZEI 2236 | 0.00 | 0.00 | 0.00 | 0.00 | 0.00 | 1.00 | 6.00 |
| TZEI 2237 | 0.00 | 0.00 | 0.00 | 0.00 | 0.00 | 1.00 | 6.00 |
| TZEI 2244 | 0.00 | 0.00 | 0.00 | 0.00 | 0.00 | 1.00 | 6.00 |
| TZEI 2248 | 0.00 | 0.00 | 0.00 | 0.00 | 0.00 | 1.00 | 6.00 |
| TZEI 2249 | 0.00 | 0.00 | 0.00 | 0.00 | 0.00 | 1.00 | 6.00 |
| TZEI 2257 | 0.00 | 0.00 | 0.00 | 0.00 | 0.00 | 1.00 | 6.00 |
| TZEI 2261 | 0.00 | 0.00 | 0.00 | 0.00 | 0.00 | 1.00 | 6.00 |
| TZEI 2262 | 0.00 | 0.00 | 0.00 | 0.00 | 0.00 | 1.00 | 6.00 |
| TZEI 2269 | 0.00 | 0.00 | 0.00 | 0.00 | 0.00 | 1.00 | 6.00 |
| TZEI 2272 | 0.00 | 0.00 | 0.00 | 0.00 | 0.00 | 1.00 | 6.00 |
| TZEI 2280 | 0.00 | 0.00 | 0.00 | 0.00 | 0.00 | 1.00 | 6.00 |
| TZEI 2283 | 0.00 | 0.00 | 0.00 | 0.00 | 0.00 | 1.00 | 6.00 |
| TZEI 2284 | 0.00 | 0.00 | 0.00 | 0.00 | 0.00 | 1.00 | 6.00 |
| TZEI 2492 | 0.01 | 0.02 | 0.02 | 0.01 | 0.00 | 0.95 | 6.00 |
| TZEI 2504 | 0.01 | 0.00 | 0.08 | 0.00 | 0.00 | 0.91 | 6.00 |
| TZEI 2535 | 0.00 | 0.00 | 0.00 | 0.00 | 0.00 | 1.00 | 6.00 |
| TZEI 2539 | 0.00 | 0.00 | 0.00 | 0.00 | 0.00 | 1.00 | 6.00 |
| TZEI 2540 | 0.00 | 0.00 | 0.00 | 0.00 | 0.00 | 1.00 | 6.00 |
| TZEI 2547 | 0.00 | 0.00 | 0.00 | 0.00 | 0.00 | 1.00 | 6.00 |
| TZEI 2551 | 0.00 | 0.00 | 0.00 | 0.00 | 0.00 | 1.00 | 6.00 |
| TZEI 2552 | 0.00 | 0.00 | 0.00 | 0.00 | 0.00 | 1.00 | 6.00 |
| TZEI 2558 | 0.00 | 0.00 | 0.00 | 0.00 | 0.00 | 1.00 | 6.00 |
| TZEI 2562 | 0.00 | 0.00 | 0.00 | 0.00 | 0.00 | 1.00 | 6.00 |
| TZEI 2563 | 0.00 | 0.00 | 0.00 | 0.00 | 0.00 | 1.00 | 6.00 |
| TZEI 2238 | 0.12 | 0.01 | 0.13 | 0.59 | 0.08 | 0.07 | admix |
| TZEI 2250 | 0.11 | 0.01 | 0.17 | 0.55 | 0.09 | 0.07 | admix |
| TZEI 2260 | 0.01 | 0.01 | 0.64 | 0.15 | 0.01 | 0.18 | admix |
| TZEI 2263 | 0.15 | 0.07 | 0.02 | 0.53 | 0.22 | 0.00 | admix |
| TZEI 2273 | 0.11 | 0.07 | 0.13 | 0.52 | 0.14 | 0.03 | admix |
| TZEI 2274 | 0.13 | 0.04 | 0.04 | 0.56 | 0.23 | 0.01 | admix |
| TZEI 2282 | 0.00 | 0.01 | 0.54 | 0.18 | 0.07 | 0.20 | admix |
| TZEI 2285 | 0.11 | 0.05 | 0.09 | 0.46 | 0.17 | 0.12 | admix |
| TZEI 2429 | 0.12 | 0.03 | 0.09 | 0.50 | 0.14 | 0.12 | admix |
| TZEI 2431 | 0.00 | 0.51 | 0.00 | 0.41 | 0.00 | 0.07 | admix |
| TZEI 2433 | 0.01 | 0.17 | 0.00 | 0.76 | 0.06 | 0.01 | admix |
| TZEI 2434 | 0.06 | 0.15 | 0.01 | 0.71 | 0.07 | 0.01 | admix |
| TZEI 2441 | 0.13 | 0.11 | 0.10 | 0.51 | 0.12 | 0.04 | admix |
| TZEI 2442 | 0.10 | 0.01 | 0.06 | 0.61 | 0.18 | 0.04 | admix |
| TZEI 2443 | 0.18 | 0.03 | 0.18 | 0.42 | 0.18 | 0.00 | admix |
| TZEI 2445 | 0.16 | 0.10 | 0.13 | 0.55 | 0.06 | 0.00 | admix |
| TZEI 2446 | 0.15 | 0.07 | 0.09 | 0.45 | 0.13 | 0.10 | admix |
| TZEI 2447 | 0.14 | 0.13 | 0.00 | 0.26 | 0.46 | 0.01 | admix |
| TZEI 2448 | 0.29 | 0.02 | 0.07 | 0.36 | 0.23 | 0.03 | admix |
| TZEI 2451 | 0.03 | 0.06 | 0.08 | 0.53 | 0.30 | 0.00 | admix |
| TZEI 2453 | 0.09 | 0.09 | 0.13 | 0.48 | 0.13 | 0.06 | admix |
| TZEI 2454 | 0.06 | 0.01 | 0.14 | 0.44 | 0.23 | 0.12 | admix |
| TZEI 2455 | 0.18 | 0.08 | 0.15 | 0.45 | 0.14 | 0.00 | admix |
| TZEI 2457 | 0.16 | 0.09 | 0.19 | 0.47 | 0.09 | 0.00 | admix |
| TZEI 2458 | 0.07 | 0.06 | 0.15 | 0.42 | 0.26 | 0.05 | admix |
| TZEI 2459 | 0.11 | 0.00 | 0.08 | 0.78 | 0.03 | 0.00 | admix |
| TZEI 2463 | 0.18 | 0.02 | 0.07 | 0.39 | 0.33 | 0.01 | admix |
| TZEI 2465 | 0.25 | 0.08 | 0.01 | 0.45 | 0.21 | 0.01 | admix |
| TZEI 2466 | 0.03 | 0.04 | 0.01 | 0.58 | 0.34 | 0.00 | admix |
| TZEI 2467 | 0.09 | 0.10 | 0.05 | 0.45 | 0.14 | 0.18 | admix |
| TZEI 2469 | 0.19 | 0.06 | 0.00 | 0.48 | 0.26 | 0.01 | admix |
| TZEI 2470 | 0.10 | 0.08 | 0.08 | 0.62 | 0.10 | 0.01 | admix |
| TZEI 2474 | 0.01 | 0.09 | 0.07 | 0.60 | 0.20 | 0.04 | admix |
| TZEI 2475 | 0.03 | 0.00 | 0.00 | 0.69 | 0.19 | 0.09 | admix |
| TZEI 2477 | 0.24 | 0.08 | 0.01 | 0.43 | 0.24 | 0.01 | admix |
| TZEI 2478 | 0.05 | 0.09 | 0.01 | 0.54 | 0.31 | 0.00 | admix |
| TZEI 2479 | 0.08 | 0.09 | 0.16 | 0.48 | 0.12 | 0.07 | admix |
| TZEI 2481 | 0.13 | 0.07 | 0.07 | 0.46 | 0.21 | 0.06 | admix |
| TZEI 2482 | 0.01 | 0.05 | 0.00 | 0.61 | 0.33 | 0.01 | admix |
| TZEI 2483 | 0.13 | 0.02 | 0.12 | 0.73 | 0.01 | 0.00 | admix |
| TZEI 2484 | 0.19 | 0.00 | 0.00 | 0.60 | 0.18 | 0.03 | admix |
| TZEI 2486 | 0.00 | 0.10 | 0.07 | 0.60 | 0.18 | 0.06 | admix |
| TZEI 2487 | 0.01 | 0.00 | 0.10 | 0.57 | 0.15 | 0.17 | admix |
| TZEI 2490 | 0.24 | 0.07 | 0.01 | 0.43 | 0.25 | 0.00 | admix |
| TZEI 2491 | 0.08 | 0.04 | 0.01 | 0.63 | 0.24 | 0.00 | admix |
| TZEI 2494 | 0.15 | 0.11 | 0.01 | 0.52 | 0.19 | 0.01 | admix |
| TZEI 2495 | 0.11 | 0.10 | 0.10 | 0.52 | 0.17 | 0.00 | admix |
| TZEI 2496 | 0.12 | 0.01 | 0.11 | 0.76 | 0.01 | 0.00 | admix |
| TZEI 2497 | 0.20 | 0.00 | 0.01 | 0.55 | 0.20 | 0.04 | admix |
| TZEI 2499 | 0.00 | 0.08 | 0.09 | 0.61 | 0.17 | 0.05 | admix |
| TZEI 2500 | 0.03 | 0.01 | 0.01 | 0.67 | 0.19 | 0.10 | admix |
| TZEI 2502 | 0.08 | 0.02 | 0.09 | 0.41 | 0.34 | 0.08 | admix |
| TZEI 2503 | 0.05 | 0.02 | 0.01 | 0.65 | 0.26 | 0.01 | admix |
| TZEI 2505 | 0.73 | 0.01 | 0.05 | 0.16 | 0.05 | 0.01 | admix |
| TZEI 2506 | 0.16 | 0.07 | 0.04 | 0.53 | 0.20 | 0.00 | admix |
| TZEI 2507 | 0.13 | 0.14 | 0.01 | 0.27 | 0.44 | 0.01 | admix |
| TZEI 2508 | 0.14 | 0.00 | 0.12 | 0.72 | 0.02 | 0.00 | admix |
| TZEI 2509 | 0.17 | 0.00 | 0.00 | 0.64 | 0.17 | 0.02 | admix |
| TZEI 2511 | 0.07 | 0.12 | 0.04 | 0.64 | 0.12 | 0.01 | admix |
| TZEI 2512 | 0.05 | 0.00 | 0.01 | 0.66 | 0.18 | 0.10 | admix |
| TZEI 2514 | 0.09 | 0.01 | 0.04 | 0.63 | 0.19 | 0.04 | admix |
| TZEI 2515 | 0.17 | 0.04 | 0.17 | 0.51 | 0.11 | 0.01 | admix |
| TZEI 2516 | 0.76 | 0.00 | 0.03 | 0.15 | 0.06 | 0.00 | admix |
| TZEI 2517 | 0.14 | 0.02 | 0.10 | 0.56 | 0.18 | 0.00 | admix |
| TZEI 2518 | 0.18 | 0.14 | 0.00 | 0.23 | 0.45 | 0.00 | admix |
| TZEI 2519 | 0.25 | 0.01 | 0.08 | 0.40 | 0.25 | 0.01 | admix |
| TZEI 2520 | 0.18 | 0.00 | 0.00 | 0.62 | 0.16 | 0.04 | admix |
| TZEI 2522 | 0.08 | 0.01 | 0.01 | 0.74 | 0.15 | 0.01 | admix |
| TZEI 2523 | 0.14 | 0.10 | 0.02 | 0.57 | 0.12 | 0.05 | admix |
| TZEI 2525 | 0.10 | 0.02 | 0.06 | 0.60 | 0.19 | 0.04 | admix |
| TZEI 2526 | 0.14 | 0.03 | 0.17 | 0.55 | 0.11 | 0.01 | admix |
| TZEI 2527 | 0.13 | 0.08 | 0.22 | 0.47 | 0.10 | 0.01 | admix |
| TZEI 2528 | 0.15 | 0.01 | 0.15 | 0.55 | 0.10 | 0.04 | admix |
| TZEI 2529 | 0.17 | 0.13 | 0.00 | 0.26 | 0.42 | 0.02 | admix |
| TZEI 2530 | 0.30 | 0.01 | 0.09 | 0.34 | 0.24 | 0.03 | admix |
| TZEI 2531 | 0.16 | 0.00 | 0.00 | 0.62 | 0.15 | 0.06 | admix |
| TZEI 2533 | 0.10 | 0.03 | 0.01 | 0.67 | 0.18 | 0.01 | admix |
| TZEI 2534 | 0.17 | 0.09 | 0.01 | 0.59 | 0.11 | 0.03 | admix |
| TZEI 2541 | 0.06 | 0.08 | 0.20 | 0.37 | 0.13 | 0.16 | admix |
| TZEI 2542 | 0.11 | 0.02 | 0.06 | 0.50 | 0.22 | 0.11 | admix |
| TZEI 2553 | 0.04 | 0.01 | 0.20 | 0.52 | 0.20 | 0.02 | admix |
| TZEI 2554 | 0.19 | 0.00 | 0.05 | 0.56 | 0.19 | 0.01 | admix |
| TZEI 2564 | 0.01 | 0.00 | 0.25 | 0.49 | 0.14 | 0.11 | admix |
| TZEI 2565 | 0.15 | 0.00 | 0.02 | 0.39 | 0.40 | 0.04 | admix |
| TZEI 2698 | 0.03 | 0.10 | 0.03 | 0.78 | 0.00 | 0.05 | admix |
| TZEI 2702 | 0.00 | 0.01 | 0.07 | 0.78 | 0.01 | 0.14 | admix |
| TZEI 2703 | 0.00 | 0.10 | 0.00 | 0.79 | 0.06 | 0.05 | admix |
| TZEI 2708 | 0.00 | 0.14 | 0.04 | 0.75 | 0.06 | 0.00 | admix |
| TZEI 2715 | 0.05 | 0.03 | 0.01 | 0.79 | 0.07 | 0.06 | admix |
| TZEI 2732 | 0.02 | 0.15 | 0.01 | 0.80 | 0.02 | 0.01 | admix |
| TZEI 2733 | 0.00 | 0.11 | 0.03 | 0.78 | 0.00 | 0.07 | admix |
| TZEI 2737 | 0.01 | 0.05 | 0.12 | 0.78 | 0.00 | 0.04 | admix |
| TZEI 2742 | 0.01 | 0.12 | 0.05 | 0.79 | 0.02 | 0.01 | admix |
| TZEI 2759 | 0.05 | 0.04 | 0.05 | 0.78 | 0.08 | 0.00 | admix |
| TZEI 2761 | 0.06 | 0.12 | 0.00 | 0.79 | 0.01 | 0.03 | admix |
| TZEI 2762 | 0.05 | 0.05 | 0.01 | 0.79 | 0.10 | 0.01 | admix |
| TZEI 2767 | 0.08 | 0.06 | 0.01 | 0.78 | 0.02 | 0.06 | admix |
| TZEI 2772 | 0.04 | 0.14 | 0.00 | 0.77 | 0.01 | 0.04 | admix |
| TZEI 2773 | 0.01 | 0.14 | 0.01 | 0.78 | 0.06 | 0.01 | admix |
| TZEI 2774 | 0.02 | 0.01 | 0.07 | 0.78 | 0.11 | 0.01 | admix |
| TZEI 2783 | 0.05 | 0.14 | 0.00 | 0.76 | 0.01 | 0.04 | admix |
| TZEI 2784 | 0.01 | 0.12 | 0.05 | 0.72 | 0.09 | 0.00 | admix |
| TZEI 2789 | 0.05 | 0.01 | 0.05 | 0.56 | 0.15 | 0.18 | admix |
| TZEI 2792 | 0.00 | 0.64 | 0.00 | 0.28 | 0.00 | 0.07 | admix |
| TZEI 2801 | 0.06 | 0.01 | 0.08 | 0.53 | 0.14 | 0.18 | admix |
| TZEI 2807 | 0.02 | 0.34 | 0.01 | 0.63 | 0.01 | 0.00 | admix |
| TZEI 2808 | 0.08 | 0.01 | 0.01 | 0.75 | 0.12 | 0.04 | admix |
| TZEI 2813 | 0.17 | 0.02 | 0.00 | 0.62 | 0.17 | 0.01 | admix |
| TZEI 2818 | 0.08 | 0.18 | 0.00 | 0.73 | 0.01 | 0.01 | admix |
| TZEI 2820 | 0.08 | 0.01 | 0.03 | 0.76 | 0.07 | 0.05 | admix |
| TZEI 2823 | 0.06 | 0.17 | 0.01 | 0.67 | 0.09 | 0.00 | admix |
| TZEI 2830 | 0.12 | 0.06 | 0.01 | 0.60 | 0.20 | 0.00 | admix |
| TZEI 2834 | 0.00 | 0.60 | 0.00 | 0.36 | 0.00 | 0.03 | admix |
| TZEI 2835 | 0.06 | 0.19 | 0.00 | 0.73 | 0.00 | 0.01 | admix |
| TZEI 2840 | 0.00 | 0.10 | 0.11 | 0.77 | 0.01 | 0.00 | admix |
| TZEI 2842 | 0.11 | 0.07 | 0.01 | 0.63 | 0.18 | 0.01 | admix |
| TZEI 2846 | 0.00 | 0.61 | 0.00 | 0.36 | 0.02 | 0.00 | admix |
| TZEI 2847 | 0.07 | 0.23 | 0.00 | 0.66 | 0.00 | 0.03 | admix |
| TZEI 2848 | 0.07 | 0.03 | 0.00 | 0.76 | 0.10 | 0.04 | admix |
| TZEI 2853 | 0.06 | 0.05 | 0.04 | 0.60 | 0.13 | 0.13 | admix |
| TZEI 2858 | 0.03 | 0.11 | 0.00 | 0.77 | 0.09 | 0.00 | admix |
| TZEI 2863 | 0.03 | 0.18 | 0.01 | 0.75 | 0.01 | 0.01 | admix |
| TZEI 2865 | 0.08 | 0.03 | 0.03 | 0.58 | 0.16 | 0.12 | admix |
| TZEI 2869 | 0.00 | 0.04 | 0.00 | 0.77 | 0.10 | 0.08 | admix |
| TZEI 2870 | 0.04 | 0.06 | 0.05 | 0.74 | 0.09 | 0.02 | admix |

Table S3: Gene flow based on population structure and pedigree information of the 376-elite white inbreds

| Source | Group 1 | Group 2 |
| --- | --- | --- |
| Group 1 | 0 |  |
| Group 2 | 7.999 | 0 |
| Group 3 | 2.225 | 4.597 |
|  | DTE STR-W Syn Pop C4 | TZEI 65 x ENT 11 |
| DTE STR-W Syn Pop C4 | 0 |  |
| TZEI 65 x ENT 11 | 44.468 | 0 |
| TZE-W Pop DT C5 STR C5 | 5.026 | 5.98 |

Table S4: Pedigree information of the 376 lines genotyped by 1904 SNP markers in this study

| Serial No. | Pedigree | Inbred lines | Serial No. | Pedigree | Inbred lines |
| --- | --- | --- | --- | --- | --- |
| 1 | (TZE-W Pop DT C5 STR C5 )S7 | TZEI.2695 | 26 | (TZE-W Pop DT C5 STR C5)S7 | TZEI.2722 |
| 2 | (TZE-W Pop DT C5 STR C5)S7 | TZEI.2696 | 27 | (TZE-W Pop DT C5 STR C5)S7 | TZEI.2724 |
| 3 | (TZE-W Pop DT C5 STR C5)S7 | TZEI.2697 | 28 | (TZE-W Pop DT C5 STR C5)S7 | TZEI.2725A |
| 4 | (TZE-W Pop DT C5 STR C5)S7 | TZEI.2698 | 29 | (TZE-W Pop DT C5 STR C5)S7 | TZEI.2725B |
| 5 | (TZE-W Pop DT C5 STR C5)S7 | TZEI.2699 | 30 | (TZE-W Pop DT C5 STR C5)S7 | TZEI.2726A |
| 6 | (TZE-W Pop DT C5 STR C5)S7 | TZEI.2700 | 31 | (TZE-W Pop DT C5 STR C5)S7 | TZEI.2726B |
| 7 | (TZE-W Pop DT C5 STR C5)S7 | TZEI.2701 | 32 | (TZE-W Pop DT C5 STR C5)S7 | TZEI.2727 |
| 8 | (TZE-W Pop DT C5 STR C5)S7 | TZEI.2702 | 33 | (TZE-W Pop DT C5 STR C5)S7 | TZEI.2728 |
| 9 | (TZE-W Pop DT C5 STR C5)S7 | TZEI.2703 | 34 | (TZE-W Pop DT C5 STR C5)S7 | TZEI.2729 |
| 10 | (TZE-W Pop DT C5 STR C5)S7 | TZEI.2704 | 35 | (TZE-W Pop DT C5 STR C5)S7 | TZEI.2730 |
| 11 | (TZE-W Pop DT C5 STR C5)S7 | TZEI.2705 | 36 | (TZE-W Pop DT C5 STR C5)S7 | TZEI.2731 |
| 12 | (TZE-W Pop DT C5 STR C5)S7 | TZEI.2706 | 37 | (TZE-W Pop DT C5 STR C5)S7 | TZEI.2732 |
| 13 | (TZE-W Pop DT C5 STR C5)S7 | TZEI.2707 | 38 | (TZE-W Pop DT C5 STR C5)S7 | TZEI.2733 |
| 14 | (TZE-W Pop DT C5 STR C5)S7 | TZEI.2708 | 39 | (TZE-W Pop DT C5 STR C5)S7 | TZEI.2734 |
| 15 | (TZE-W Pop DT C5 STR C5)S7 | TZEI.2710 | 40 | (TZE-W Pop DT C5 STR C5)S7 | TZEI.2735A |
| 16 | (TZE-W Pop DT C5 STR C5)S7 | TZEI.2711 | 41 | (TZE-W Pop DT C5 STR C5)S7 | TZEI.2735B |
| 17 | (TZE-W Pop DT C5 STR C5)S7 | TZEI.2712 | 42 | (TZE-W Pop DT C5 STR C5)S7 | TZEI.2736A |
| 18 | (TZE-W Pop DT C5 STR C5)S7 | TZEI.2713 | 43 | (TZE-W Pop DT C5 STR C5)S7 | TZEI.2736B |
| 19 | (TZE-W Pop DT C5 STR C5)S7 | TZEI.2714 | 44 | (TZE-W Pop DT C5 STR C5)S7 | TZEI.2737 |
| 20 | (TZE-W Pop DT C5 STR C5)S7 | TZEI.2715 | 45 | (TZE-W Pop DT C5 STR C5)S7 | TZEI.2738 |
| 21 | (TZE-W Pop DT C5 STR C5)S7 | TZEI.2716 | 46 | (TZE-W Pop DT C5 STR C5)S7 | TZEI.2739 |
| 22 | (TZE-W Pop DT C5 STR C5)S7 | TZEI.2717 | 47 | (TZE-W Pop DT C5 STR C5)S7 | TZEI.2740 |
| 23 | (TZE-W Pop DT C5 STR C5)S7 | TZEI.2718 | 48 | (TZE-W Pop DT C5 STR C5)S7 | TZEI.2741A |
| 24 | (TZE-W Pop DT C5 STR C5)S7 | TZEI.2719 | 49 | (TZE-W Pop DT C5 STR C5)S7 | TZEI.2741B |
| 25 | (TZE-W Pop DT C5 STR C5)S7 | TZEI.2720 | 50 | (TZE-W Pop DT C5 STR C5)S7 | TZEI.2742 |

| Serial No. | | Pedigree | | Inbred lines | | Serial No. | | Pedigree | | Inbred lines | |
| --- | --- | --- | --- | --- | --- | --- | --- | --- | --- | --- | --- |
| 51 | | (TZE-W Pop DT C5 STR C5)S7 | | TZEI.2743 | | 76 | | (TZE-W Pop DT C5 STR C5)S7 | | TZEI.2767 | |
| 52 | | (TZE-W Pop DT C5 STR C5)S7 | | TZEI.2744 | | 77 | | (TZE-W Pop DT C5 STR C5)S7 | | TZEI.2768 | |
| 53 | | (TZE-W Pop DT C5 STR C5)S7 | | TZEI.2745 | | 78 | | (TZE-W Pop DT C5 STR C5)S7 | | TZEI.2769 | |
| 54 | | (TZE-W Pop DT C5 STR C5)S7 | | TZEI.2746A | | 79 | | (TZE-W Pop DT C5 STR C5)S7 | | TZEI.2770 | |
| 55 | | (TZE-W Pop DT C5 STR C5)S7 | | TZEI.2746B | | 80 | | (TZE-W Pop DT C5 STR C5)S7 | | TZEI.2771 | |
| 56 | | (TZE-W Pop DT C5 STR C5)S7 | | TZEI.2747 | | 81 | | (TZE-W Pop DT C5 STR C5)S7 | | TZEI.2772 | |
| 57 | | (TZE-W Pop DT C5 STR C5)S7 | | TZEI.2748 | | 82 | | (TZE-W Pop DT C5 STR C5)S7 | | TZEI.2773 | |
| 58 | | (TZE-W Pop DT C5 STR C5)S7 | | TZEI.2749 | | 83 | | (TZE-W Pop DT C5 STR C5)S7 | | TZEI.2774 | |
| 59 | | (TZE-W Pop DT C5 STR C5)S7 | | TZEI.2750 | | 84 | | (TZE-W Pop DT C5 STR C5)S7 | | TZEI.2775 | |
| 60 | | (TZE-W Pop DT C5 STR C5)S7 | | TZEI.2751 | | 85 | | (TZE-W Pop DT C5 STR C5)S7 | | TZEI.2776 | |
| 61 | | (TZE-W Pop DT C5 STR C5)S7 | | TZEI.2752 | | 86 | | (TZE-W Pop DT C5 STR C5)S7 | | TZEI.2777 | |
| 62 | | (TZE-W Pop DT C5 STR C5)S7 | | TZEI.2753 | | 87 | | (TZE-W Pop DT C5 STR C5)S7 | | TZEI.2778 | |
| 63 | | (TZE-W Pop DT C5 STR C5)S7 | | TZEI.2754 | | 88 | | (TZE-W Pop DT C5 STR C5)S7 | | TZEI.2779 | |
| 64 | | (TZE-W Pop DT C5 STR C5)S7 | | TZEI.2755 | | 89 | | (TZE-W Pop DT C5 STR C5)S7 | | TZEI.2780 | |
| 65 | | (TZE-W Pop DT C5 STR C5)S7 | | TZEI.2756 | | 90 | | (TZE-W Pop DT C5 STR C5)S7 | | TZEI.2781 | |
| 66 | | (TZE-W Pop DT C5 STR C5)S7 | | TZEI.2757 | | 91 | | (TZE-W Pop DT C5 STR C5)S7 | | TZEI.2782 | |
| 67 | | (TZE-W Pop DT C5 STR C5)S7 | | TZEI.2758 | | 92 | | (TZE-W Pop DT C5 STR C5)S7 | | TZEI.2783 | |
| 68 | | (TZE-W Pop DT C5 STR C5)S7 | | TZEI.2759 | | 93 | | (TZE-W Pop DT C5 STR C5)S7 | | TZEI.2784 | |
| 69 | | (TZE-W Pop DT C5 STR C5)S7 | | TZEI.2760 | | 94 | | (TZE-W Pop DT C5 STR C5)S7 | | TZEI.2785 | |
| 70 | | (TZE-W Pop DT C5 STR C5)S7 | | TZEI.2761 | | 95 | | (TZE-W Pop DT C5 STR C5)S7 | | TZEI.2786 | |
| 71 | | (TZE-W Pop DT C5 STR C5)S7 | | TZEI.2762 | | 96 | | (TZE-W Pop DT C5 STR C5)S7 | | TZEI.2787 | |
| 72 | | (TZE-W Pop DT C5 STR C5)S7 | | TZEI.2763 | | 97 | | (TZE-W Pop DT C5 STR C5)S7 | | TZEI.2788 | |
| 73 | | (TZE-W Pop DT C5 STR C5)S7 | | TZEI.2764 | | 98 | | (TZE-W Pop DT C5 STR C5)S7 | | TZEI.2789 | |
| 74 | | (TZE-W Pop DT C5 STR C5)S7 | | TZEI.2765 | | 99 | | (TZE-W Pop DT C5 STR C5)S7 | | TZEI.2790 | |
| 75 | | (TZE-W Pop DT C5 STR C5)S7 | | TZEI.2766 | | 100 | | (TZE-W Pop DT C5 STR C5)S7 | | TZEI.2791 | |
| Serial No. | Pedigree | | Inbred lines | | Serial No. | | Pedigree | | Inbred lines | |  |
| 101 | (TZE-W Pop DT C5 STR C5)S7 | | TZEI.2792 | | 126 | | (TZE-W Pop DT C5 STR C5)S7 | | TZEI.2817 | |  |
| 102 | (TZE-W Pop DT C5 STR C5)S7 | | TZEI.2793 | | 127 | | (TZE-W Pop DT C5 STR C5)S7 | | TZEI.2818 | |  |
| 103 | (TZE-W Pop DT C5 STR C5)S7 | | TZEI.2794 | | 128 | | (TZE-W Pop DT C5 STR C5)S7 | | TZEI.2819 | |  |
| 104 | (TZE-W Pop DT C5 STR C5)S7 | | TZEI.2795 | | 129 | | (TZE-W Pop DT C5 STR C5)S7 | | TZEI.2820 | |  |
| 105 | (TZE-W Pop DT C5 STR C5)S7 | | TZEI.2796 | | 130 | | (TZE-W Pop DT C5 STR C5)S7 | | TZEI.2821 | |  |
| 106 | (TZE-W Pop DT C5 STR C5)S7 | | TZEI.2797 | | 131 | | (TZE-W Pop DT C5 STR C5)S7 | | TZEI.2822 | |  |
| 107 | (TZE-W Pop DT C5 STR C5)S7 | | TZEI.2798 | | 132 | | (TZE-W Pop DT C5 STR C5)S7 | | TZEI.2823 | |  |
| 108 | (TZE-W Pop DT C5 STR C5)S7 | | TZEI.2799 | | 133 | | (TZE-W Pop DT C5 STR C5)S7 | | TZEI.2824 | |  |
| 109 | (TZE-W Pop DT C5 STR C5)S7 | | TZEI.2800 | | 134 | | (TZE-W Pop DT C5 STR C5)S7 | | TZEI.2830 | |  |
| 110 | (TZE-W Pop DT C5 STR C5)S7 | | TZEI.2801 | | 135 | | (TZE-W Pop DT C5 STR C5)S7 | | TZEI.2831 | |  |
| 111 | (TZE-W Pop DT C5 STR C5)S7 | | TZEI.2802 | | 136 | | (TZE-W Pop DT C5 STR C5)S7 | | TZEI.2832 | |  |
| 112 | (TZE-W Pop DT C5 STR C5)S7 | | TZEI.2803 | | 137 | | (TZE-W Pop DT C5 STR C5)S7 | | TZEI.2833 | |  |
| 113 | (TZE-W Pop DT C5 STR C5)S7 | | TZEI.2804 | | 138 | | (TZE-W Pop DT C5 STR C5)S7 | | TZEI.2834 | |  |
| 114 | (TZE-W Pop DT C5 STR C5)S7 | | TZEI.2805 | | 139 | | (TZE-W Pop DT C5 STR C5)S7 | | TZEI.2835 | |  |
| 115 | (TZE-W Pop DT C5 STR C5)S7 | | TZEI.2806 | | 140 | | (TZE-W Pop DT C5 STR C5)S7 | | TZEI.2836 | |  |
| 116 | (TZE-W Pop DT C5 STR C5)S7 | | TZEI.2807 | | 141 | | (TZE-W Pop DT C5 STR C5)S7 | | TZEI.2837 | |  |
| 117 | (TZE-W Pop DT C5 STR C5)S7 | | TZEI.2808 | | 142 | | (TZE-W Pop DT C5 STR C5)S7 | | TZEI.2838 | |  |
| 118 | (TZE-W Pop DT C5 STR C5)S7 | | TZEI.2809 | | 143 | | (TZE-W Pop DT C5 STR C5)S7 | | TZEI.2839 | |  |
| 119 | (TZE-W Pop DT C5 STR C5)S7 | | TZEI.2810 | | 144 | | (TZE-W Pop DT C5 STR C5)S7 | | TZEI.2840 | |  |
| 120 | (TZE-W Pop DT C5 STR C5)S7 | | TZEI.2811 | | 145 | | (TZE-W Pop DT C5 STR C5)S7 | | TZEI.2841 | |  |
| 121 | (TZE-W Pop DT C5 STR C5)S7 | | TZEI.2812 | | 146 | | (TZE-W Pop DT C5 STR C5)S7 | | TZEI.2842 | |  |
| 122 | (TZE-W Pop DT C5 STR C5)S7 | | TZEI.2813 | | 147 | | (TZE-W Pop DT C5 STR C5)S7 | | TZEI.2843 | |  |
| 123 | (TZE-W Pop DT C5 STR C5)S7 | | TZEI.2814 | | 148 | | (TZE-W Pop DT C5 STR C5)S7 | | TZEI.2844 | |  |
| 124 | (TZE-W Pop DT C5 STR C5)S7 | | TZEI.2815 | | 149 | | (TZE-W Pop DT C5 STR C5)S7 | | TZEI.2845 | |  |
| 125 | (TZE-W Pop DT C5 STR C5)S7 | | TZEI.2816 | | 150 | | (TZE-W Pop DT C5 STR C5)S7 | | TZEI.2846 | |  |

| Pedigree | Inbred lines | Serial No. | Pedigree | Inbred lines |
| --- | --- | --- | --- | --- |
| (TZE-W Pop DT C5 STR C5)S7 | TZEI.2847 | 176 | (TZE-W Pop DT C5 STR C5)S7 | TZEI.2872 |
| (TZE-W Pop DT C5 STR C5)S7 | TZEI.2848 | 177 | (TZE-W Pop DT C5 STR C5)S7 | TZEI.2873 |
| (TZE-W Pop DT C5 STR C5)S7 | TZEI.2849 | 178 | (TZE-W Pop DT C5 STR C5)S7 | TZEI.2874 |
| (TZE-W Pop DT C5 STR C5)S7 | TZEI.2850 | 179 | (TZE-W Pop DT C5 STR C5)S7 | TZEI.2875 |
| (TZE-W Pop DT C5 STR C5)S7 | TZEI.2851 | 180 | (DTE STR-W Syn Pop C4)S7 | TZEI.2429 |
| (TZE-W Pop DT C5 STR C5)S7 | TZEI.2852 | 181 | (DTE STR-W Syn Pop C4)S7 | TZEI.2430 |
| (TZE-W Pop DT C5 STR C5)S7 | TZEI.2853 | 182 | (DTE STR-W Syn Pop C4)S7 | TZEI.2431 |
| (TZE-W Pop DT C5 STR C5)S7 | TZEI.2854 | 183 | (DTE STR-W Syn Pop C4)S7 | TZEI.2432 |
| (TZE-W Pop DT C5 STR C5)S7 | TZEI.2855 | 184 | (DTE STR-W Syn Pop C4)S7 | TZEI.2433 |
| (TZE-W Pop DT C5 STR C5)S7 | TZEI.2856 | 185 | (DTE STR-W Syn Pop C4)S7 | TZEI.2434 |
| (TZE-W Pop DT C5 STR C5)S7 | TZEI.2857 | 186 | (DTE STR-W Syn Pop C4)S7 | TZEI.2437 |
| (TZE-W Pop DT C5 STR C5)S7 | TZEI.2858 | 187 | (DTE STR-W Syn Pop C4)S7 | TZEI.2438 |
| (TZE-W Pop DT C5 STR C5)S7 | TZEI.2859 | 188 | (DTE STR-W Syn Pop C4)S7 | TZEI.2439 |
| (TZE-W Pop DT C5 STR C5)S7 | TZEI.2860 | 189 | (DTE STR-W Syn Pop C4)S7 | TZEI.2440 |
| (TZE-W Pop DT C5 STR C5)S7 | TZEI.2861 | 190 | (DTE STR-W Syn Pop C4)S7 | TZEI.2441 |
| (TZE-W Pop DT C5 STR C5)S7 | TZEI.2862 | 191 | (DTE STR-W Syn Pop C4)S7 | TZEI.2442 |
| (TZE-W Pop DT C5 STR C5)S7 | TZEI.2863 | 192 | (DTE STR-W Syn Pop C4)S7 | TZEI.2443 |
| (TZE-W Pop DT C5 STR C5)S7 | TZEI.2864 | 193 | (DTE STR-W Syn Pop C4)S7 | TZEI.2444 |
| (TZE-W Pop DT C5 STR C5)S7 | TZEI.2865 | 194 | (DTE STR-W Syn Pop C4)S7 | TZEI.2445 |
| (TZE-W Pop DT C5 STR C5)S7 | TZEI.2866 | 195 | (DTE STR-W Syn Pop C4)S7 | TZEI.2446 |
| (TZE-W Pop DT C5 STR C5)S7 | TZEI.2867 | 196 | (DTE STR-W Syn Pop C4)S7 | TZEI.2447 |
| (TZE-W Pop DT C5 STR C5)S7 | TZEI.2868 | 197 | (DTE STR-W Syn Pop C4)S7 | TZEI.2448 |
| (TZE-W Pop DT C5 STR C5)S7 | TZEI.2869 | 198 | (DTE STR-W Syn Pop C4)S7 | TZEI.2449 |
| (TZE-W Pop DT C5 STR C5)S7 | TZEI.2870 | 199 | (DTE STR-W Syn Pop C4)S7 | TZEI.2450 |
| (TZE-W Pop DT C5 STR C5)S7 | TZEI.2871 | 200 | (DTE STR-W Syn Pop C4)S7 | TZEI.2451 |

| Pedigree | Inbred lines | Serial No. | Pedigree | Inbred lines |
| --- | --- | --- | --- | --- |
| (DTE STR-W Syn Pop C4)S7 | TZEI.2452 | 226 | (DTE STR-W Syn Pop C4)S7 | TZEI.2477 |
| (DTE STR-W Syn Pop C4)S7 | TZEI.2453 | 227 | (DTE STR-W Syn Pop C4)S7 | TZEI.2478 |
| (DTE STR-W Syn Pop C4)S7 | TZEI.2454 | 228 | (DTE STR-W Syn Pop C4)S7 | TZEI.2479 |
| (DTE STR-W Syn Pop C4)S7 | TZEI.2455 | 229 | (DTE STR-W Syn Pop C4)S7 | TZEI.2480 |
| (DTE STR-W Syn Pop C4)S7 | TZEI.2456 | 230 | (DTE STR-W Syn Pop C4)S7 | TZEI.2481 |
| (DTE STR-W Syn Pop C4)S7 | TZEI.2457 | 231 | (DTE STR-W Syn Pop C4)S7 | TZEI.2482 |
| (DTE STR-W Syn Pop C4)S7 | TZEI.2458 | 232 | (DTE STR-W Syn Pop C4)S7 | TZEI.2483 |
| (DTE STR-W Syn Pop C4)S7 | TZEI.2459 | 233 | (DTE STR-W Syn Pop C4)S7 | TZEI.2484 |
| (DTE STR-W Syn Pop C4)S7 | TZEI.2460 | 234 | (DTE STR-W Syn Pop C4)S7 | TZEI.2485 |
| (DTE STR-W Syn Pop C4)S7 | TZEI.2461 | 235 | (DTE STR-W Syn Pop C4)S7 | TZEI.2486 |
| (DTE STR-W Syn Pop C4)S7 | TZEI.2462 | 236 | (DTE STR-W Syn Pop C4)S7 | TZEI.2487 |
| (DTE STR-W Syn Pop C4)S7 | TZEI.2463 | 237 | (DTE STR-W Syn Pop C4)S7 | TZEI.2489 |
| (DTE STR-W Syn Pop C4)S7 | TZEI.2464 | 238 | (DTE STR-W Syn Pop C4)S7 | TZEI.2490 |
| (DTE STR-W Syn Pop C4)S7 | TZEI.2465 | 239 | (DTE STR-W Syn Pop C4)S7 | TZEI.2491 |
| (DTE STR-W Syn Pop C4)S7 | TZEI.2466 | 240 | (DTE STR-W Syn Pop C4)S7 | TZEI.2492 |
| (DTE STR-W Syn Pop C4)S7 | TZEI.2467 | 241 | (DTE STR-W Syn Pop C4)S7 | TZEI.2493 |
| (DTE STR-W Syn Pop C4)S7 | TZEI.2468 | 242 | (DTE STR-W Syn Pop C4)S7 | TZEI.2494 |
| (DTE STR-W Syn Pop C4)S7 | TZEI.2469 | 243 | (DTE STR-W Syn Pop C4)S7 | TZEI.2495 |
| (DTE STR-W Syn Pop C4)S7 | TZEI.2470 | 244 | (DTE STR-W Syn Pop C4)S7 | TZEI.2496 |
| (DTE STR-W Syn Pop C4)S7 | TZEI.2471 | 245 | (DTE STR-W Syn Pop C4)S7 | TZEI.2497 |
| (DTE STR-W Syn Pop C4)S7 | TZEI.2472 | 246 | (DTE STR-W Syn Pop C4)S7 | TZEI.2498 |
| (DTE STR-W Syn Pop C4)S7 | TZEI.2473 | 247 | (DTE STR-W Syn Pop C4)S7 | TZEI.2499 |
| (DTE STR-W Syn Pop C4)S7 | TZEI.2474 | 248 | (DTE STR-W Syn Pop C4)S7 | TZEI.2500 |
| (DTE STR-W Syn Pop C4)S7 | TZEI.2475 | 249 | (DTE STR-W Syn Pop C4)S7 | TZEI.2501 |
| (DTE STR-W Syn Pop C4)S7 | TZEI.2476 | 250 | (DTE STR-W Syn Pop C4)S7 | TZEI.2502 |

| Serial No. | Pedigree | Inbred lines | Serial No. | Pedigree | Inbred lines |
| --- | --- | --- | --- | --- | --- |
| 251 | (DTE STR-W Syn Pop C4)S7 | TZEI.2503 | 276 | (DTE STR-W Syn Pop C4)S7 | TZEI.2528 |
| 252 | (DTE STR-W Syn Pop C4)S7 | TZEI.2504 | 277 | (DTE STR-W Syn Pop C4)S7 | TZEI.2529 |
| 253 | (DTE STR-W Syn Pop C4)S7 | TZEI.2505 | 278 | (DTE STR-W Syn Pop C4)S7 | TZEI.2530 |
| 254 | (DTE STR-W Syn Pop C4)S7 | TZEI.2506 | 279 | (DTE STR-W Syn Pop C4)S7 | TZEI.2531 |
| 255 | (DTE STR-W Syn Pop C4)S7 | TZEI.2507 | 280 | (DTE STR-W Syn Pop C4)S7 | TZEI.2532 |
| 256 | (DTE STR-W Syn Pop C4)S7 | TZEI.2508 | 281 | (DTE STR-W Syn Pop C4)S7 | TZEI.2533 |
| 257 | (DTE STR-W Syn Pop C4)S7 | TZEI.2509 | 282 | (DTE STR-W Syn Pop C4)S7 | TZEI.2534 |
| 258 | (DTE STR-W Syn Pop C4)S7 | TZEI.2510 | 283 | (DTE STR-W Syn Pop C4)S7 | TZEI.2535 |
| 259 | (DTE STR-W Syn Pop C4)S7 | TZEI.2511 | 284 | (DTE STR-W Syn Pop C4)S7 | TZEI.2536 |
| 260 | (DTE STR-W Syn Pop C4)S7 | TZEI.2512 | 285 | (DTE STR-W Syn Pop C4)S7 | TZEI.2537 |
| 261 | (DTE STR-W Syn Pop C4)S7 | TZEI.2513 | 286 | (DTE STR-W Syn Pop C4)S7 | TZEI.2538 |
| 262 | (DTE STR-W Syn Pop C4)S7 | TZEI.2514 | 287 | (DTE STR-W Syn Pop C4)S7 | TZEI.2539 |
| 263 | (DTE STR-W Syn Pop C4)S7 | TZEI.2515 | 288 | (DTE STR-W Syn Pop C4)S7 | TZEI.2540 |
| 264 | (DTE STR-W Syn Pop C4)S7 | TZEI.2516 | 289 | (DTE STR-W Syn Pop C4)S7 | TZEI.2541 |
| 265 | (DTE STR-W Syn Pop C4)S7 | TZEI.2517 | 290 | (DTE STR-W Syn Pop C4)S7 | TZEI.2542 |
| 266 | (DTE STR-W Syn Pop C4)S7 | TZEI.2518 | 291 | (DTE STR-W Syn Pop C4)S7 | TZEI.2543 |
| 267 | (DTE STR-W Syn Pop C4)S7 | TZEI.2519 | 292 | (DTE STR-W Syn Pop C4)S7 | TZEI.2544 |
| 268 | (DTE STR-W Syn Pop C4)S7 | TZEI.2520 | 293 | (DTE STR-W Syn Pop C4)S7 | TZEI.2545 |
| 269 | (DTE STR-W Syn Pop C4)S7 | TZEI.2521 | 294 | (DTE STR-W Syn Pop C4)S7 | TZEI.2546 |
| 270 | (DTE STR-W Syn Pop C4)S7 | TZEI.2522 | 295 | (DTE STR-W Syn Pop C4)S7 | TZEI.2547 |
| 271 | (DTE STR-W Syn Pop C4)S7 | TZEI.2523 | 296 | (DTE STR-W Syn Pop C4)S7 | TZEI.2548 |
| 272 | (DTE STR-W Syn Pop C4)S7 | TZEI.2524 | 297 | (DTE STR-W Syn Pop C4)S7 | TZEI.2549 |
| 273 | (DTE STR-W Syn Pop C4)S7 | TZEI.2525 | 298 | (DTE STR-W Syn Pop C4)S7 | TZEI.2550 |
| 274 | (DTE STR-W Syn Pop C4)S7 | TZEI.2526 | 299 | (DTE STR-W Syn Pop C4)S7 | TZEI.2551 |
| 275 | (DTE STR-W Syn Pop C4)S7 | TZEI.2527 | 300 | (DTE STR-W Syn Pop C4)S7 | TZEI.2552 |
| Serial No. | Pedigree | Inbred lines | Serial No. | Pedigree | Inbred lines |
| 301 | (DTE STR-W Syn Pop C4)S7 | TZEI.2553 | 326 | (TZEI 65 x ENT 11)S7 | TZEI.2239 |
| 302 | (DTE STR-W Syn Pop C4)S7 | TZEI.2554 | 327 | (TZEI 65 x ENT 11)S7 | TZEI.2240 |
| 303 | (DTE STR-W Syn Pop C4)S7 | TZEI.2555 | 328 | (TZEI 65 x ENT 11)S7 | TZEI.2241 |
| 304 | (DTE STR-W Syn Pop C4)S7 | TZEI.2556 | 329 | (TZEI 65 x ENT 11)S7 | TZEI.2242 |
| 305 | (DTE STR-W Syn Pop C4)S7 | TZEI.2557A | 330 | (TZEI 65 x ENT 11)S7 | TZEI.2243 |
| 306 | (DTE STR-W Syn Pop C4)S7 | TZEI.2557B | 331 | (TZEI 65 x ENT 11)S7 | TZEI.2244 |
| 307 | (DTE STR-W Syn Pop C4)S7 | TZEI.2558 | 332 | (TZEI 65 x ENT 11)S7 | TZEI.2245 |
| 308 | (DTE STR-W Syn Pop C4)S7 | TZEI.2559 | 333 | (TZEI 65 x ENT 11)S7 | TZEI.2246 |
| 309 | (DTE STR-W Syn Pop C4)S7 | TZEI.2560 | 334 | (TZEI 65 x ENT 11)S7 | TZEI.2247 |
| 310 | (DTE STR-W Syn Pop C4)S7 | TZEI.2561 | 335 | (TZEI 65 x ENT 11)S7 | TZEI.2248 |
| 311 | (DTE STR-W Syn Pop C4)S7 | TZEI.2562 | 336 | (TZEI 65 x ENT 11)S7 | TZEI.2249 |
| 312 | (DTE STR-W Syn Pop C4)S7 | TZEI.2563 | 337 | (TZEI 65 x ENT 11)S7 | TZEI.2250 |
| 313 | (DTE STR-W Syn Pop C4)S7 | TZEI.2564 | 338 | (TZEI 65 x ENT 11)S7 | TZEI.2252 |
| 314 | (DTE STR-W Syn Pop C4)S7 | TZEI.2565 | 339 | (TZEI 65 x ENT 11)S7 | TZEI.2253 |
| 315 | (DTE STR-W Syn Pop C4)S7 | TZEI.2566 | 340 | (TZEI 65 x ENT 11)S7 | TZEI.2254 |
| 316 | (DTE STR-W Syn Pop C4)S7 | TZEI.2567 | 341 | (TZEI 65 x ENT 11)S7 | TZEI.2255 |
| 317 | (DTE STR-W Syn Pop C4)S7 | TZEI.2568 | 342 | (TZEI 65 x ENT 11)S7 | TZEI.2256 |
| 318 | (TZEI 65 x ENT 11)S7 | TZEI.2231 | 343 | (TZEI 65 x ENT 11)S7 | TZEI.2257 |
| 319 | (TZEI 65 x ENT 11)S7 | TZEI.2232 | 344 | (TZEI 65 x ENT 11)S7 | TZEI.2258 |
| 320 | (TZEI 65 x ENT 11)S7 | TZEI.2233 | 345 | (TZEI 65 x ENT 11)S7 | TZEI.2259 |
| 321 | (TZEI 65 x ENT 11)S7 | TZEI.2234 | 346 | (TZEI 65 x ENT 11)S7 | TZEI.2260 |
| 322 | (TZEI 65 x ENT 11)S7 | TZEI.2235 | 347 | (TZEI 65 x ENT 11)S7 | TZEI.2261 |
| 323 | (TZEI 65 x ENT 11)S7 | TZEI.2236 | 348 | (TZEI 65 x ENT 11)S7 | TZEI.2262 |
| 324 | (TZEI 65 x ENT 11)S7 | TZEI.2237 | 349 | (TZEI 65 x ENT 11)S7 | TZEI.2263 |
| 325 | (TZEI 65 x ENT 11)S7 | TZEI.2238 | 350 | (TZEI 65 x ENT 11)S7 | TZEI.2264 |

| Pedigree | Inbred lines | Serial No. | Pedigree | Inbred lines |
| --- | --- | --- | --- | --- |
| (TZEI 65 x ENT 11)S7 | TZEI.2265 | 364 | (TZEI 65 x ENT 11)S7 | TZEI.2278 |
| (TZEI 65 x ENT 11)S7 | TZEI.2266 | 365 | (TZEI 65 x ENT 11)S7 | TZEI.2279 |
| (TZEI 65 x ENT 11)S7 | TZEI.2267 | 366 | (TZEI 65 x ENT 11)S7 | TZEI.2280 |
| (TZEI 65 x ENT 11)S7 | TZEI.2268 | 367 | (TZEI 65 x ENT 11)S7 | TZEI.2281 |
| (TZEI 65 x ENT 11)S7 | TZEI.2269 | 368 | (TZEI 65 x ENT 11)S7 | TZEI.2282 |
| (TZEI 65 x ENT 11)S7 | TZEI.2270 | 369 | (TZEI 65 x ENT 11)S7 | TZEI.2283 |
| (TZEI 65 x ENT 11)S7 | TZEI.2271 | 370 | (TZEI 65 x ENT 11)S7 | TZEI.2284 |
| (TZEI 65 x ENT 11)S7 | TZEI.2272 | 371 | (TZEI 65 x ENT 11)S7 | TZEI.2285 |
| (TZEI 65 x ENT 11)S7 | TZEI.2273 | 372 | (TZEI 65 x ENT 11)S7 | TZEI.2286 |
| (TZEI 65 x ENT 11)S7 | TZEI.2274 | 373 | (TZEI 65 x ENT 11)S7 | TZEI.2287 |
| (TZEI 65 x ENT 11)S7 | TZEI.2275 | 374 | (TZEI 65 x ENT 11)S7 | TZEI.2288 |
| (TZEI 65 x ENT 11)S7 | TZEI.2276 | 375 | (TZEI 65 x ENT 11)S7 | TZEI.2289 |
| (TZEI 65 x ENT 11)S7 | TZEI.2277 | 376 | (TZEI 65 x ENT 11)S7 | TZEI.2290 |

Figure S1: Pedigree-based PCA scatter plot of the 376 early maturing maize inbred lines along PC1 and PC2 using 1904 SNP markers


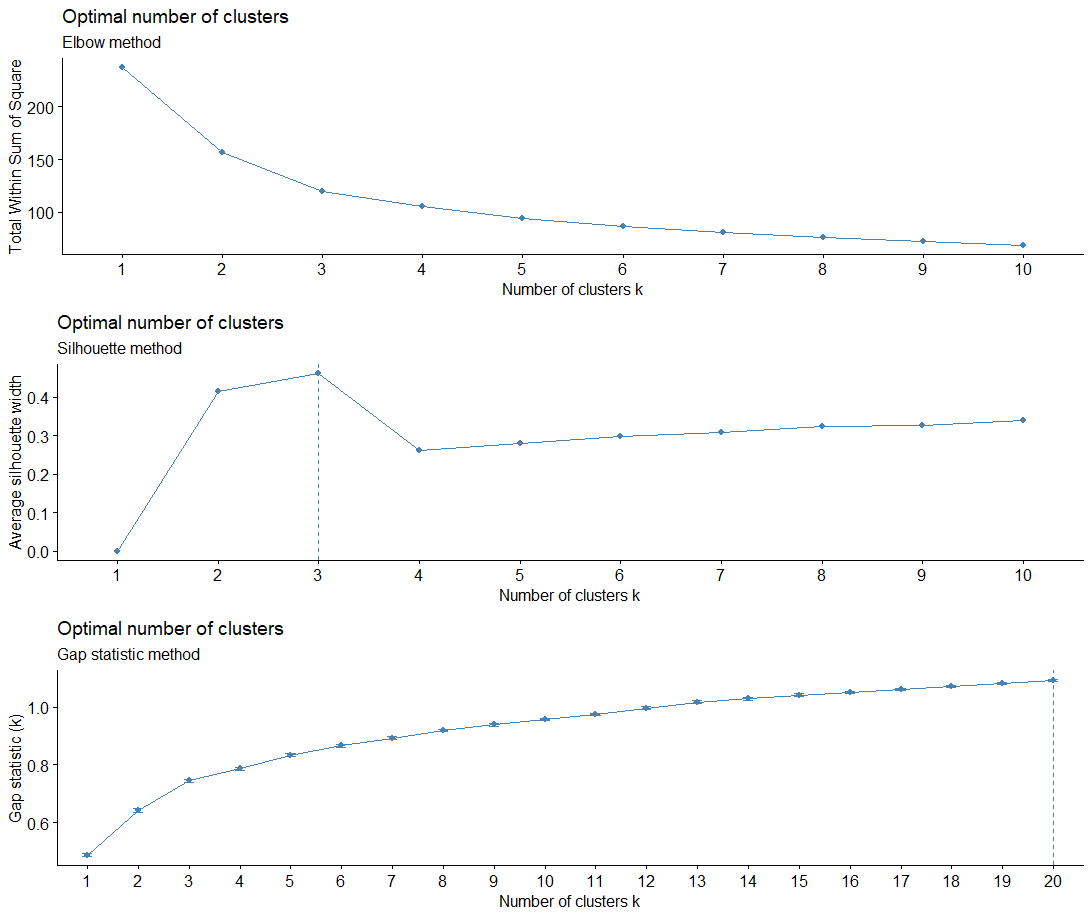


Figure S2: Optimum population structure based on elbow method (upper), Silhouette method (middle), and Gap statistics (lower)
