# Supplementary figures and images for: Optimizing breeding strategies for early-maturing white maize through genetic diversity and population structure
Source: PLoS One. 2025 Feb 24;20(2):e0316793. doi: 10.1371/journal.pone.0316793 (PMC11849899; doi:10.1371/journal.pone.0316793)

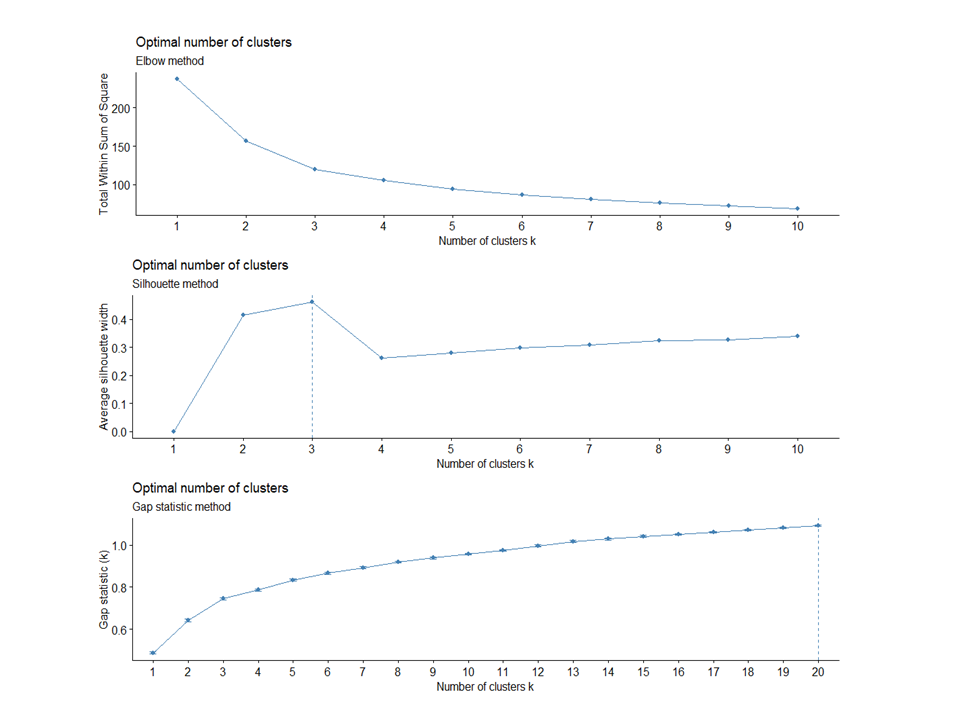

Supplement: S1 Fig — (TIF) [file pone.0316793.s002.tif]

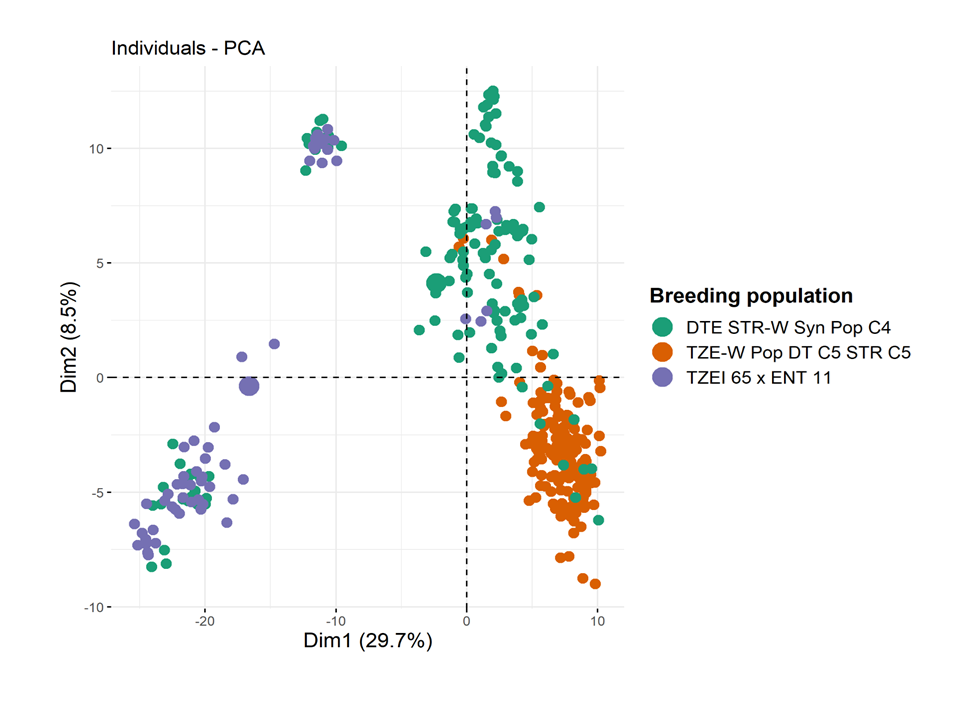

Supplement: S2 Fig — (TIF) [file pone.0316793.s003.tif]
