# Supplementary material for: Optimizing breeding strategies for early-maturing white maize through genetic diversity and population structure
Source: PLoS One. 2025 Feb 24;20(2):e0316793. doi: 10.1371/journal.pone.0316793 (PMC11849899; doi:10.1371/journal.pone.0316793)
Supplement: Attachment — Submitted filename: PONE-D-24-43431 Reviewed.pdf [file pone.0316793.s004.pdf]

PLOS ONE

Assessment of genetic diversity, population structure, and clustering methods for heterotic grouping in early-maturing white maize (Zea mays L.) inbred lines

--Manuscript Draft--

**Manuscript Number:**	PONE-D-24-43431
**Article Type:**	Research Article
**Full Title:**	Assessment of genetic diversity, population structure, and clustering methods for heterotic grouping in early-maturing white maize (Zea mays L.) inbred lines
**Short Title:**	Genetic Structure and Heterotic groupings of Early-Maturing White Maize inbreds
**Corresponding Author:**	Idris Ishola Adejumobi, Ph.D.<br>International Institute of Tropical Agriculture<br>Ibadan, Oyo NIGERIA
**Keywords:**	Keywords; Early maturing maize, genetic variability, heterotic groups, single nucleotide polymorphic markers
**Abstract:**	Maize production and productivity in Sub-Saharan Africa face a myriad of constraints. Assessment of genetic diversity in elite inbreds provides the opportunity to identify lines that possess desirable genes and to explore the genetic relatedness of maize lines for heterotic breeding by selecting parents with complementary genetic backgrounds. The objectives of this study were to assess the level of genetic diversity, and population structure, and identify appropriate clustering methods for assigning maize inbreds into heterotic groups. Three hundred and seventy-six elite inbreds extracted from three source populations were genotyped using Diversity Array Technology (DArTtag) mid-density genotyping. Results revealed 3305 SNPs out of which 1904 were retained after quality control. The average marker polymorphic information content (PIC) was 0.39, observed heterozygosity was 0.02, gene diversity was 0.37, and minor allele frequency was 0.29. The optimum sub-population was three defined by an admixture-based model and principal component analysis. The average genetic distance was 0.303 varying from 0.03 (TZEI 2772 and TZEI 2761) to 0.372 (TZEI 2273 and TZEI 2832). Of the four genetic distances and seven clustering methods, combination of IBS distance and the average linkage clustering method was identified as the best heterotic grouping technique with three groups identified. The pedigree-based phylogenetic tree revealed the possibility of shared alleles among the source populations. The F-statistics based on the underlying population structure revealed 10% variation among sub-populations and 90% variation within sub-populations with a moderate level of genetic differentiation (0.10). The elite inbred lines showed a high degree of genetic diversity, which could be beneficial for breeding new, early-maturing white hybrids.
**Order of Authors:**	Hellen Mawia Mukiti, MSc
	Baffour Badu-Apraku, Ph.D
	Idris Ishola Adejumobi, Ph.D
	John Derera, Ph.D
	Ayodeji Abe, Ph.D
**Additional Information:**	
**Question**	**Response**
**Financial Disclosure**<br><br>Enter a financial disclosure statement that describes the sources of funding for the work included in this submission. Review the submission guidelines for detailed requirements. View published research articles from *PLOS ONE* for specific	Yes

Powered by Editorial Manager® and ProduXion Manager® from Aries Systems Corporation

examples.<br><br>This statement is required for submission and **will appear in the published article** if the submission is accepted. Please make sure it is accurate.<br><br>**Funded studies**<br>Enter a statement with the following details:<br>• Initials of the authors who received each award<br>• Grant numbers awarded to each author<br>• The full name of each funder<br>• URL of each funder website<br>• Did the sponsors or funders play any role in the study design, data collection and analysis, decision to publish, or preparation of the manuscript?<br><br>Did you receive funding for this work?	
Please add funding details.<br>as follow-up to "**Financial Disclosure**<br><br>Enter a financial disclosure statement that describes the sources of funding for the work included in this submission. Review the submission guidelines for detailed requirements. View published research articles from *PLOS ONE* for specific examples.<br><br>This statement is required for submission and **will appear in the published article** if the submission is accepted. Please make sure it is accurate.<br><br>**Funded studies**<br>Enter a statement with the following details:<br>• Initials of the authors who received each award<br>• Grant numbers awarded to each author<br>• The full name of each funder<br>• URL of each funder website<br>• Did the sponsors or funders play any role in the study design, data collection and analysis, decision to publish, or preparation of the manuscript?	Bill and Melinda Gates Foundation through the Accelerated Genetic Gains and Stress Tolerant Maize for Africa Projects [OPP1134248].

Powered by Editorial Manager® and ProduXion Manager® from Aries Systems Corporation

Did you receive funding for this work?"	
Please select the country of your main research funder (please select carefully as in some cases this is used in fee calculation).<br>as follow-up to "**Financial Disclosure**<br><br>Enter a financial disclosure statement that describes the sources of funding for the work included in this submission. Review the submission guidelines for detailed requirements. View published research articles from *PLOS ONE* for specific examples.<br><br>This statement is required for submission and **will appear in the published article** if the submission is accepted. Please make sure it is accurate.<br><br>**Funded studies**<br>Enter a statement with the following details:<br>• Initials of the authors who received each award<br>• Grant numbers awarded to each author<br>• The full name of each funder<br>• URL of each funder website<br>• Did the sponsors or funders play any role in the study design, data collection and analysis, decision to publish, or preparation of the manuscript?<br><br>Did you receive funding for this work?"	UNITED STATES - US
**Competing Interests**<br><br>Use the instructions below to enter a competing interest statement for this submission. On behalf of all authors, disclose any competing interests that could be perceived to bias this work—acknowledging all financial support and any other relevant financial or non-financial competing interests.	I have read the journal's policy, and the authors of this manuscript do not have any competing interests.

This statement is required for submission and **will appear in the published article** if the submission is accepted. Please make sure it is accurate and that any funding sources listed in your Funding Information later in the submission form are also declared in your Financial Disclosure statement.<br><br>View published research articles from *PLOS ONE* for specific examples.<br><br>**NO authors have competing interests**<br><br>Enter: *The authors have declared that no competing interests exist.*<br><br>**Authors with competing interests**<br><br>Enter competing interest details beginning with this statement:<br><br>*I have read the journal's policy and the authors of this manuscript have the following competing interests: [insert competing interests here]*<br><br>* typeset	
**Ethics Statement**<br><br>Enter an ethics statement for this submission. This statement is required if the study involved:<br><br>• Human participants<br>• Human specimens or tissue<br>• Vertebrate animals or cephalopods<br>• Vertebrate embryos or tissues<br>• Field research<br><br>Write "N/A" if the submission does not require an ethics statement.<br><br>General guidance is provided below. Consult the submission guidelines for detailed instructions. **Make sure that all information entered here is included in the Methods section of the manuscript.**	N/A

**Format for specific study types**<br><br>**Human Subject Research (involving human participants and/or tissue)**<br>• Give the name of the institutional review board or ethics committee that approved the study<br>• Include the approval number and/or a statement indicating approval of this research<br>• Indicate the form of consent obtained (written/oral) or the reason that consent was not obtained (e.g. the data were analyzed anonymously)<br><br>**Animal Research (involving vertebrate animals, embryos or tissues)**<br>• Provide the name of the Institutional Animal Care and Use Committee (IACUC) or other relevant ethics board that reviewed the study protocol, and indicate whether they approved this research or granted a formal waiver of ethical approval<br>• Include an approval number if one was obtained<br>• If the study involved *non-human primates*, add *additional details* about animal welfare and steps taken to ameliorate suffering<br>• If anesthesia, euthanasia, or any kind of animal sacrifice is part of the study, include briefly which substances and/or methods were applied<br><br>**Field Research**<br><br>Include the following details if this study involves the collection of plant, animal, or other materials from a natural setting:<br>• Field permit number<br>• Name of the institution or relevant body that granted permission	
**Data Availability**<br><br>Authors are required to make all data underlying the findings described fully available, without restriction, and from the time of publication. PLOS allows rare exceptions to address legal and ethical concerns. See the PLOS Data Policy and FAQ for detailed information.	Yes - all data are fully available without restriction

A Data Availability Statement describing where the data can be found is required at submission. Your answers to this question constitute the Data Availability Statement and **will be published in the article**, if accepted.<br><br>**Important:** Stating 'data available on request from the author' is not sufficient. If your data are only available upon request, select 'No' for the first question and explain your exceptional situation in the text box.<br><br>Do the authors confirm that all data underlying the findings described in their manuscript are fully available without restriction?	
**Describe where the data may be found in full sentences. If you are copying our sample text, replace any instances of XXX with the appropriate details.**<br><br>• If the data are **held or will be held in a public repository**, include URLs, accession numbers or DOIs. If this information will only be available after acceptance, indicate this by ticking the box below. For example: *All XXX files are available from the XXX database (accession number(s) XXX, XXX.*).<br>• If the data are all contained **within the manuscript and/or Supporting Information files**, enter the following: *All relevant data are within the manuscript and its Supporting Information files.*<br>• If neither of these applies but you are able to provide **details of access elsewhere**, with or without limitations, please do so. For example:<br><br>*Data cannot be shared publicly because of [XXX]. Data are available from the XXX Institutional Data Access / Ethics Committee (contact via XXX) for researchers who meet the criteria for access to confidential data.*<br><br>*The data underlying the results presented in the study are available*	The raw dataset for this study can be obtained from the IITA CKAN repository at https://data.iita.org/dataset and https://ebs.excellenceinbreeding.org/

from (include the name of the third party and contact information or URL).<br>• This text is appropriate if the data are owned by a third party and authors do not have permission to share the data.<br><br>* typeset	
Additional data availability information:	

Manuscript

Click here to access/download;Manuscript;Hellen_Mukiti_PLOS ONE_submission.docx

Assessment of genetic diversity, population structure, and clustering methods for heterotic grouping in early-maturing white maize (*Zea mays* L.) inbred lines

Hellen Mawia Mukiti[1, 2], Baffour Badu-Apraku[1*], Ayodeji Abe[3], Idris Ishola Adejumobi[1*], John Derera[1]

[1] International Institute of Tropical Agriculture (IITA) Ibadan, Nigeria; bbaduapraku@gmail.com; j.derera@cgiar.org; i.adejumobi@cgiar.org

[2] Pan African University Life and Earth Sciences Institute (including Health and Agriculture), University of Ibadan, Ibadan 200284, Oyo State, Nigeria; hellenmmukiti@gmail.com[3]

[3] Department of Crop and Horticultural Sciences, University of Ibadan, Ibadan 200284, Oyo State, Nigeria: a.abe@ui.edu.ng

* Correspondence: bbaduapraku@gmail.com; i.adejumobi@cgiar.org

ABSTRACT

Maize production and productivity in Sub-Saharan Africa face a myriad of constraints. Assessment of genetic diversity in elite inbreds provides the opportunity to identify lines that possess desirable genes and to explore the genetic relatedness of maize lines for heterotic breeding by selecting parents with complementary genetic backgrounds. The objectives of this study were to assess the level of genetic diversity, and population structure, and identify appropriate clustering methods for assigning maize inbreds into heterotic groups. Three hundred and seventy-six elite inbreds extracted from three source populations were genotyped using Diversity Array Technology (DArTtag) mid-density genotyping. Results revealed 3305 SNPs out of which 1904 were retained after quality control. The average marker polymorphic information content (PIC) was 0.39, observed heterozygosity was 0.02, gene diversity was 0.37, and minor allele frequency was 0.29. The optimum sub-population was three defined by an admixture-based model and principal component analysis. The average genetic distance was 0.303 varying from 0.03 (TZEI 2772 and TZEI 2761) to 0.372 (TZEI 2273 and TZEI 2832). Of the four genetic distances and seven clustering methods, combination of IBS distance and the average linkage clustering method was identified as the best heterotic grouping technique with three groups identified. The pedigree-based phylogenetic tree revealed the possibility of shared alleles among the source populations. The F-statistics based on the underlying population structure revealed 10% variation among sub-populations and 90% variation within sub-populations with a moderate level of genetic differentiation (0.10). The elite inbred lines showed a high degree of genetic diversity, which could be beneficial for breeding new, early-maturing white hybrids.

Keywords; Early-maturing maize, genetic variability, heterotic groups, single nucleotide polymorphic markers

1. INTRODUCTION

Maize (*Zea mays* L.) is globally cultivated for several purposes including food, feed, and industrial needs (Erenstein et al., 2022). In sub-Saharan Africa (SSA), it is a primary food source and exhibits the greatest capacity for crop production in the savannah of the sub-region. Maize accounts for 40% of the African cereal production with utilization extending from direct consumption as food by the populace to processed products, and feeds for animals (Ranum et al., 2014). Maize consists of about 72% carbohydrate, 10% protein, and 4% fat, with an energy density of 365 Kcal/100g (Ranum et al., 2014). Approximately 60% of the populace in SSA depend on agriculture as their primary source of sustenance and income (Okou et al., 2022). However, due to the low productivity of maize constantly observed on farmers' fields, its potential to enhance food security is far from being met at the household and national levels. This is attributable to several constraints, which include drought, heat, low soil nitrogen, *Striga* parasitism, fall armyworm infestation, and several diseases (Baffour Badu-Apraku & Fakorede, 2017). Boosting maize production against these stress conditions is crucial to ensuring food security and livelihoods in SSA (Iqbal et al., 2001).

Genetic diversity is the raw material for any crop improvement program. Knowledge of the level of genetic diversity in germplasm is essential as it enables efficient allocation of resources to effectively meet breeding goals. The use of genetic variability in maize has been employed to select genetically diverse parents for hybrid production. Maize, being a species that experiences out-crossing, possesses a genome that is intricate and exhibits a substantial level of genetic diversity (Hake & Ross-Ibarra, 2015). These characteristics are beneficial to breeders as it allows them to attain a considerable increase in hybrid vigour, resulting in the development of maize varieties with high productivity. Precise data on the genetic diversity of maize lines in a breeding program could facilitate the identification and classification of inbreds into heterotic groups. This would allow the development of productive hybrids from contrasting heterotic groups without the need to test every possible combination among prospective parents (Moll et al., 1965). In assessing genetic diversity in maize germplasm, several methods including morphological, biochemical, and molecular markers have been employed. Of these methods, molecular markers provide more accurate and reliable results as it is independent of environmental influence (Akinwale et al., 2014). Utilising marker-based genetic distance (GD) estimates could help to avoid making hybrid crosses between closely related inbreds, especially when the heterotic groups of these lines are not clearly defined. Predictive analysis could be used to eliminate crosses with unsatisfactory performance and reduce the cost and the time involved in the identification of desired hybrids (Melchinger & Gumber, 1998). Over the decades, PCR-based markers, such as random amplified polymorphic DNA (RAPD), amplified fragment length polymorphism (AFLP), simple sequence repeats (SSR), single nucleotide polymorphism (SNP) markers have demonstrated their efficacy as robust techniques for analysing genetic variation and characterizing population structure in maize lines (Amiteye, 2021). However, SNP markers have gained high popularity in various genetic studies of the modern breeding era owing to their advantages over other marker types (Zhu et al., 2018).

In the last four decades, maize breeders at the International Institute of Tropical Agriculture (IITA; https://www.iita.org/) and International Maize and Wheat Improvement Center (CIMMYT; https://www.cimmyt.org/) in collaboration with the National Agricultural Research System (NARS) of several maize-growing countries have developed diverse range of germplasms. These germplasms comprised elite inbreds, populations, synthetics, and open-pollinated varieties and more recently hybrids (Baffour Badu-Apraku & Fakorede, 2017). Numerous public and private sector organizations use IITA and CIMMYT maize germplasm for a variety of purposes, including the development of open-pollinated varieties, production of hybrid seeds, breeding based on pedigree information, population development for mapping quantitative trait loci (QTL), production of doubled haploids,and the incorporation of traits using transgenic methods (Semagn et al., 2012). Improving food security in SSA depends on the development and deployment of superior maize genotypes preferably hybrids that combine tolerance to

the stress factors characterizing SSA with high-yielding capability, to replace the genetically weak varieties cultivated by many farmers. Hybrid production is of major interest owing to their superiority in grain yield from heterosis (Li et al., 2021). "Heterosis", a phenomenon elucidating the superiority of a hybrid over the mid-parent, better parent, or a reference genotype performance is only possible if there are distinct heterotic groups. Therefore, developing hybrid maize genotypes to boost maize production and productivity in SSA requires identifying parental inbreds from complementary genetic backgrounds and showing adequate levels of genetic diversity. Source populations with a high level of genetic variability could facilitate the extraction of such valuable inbreds (Semagn et al., 2012).

In defining heterotic groups, adequate measures need to be taken to identify the appropriate genetic distance matrix and linkage method to summarize the best dissimilarity matrices into distinct genetic groups or clusters (Padilla et al., 2007). Hierarchical clustering is the most widely used approach in the analysis of crop diversity and heterotic classification (Mohammadi & Prasanna, 2003). Popularly used genetic distance matrices include Identical-by-State, Gower distance, Euclidean distance (Roger), and Jaccard distance and for linkage methods includes complete linkage, single linkage, average linkage (UPGMA), centroid, ward.D2, McQuitty, and Median (Padilla et al., 2007, Mohammadi & Prasanna, 2003). Each of these approaches has some distinctive features and may generate different results. It therefore becomes imperative to identify the most appropriate combination of distance matrix and linkage method for the assignment of the inbred lines into heterotic groups. The objectives of this study were to assess the level of genetic diversity and population structure of 376 early white maize inbred lines, and to identify the best distance matrix and clustering linkage methods for assigning the inbreds into distinct heterotic groups.

2. MATERIALS AND METHODS

2.1 Genetic Materials Used for the Study

Three hundred and seventy-six elite S_7 early white multiple stress tolerant inbred lines were used for this study (Table S1). The inbred lines were newly developed from three source populations namely: DTE STR-W Syn Pop C4, TZE-W Pop DT C5 STR C5, and TZEI 65 x ENT 11 (Baffour Badu-Apraku & Fakorede, 2017) have described the details of the breeding approach used to develop the three-source populations. Briefly, DTE STR-W Syn Pop C4 was developed from the broad-based population DTE STR-W Syn Pop following four cycles of S_1 family recurrent selection. TZE-W Pop DT C5 STR C5 was developed from the broad-based population TZE-W Pop DT STR following five cycles of S_1 family recurrent selection. The biparental population TZEI 65 x ENT 11 was developed from the parental inbreds from IITA (TZEI 65) and CIMMYT (ENT 11). The development of the inbreds from these three populations began with the generation of S_1 lines. These lines were advanced through S_2 to S_4 stages of inbreeding by selfing. After each cycle of selfing, the lines were evaluated under artificial *Striga* infestation and induced moisture stress. At S_4, 250 – 300 selected lines were crossed to a broad-based tester to estimate their general combining ability effects and based on the testcrosses performance, 90 – 100 S_4 lines were advanced through S_5 to S_7 stage of inbreeding where homozygosity of the lines is expected to be 99%.

2.2 Leaf sample collection and genotyping

Seeds of the S_7 inbreds were planted in the field and leaf samples were collected at three weeks after planting. The samples were kept under -80 degrees Celsius conditions for 72 hours and lyophilized using a Labconco Freezone 2.5 L System lyophilizer (Marshall Scientific, USA). Leaf discs from three-week-old seedlings of each inbred were punched and placed in labelled propylene tubes. These tubes were then transferred to a deep-well plate and shipped to Intertek Laboratory, Australia, for mid-density DArTtag genotyping. The genotyping involved a series of steps, which combined molecular biology techniques with

bioinformatics analysis. To begin with, DNA was isolated from the leaf samples, purified, and digested with specific restriction enzymes that cut it at predetermined target sequences. These target sequences were taken through adapter ligation during which adapters containing uniqueoligonucleotide sequences were ligated to the ends of the restriction fragments. Following the adapter ligation was the representation by complexity reduction through a proprietary amplification technique that selectively enriched specific genomic regions containing the pre-selected SNP markers. The PCR amplification followed to generate millions of copies of the target sequence used for hybridization and single nucleotide polymorphism (SNP) analysis. This allowed the detection of variations at the targeted SNP sites. The hybridized probes were taken for imaging and data analysis on the DArT platform to identify the presence or absence of specific polymorphisms at each SNP marker locus for each sample. After this, data quality control and genotyping calls were done(Kilian et al., 2012).

2.3 Marker quality control

Multiple sequences were generated by the DArTtag platform using proprietary analytical pipelines. The sequence results received from the DArT platform had 3,305 SNP markers. From this, quality control was performed with SNP QC implemented in R using minor allele frequency (MAF) (0.05), call rate (≥ 90), missing rate (0.2), genotype quality (GQ = 20), maximum and minimum allele = 2, and no indels. The markers were reduced to 1904 SNP SNPs after the filtering process and were used for downstream analyses.

2.4 Assessment of genetic diversity, population structure, and clustering methods from SNP marker data

The final HapMap with 1904 high-quality SNP data was first converted to variant call format (VCF). Genetic diversity indices such as expected and observed heterozygosities (EH and OH), minor allele frequency (MAF), and polymorphic information content (PIC) are crucial tools for assessing the genetic variation within and among populations were estimated from the VCF file in PLINK 2.0 [17]. These parameters provide quantitative measures of the richness and evenness of alleles in the study population. The HapMap file was also converted to a minor allele frequency format from where the gene content file was generated in R (R core team, 2017). To reveal the genetic stratification in the elite inbred population, the gene content file containing the 1904 DArT markers was imported into the Bayesian Markov chain Monte Carlo (MCMC) software STRUCTURE V2.3.4 (Gao et al., 2007). Structure simulations were carried out using a burn-in period of 20,000 iterations and an MCMC set at 20,000 with no prior information on the origin of individuals. For the appropriate K-value, the Evanno transformation method was used which exhibited a low cross-validation error compared to other K values (Evanno et al., 2005). The results obtained from STRUCTURE were uploaded into Harvester to determine the optimum K-value. A membership probability level ≥80 was used to assign the inbreds to sub-populations, while inbreds with a probability level <80 were designated as admixture. Additionally, principal component analysis (PCA) was used to visualize the pattern of the genetic structure and the relationship among the 376 inbred source populations using the FactorMineR package (Lê et al., 2008) in R. To identify the best distance matrix and linkage methods for assigning the early white inbreds into heterotic groups, Four different genetic distance matrices were generated from the gene content file namely identity-by-state (IBS), Gower distance, Euclidian distance, and Jaccard distance based on equation 1 to 4, respectively.

$$\mathrm{IBS}(i,j) = \frac{\text{number of alleles shared between individuals i and j}}{\text{total number of alleles compared}}$$ ………………….equation 1

$$d(i,j) = 1 - \frac{(\Sigma\_\{k=1\}\^\{p\}\ S\_k(i,j))}{p}$$ ………………………………………….equation 2

Where; d(i, j) is the Gower distance between objects i and j; p is the total number of variables; S_k(i, j) is the similarity coefficient between objects i and j for variable k.

$$d(p, q) = sqrt((p1 - q1)^2 + (p2 - q2)^2 + \ldots + (pn - qn)^2) \ldots\ldots\ldots\ldots 3$$

Where; d(p, q) is the Euclidean distance between points p and q; p1, p2, ..., pn are the coordinates of point p; q1, q2, ..., qn are the coordinates of point q.

$$\text{Jaccard}\ (A, B) = \frac{|A \cap B|}{|A \cup B|} \ldots\ldots\ldots 4$$

Where; ∣A ∩ B∣ is the cardinality of the intersection of sets A and B (i.e., the number of elements common to both sets); ∣A ∪ B∣ is the cardinality of the union of sets A and B (the total number of elements in both sets, including duplicates).

These matrices were combined with seven types of linkage methods namely Complete linkage, Single linkage, Average linkage (UPGMA), Centroid, Ward minimum variance (ward.D2), McQuitty, and Median in the dendextend package (Tal & Vardy, 2015) in R for estimating the cophenetic correlation coefficient (CCC) between the distances and linkage methods pair. The distance and linkage method with the highest CCC were used to assign the inbreds into heterotic groups in the Analysis of Phylogenetic and Evolution (APE) package (Paradis & Schliep, 2019). The final dendrogram was constructed using the ggtree package (Yu et al., 2017) in R. Analysis of molecular variance (AMOVA) and genetic differentiation (F_{ST}) was done using GenAlEx 6.5 (Peakall & Smouse, 2012) to partition the variation between and within populations and pedigree of inbreds.

3. RESULTS

3.1 Summary of Genetic Diversity Indices

The diversity indices of the 376 inbred lines based on 1,904 SNP markers revealed that 57.6% have PIC greater than the average PIC of 0.39. The SNP markers on chromosome 9 has the highest PIC (0.41). The mean heterozygosity expected/gene diversity (EH) averaged 0.37 and ranged from 0.07 to 0.50. Markers on chromosome 9 also had the highest level of observed heterozygosity (0.40), while markers on chromosome 1 had the lowest (0.35). Observed heterozygosity (OH) averaged 0.02 with a range of 0.00 to 0.81. The MAF averaged 0.29, and ranged from 0.05 to 0.50. The MAF was highest in markers on chromosome 9 and lowest in markers on chromosome 1 and 10 (Table 1). The distribution of markers across the 10 chromosomes of the maize genome revealed that chromosome 10 had the least number of SNP markers (143), while chromosome 5 had the highest number (254) (Figure 1).

Table 1: Diversity indices of the 376 early maturing maize inbred lines based on 1,904 SNP markers

Chromosome	OH	EH	MAF	PIC
CHR1	0.02	0.35	0.27	0.38
CHR2	0.02	0.37	0.30	0.38
CHR3	0.02	0.37	0.28	0.39
CHR4	0.03	0.38	0.29	0.40
CHR5	0.02	0.37	0.28	0.39
CHR6	0.03	0.37	0.30	0.38

CHR7	0.02	0.38	0.30	0.39
CHR8	0.02	0.38	0.28	0.39
CHR9	0.03	0.40	0.31	0.41
CHR10	0.02	0.37	0.27	0.39
Min	0.00	0.07	0.05	0.00
Average	0.02	0.37	0.29	0.39
Max	0.81	0.50	0.50	0.50

EH; expected heterozygosity, OH; observed heterozygosity, MAF; minor allele frequency, PIC; polymorphic information content.

3.2 Population structure of the 376 elite early white inbred lines assessed with 1904 SNP markers

From the STRUCTURE results, the value of LnP(D) increased continuously from K = 1 to 10; with an inflection point at 2 and 6 (Fig. 2a and b). Based on the admixture model the 376 maize inbreds were grouped into two and six sub-populations, respectively (Fig. 2c and d). The Introduction of an 80% membership probability threshold resulted in a 9.6% (36 inbreds) level of admixture at K=2 and a 33% (125 inbreds) level of admixture at K=6 (Table S2 and S3). Of the inbreds designated as admixture from K=2 and K=6, 11 inbreds were common to both K values. The admixture levels at K =2 comprised 17 inbreds from TZEI 65 x ENT 11 and 19 inbreds from DTE STR-W Syn Pop C4. At K= 6 the admixture comprised 8 inbreds from TZEI 65 x ENT 11, 76 inbreds from DTE STR-W Syn Pop C4, and 41 inbreds from TZE-W Pop DT C5 STR C5. The visualization of the underlying population structure using PCA however, revealed three sub-populations. In addition, the pattern of the genetic stratification from PCA showed that sub-populations 1 had greater genetic closeness with sub-population 2 than with sub-population 3. Furthermore, sub-population 2 was stratified into two subgroups as observed in the PC1 and 2-plot view (Fig. 3).

3.3 Defining the best clustering method and heterotic groups of 376 early white inbred lines

The CCC of the genetic distance matrices and the hierarchical cluster (HC) clustering linkage methods presented in Table 2 showed that the IBS distance combined with the average linkage (UPGMA) method had the highest CCC (0.97). The clustering was therefore constructed with the UPGMA method using the IBS matrix. The IBS genetic dissimilarity/distance (GD) displayed a range of estimates from 0.002 between TZEI 2772 and TZEI 2761 to 0.372 between TZEI 2273 and TZEI 2832 with an average of 0.303. The HC assigned the 376-elite white inbreds to three major heterotic groups (Fig. 4). One hundred and eighty elite inbreds were assigned to heterotic group 1 (red) with an average GD of 0.302. This group had the largest number of cluster members. In this heterotic group, the lowest GD was between the inbred pair TZEI 2772 and TZEI 2761 (0.002), while the maximum GD (0.371) was between the inbred pair TZEI 2832 and TZEI 2273. Heterotic group 2 (green) comprised 137 inbreds with an average GD of 0.302. The lowest GD for this heterotic group was between the inbred pair TZEI 2477 and TZEI 2490 (0.012), while the highest GD (0.369) was between the inbred pair TZEI 2273 and TZEI 2748. Heterotic group 3 (blue) had the least number of cluster members (59 inbreds) with an average GD of 0.301. The lowest GD (0.013) was between the inbred pair TZEI 2259 and TZEI 2548, while the highest GD (0.366) was between the inbred pair TZEI 2271 and TZEI 2729 (Table 3). Considering a 35% dissimilarity among inbreds from opposing heterotic groups as a benchmark, 661 hybrid combinations were identified. These hybrids comprised 130 hybrid combinations between heterotic groups 1 and 2, 396 hybrid combinations between heterotic groups 1 and 3, and 135 hybrid combinations between heterotic groups 2 and 3 (Fig. 5).

Cophenetic correlation coefficients among the clustering linkage methods and genetic distance matrices [illegible]ployed to determine the best heterotic grouping methods for the 376 early-maturing white inbred lines

	Hierarchical Clustering methods						
**Genetic distance Matrix**	Average	Centroid	Complete	McQuitty	Median	Single	ward.D2
Euclidean	0.95	0.21	0.93	0.95	0.20	0.91	0.53
IBS	**0.97**	0.93	0.93	0.94	0.90	0.77	0.88
Jaccard	0.94	0.50	0.92	0.94	0.39	0.90	0.58
Gower	0.94	0.57	0.92	0.94	0.42	0.91	0.63

Table 3: Genetics distance from 1904 DArT-derived SNP markers characterizing the three heterotic groups defined for the 376 early maturing white inbred lines

	HG_1	HG_2	HG_3
Minimum	0.002 (TZEI 2772 and TZEI 2761)	0.012 (TZEI 2477 and TZEI 2490)	0.013 (TZEI 2259 and TZEI 2548)
Maximum	0.371 (TZEI 2832 and TZEI 2273)	0.369 (TZEI 2273 and TZEI 2748)	0.366 (TZEI 2271 and TZEI 2729)
Average	0.302	0.302	0.301

HG_1, heterotic group one; HG_2, heterotic group two; HG_3, heterotic group three

The PCA plot of the 376-elite inbreds based on pedigree information revealed the presence of common alleles among the source populations DTE STR-W Syn Pop C4, TZEI 65 x ENT 11, andTZE-W Pop DT C5 STR C5 (Fig. S1). Heterotic group 1 comprised 47.6% of all the inbred lines studied with 94.4 % derived from population TZE-W Pop DT C5 STR C5 and 5.6% derived from population DTE STR-W Syn Pop C4. Heterotic group 2 constituted 36.7 % of all the inbred lines studied and comprised inbred lines derived from all the three source populations. Inbred lines derived from populations DTE STR-W Syn Pop C4, TZE-W Pop DT C5STR C5 and TZEI 65 × ENT 11 constituted 79.0%, 5.8% and 15.2% of heterotic group 2. Inbred lines derived from TZEI 65 × ENT 11 made up 66.1% of the inbred lines derived from heterotic group 3, while the remaining inbreds were derived from DTE STR-W Syn Pop C4 (33.9%). The three heterotic groups shared inbred lines derived from DTE STR-W Syn Pop C4. The pedigree-based phylogenetic tree assigned 172 of 180 inbreds extracted from TZE-Pop DT C5 STR C5 into heterotic group 1, 109 of 138 inbreds extracted from DTE STR-W Syn Pop C4 into heterotic group 2, and 39 of 59 inbreds extracted from TZEI 65 x ENT 11 into heterotic group 3 (Fig. 6).

3.3 Analysis of Molecular Variance and Genetic Differentiation

The AMOVA results (Table 4 and 5) based on the heterotic group and pedigree information captured the spread of genetic variation between and within the heterotic groups and pedigrees (Table 6). Based on the heterotic structure, the results revealed a genetic variation of 10% among heterotic groups and 90% within heterotic groups, with a moderate level of genetic differentiation (0.099). Among pedigrees, genetic variation was 7%, while within-pedigrees variation was 93%, with a moderate level of genetic differentiation (0.073). Heterotic groups 1 and 2 had the lowest level of differentiation (0.059) and the highest gene flow (7.999), while the differentiation was highest (0.183) between heterotic groups 3 and 1 with the lowest gene flow

(2.225). Based on the source populations, DTE STR-W Syn Pop C4 and TZEI 65 x ENT 11 had the least pairwise differentiation (0.011) with the highest level of gene flow (44.468), while DTE STR-W Syn Pop C4 and TZE-W Pop DT C5 STR C5 had the highest differentiation (0.090) with the lowest gene flow (5.026).

Table 4: Analysis of molecular variance and genetic differentiation among 376 early-maturingmaize inbred lines assessed using 1904 SNP markers

**Source**	**DF**	**SS**	**MS**	**Est. Var.**	**%**	**Fst**	**P**
Among HG	2	9556.319	4778.159	38.403	10%	0.099	0.001
Within HG	373	130269.094	349.247	349.247	90%		
Total	375	139825.412		387.650	100%		
Source	**DF**	**SS**	**MS**	**Est. Var.**	**%**	**Fst**	**P**
Among Pedigree	2	7218.986	3609.493	28.188	7%	0.073	0.001
Within Pedigree	373	132606.426	355.513	355.513	93%		
Total	375	139825.412		383.701	100%		

DF: degrees of freedom; HG; Heterotic group; SS: sum of squares; MS: mean squares; Est.Var: estimated variance; %: percentage variance; Fst: Pairwise differentiation; P: significance level

Table 5: Pairwise population differentiation based on population structure and pedigreeinformation of the 376-elite white inbreds

Source	Group 1	Group 2	Group 3
Heterotic group 1	0.000	0.001	0.001
Heterotic group 2	0.059	0.000	0.001
Heterotic group 3	0.183	0.098	0.000
	DTE STR-W Syn Pop C4	TZEI 65 x ENT 11	TZE-W Pop DT C5 STR C5
DTE STR-W Syn Pop C4	0.000	0.001	0.001
TZEI 65 x ENT 11	0.011	0.000	0.001
TZE-W Pop DT C5 STR C5	0.090	0.077	0.000

4. DISCUSSION

In this study, 1904 SNP markers were used to assess the level of genetic diversity, population structure and the appropriate clustering method for assigning 376 elite early-maturing maize inbreds into distinct heterotic groups. The mean marker PIC showed that the markers were appropriate for assessing genetic diversity of the early elite inbreds used for the study. Polymorphic information content explains the usefulness of a genetic marker in linkage and diversity studies as it reveals the ability of markers to effectively differentiate and discriminate among genotypes. The average marker PIC obtained in this study

was comparable to those reported in tropical maize germplasm by (Adu et al., 2019; Badu-Apraku, 2021; Gedil et al., 2023) and higher than those reported by (Wu et al., 2015; Zhang et al., 2016). The average marker MAF suggested the presence of rare alleles in substantial proportions in the maize inbred population. Adu et al. (2019) reported similar findings. The expected heterozygosity indicated the probability of individuals possessing different alleles at a given SNP locus. The high estimates of expected heterozygosity as observed in this study suggested greater genetic diversity within the population for exploitation in the IITA maize-breeding program. The observed heterozygosity on the other hand indicated the actual proportion of individuals heterozygous at a SNP locus and revealed the observed level of genetic diversity, which could be exploited through various breeding schemes.

Based on the 1904 SNP markers used to investigate the population structure and the relationship patterns of the elite white inbred population, a discord was observed from the outputs of the Bayesian statistics approach in STRUCTURE software and the principal component analysis (PCA). While STRUCTURE concluded that, the optimum number of sub-populations was two and six, the alternative approach concluded that the optimum number of sub-populations was three. Additionally, PCA revealed closer genetic proximity between subpopulations 1 and 2, suggesting they could have been considered a single subpopulation by the Bayesian statistics approach in STRUCTURE software. The percentage of admixture resulting from K=2 (sub-populations) was far lower than compared to when k=6 suggesting that admixture levels increase with an increase in the level of stratification. The level of admixture in this study at both K values defined as optimum did not translate to genetic contamination but rather inbred lines that combined genetic components of more than one group (Zhang et al., 2016). In the present study, three sub-populations were considered optimum. Knowledge of population stratification is essential for explaining differences in the genetic architecture of any population though spatial and gene exchange isolation could affect the output (Huang et al., 2017).

The dissimilarity matrices and clustering methods employed for this study have implications on the results, hence the choice of an appropriate distance matrix and hierarchical clustering method is critical for determining the accuracy of the genetic variability among the 376 inbred lines. The IBS genetic distance matrix and the average linkage method were found to be the appropriate hierarchical clustering parameters for assigning the elite inbreds into the heterotic groups. Using correct genetic distance and proper linkage method can improve the precision of heterosis prediction as it ensures that parents with sufficient genetic divergence are identified for potential crossing. Additionally, by accurately assessing genetic relatedness, breeders can prioritize crosses with the highest potential for heterosis and thereby optimizing resource allocation. Furthermore, identification of appropriate genetic distance measures can lead to the development of hybrids with superior agronomic traits and higher yield potential (Badu-Apraku, 2021). The heterotic grouping of the elite inbreds was largely consistent with the pedigree and selection history of the inbreds. It is noteworthy that each of the heterotic groups had a number of inbreds from more than one source populations. This observation suggested that the source populations from where the 376 elite inbreds were developed could have shared alleles. The number of heterotic groups observed in this study was similar to that reported by (Adu et al., 2019) for a population of 94 white and yellow elite early inbreds using DArTseq SNP markers. In another study conducted by (Badu-Apraku et al., 2021) with a panel of 439 early and extra-early maize inbreds derived from diverse source populations, four heterotic groups were identified. The authors reported that the ideal heterotic groups for each maturity group and kernel colour should be two, labelled A and B or at most three labelled A, B, and C (the mixed group).

The paradigm shift from open-pollinated varieties to hybrid maize production in the West African sub-region since the 2000s has emphasized the development of inbred lines and their classification into heterotic groups (Badu-Apraku et al., 2021). Several methods have been employed at IITA to classify early maize inbred lines, including phenotypic data, specific combining ability, combining ability of multiple traits, and

more recently, DNA-based markers. However, heterotic groups are continuously refined as new inbred lines are developed from heterotic and broad-based populations (Akinwale et al., 2014, Agbaje et al., 2008). In this study, 661 hybrid combinations from opposing heterotic groups were identified as potentially resulting in outstanding hybrids (Figure 5). Hybrid maize production exploits heterosis, the enhanced vigour observed in progeny from two complementary inbred lines. Inbreds from different heterotic groups exhibit higher levels of heterosis compared to those from the same group. The latter can only exhibit high heterosis when crossed in a three-way hybrid combination with inbreds differing in specific characteristics (Badu-Apraku et al., 2021).

The analysis of molecular variance revealed higher levels of genetic variability within the heterotic groups than among the heterotic groups with a moderate estimate of genetic differentiation. A similar pattern of observation exists based on the pedigree or source populations. A deeper dive into the pairwise genetic differentiation among the observed heterotic groups revealed that heterotic groups I and III were characterized by high genetic differentiation and as such were suitable for the exploration of heterosis for early-maturity white hybrid maize production. These heterotic groups consisted largely of inbreds from the source populations TZEI 65 x ENT 11 and TZE-W Pop DT C5 STR C5. The justification for this observation could be the involvement of ENT 11 as a parental inbred of the heterotic population. ENT 11 is a CIMMYT inbred with a different genetic background compared to TZEI 65 or the broad-based populations developed by IITA. Inbreds derived from TZEI 65 x ENT 11 presumably acquired unique haplotype blocks through recombination during inbreeding, leading to greater genetic distance from those developed solely from IITA germplasm. Similar, though less pronounced, genetic differentiation was observed between Groups II and III. Conversely, Groups I and II exhibited the lowest FST values, suggesting they may not display significant heterosis in hybrid combinations. Inbreds within these groups originated from the IITA broad-based populations TZE-W Pop DT C5 STR C5 and DTE STR-W Syn Pop C4. Based on the understanding obtained from several reports, genetic differentiation values between up to 0.05 suggested low levels of differentiation, 0.06 and 0.15 as moderate, 0.16 and 0.25 as high, and above 0.25 as very high (Wright, 1930, Hartl & Clark, 2007). Understanding pairwise genetic differentiation is crucial for breeding cross-pollinated crops like maize, as it facilitates efficient exploitation of heterosis for hybrid development and enhanced selection gains (Kondwakwenda et al., 2020).

The observed gene flow pattern mirrored the inverse trend of genetic differentiation. The lowest gene flow, indicative of reduced allele sharing, occurred between Groups I and III, the most genetically differentiated groups, making them suitable for hybrid production. Conversely, the highest gene flow, suggesting substantial shared alleles, was observed between Groups I and II, potentially leading to lower heterosis in hybrid combinations. Gene flow exceeding unity signifies sufficient gene flow (Wright, 1930), explaining why the admixture-based model implemented in STRUCTURE grouped them into a single sub-population.

This study provides valuable data to guide early maturity white hybrid maize development at the IITA maize improvement program. Future efforts should focus on aligning these findings with existing heterotic groups and implementing strategies to refine these groups for optimal genetic gain and cost-effective hybrid production. Notably, the impracticality of managing six distinct heterotic groups for early maturity white maize, as suggested by the K= 6 outcome in STRUCTURE, necessitates a streamlining approach for efficient breeding practices.

CONCLUSIONS

The study effectively employed 1,904 SNP markers to characterize genetic diversity among 376 elite early maturing white maize inbred lines, revealing a complex genetic structure within the germplasm. The markers polymorphic information content showed that the markers were appropriate in explaining the

genetic differences among the inbreds and the heterozygosity information revealed the presence of substantial level of diversity in the inbred population. The combination of IBS distance matrix and the average linkage clustering methods was identified as the best technique for heterotic grouping of the 376 maize inbred population. Three distinct heterotic groups, each comprising inbreds from more than one source population were identified. This suggested the existence of genetic relatedness among the source populations. The observed genetic diversity within the inbred lines offers a promising foundation for developing superior maize hybrids. By capitalizing on the identified heterotic groups, breeders in the IITA-MIP can prioritize crosses with the greatest potential for heterosis, thereby accelerating the development of high-yielding, stress-tolerant hybrid maize genotypes. To maximize the impact of these findings, policymakers should invest in research to further refine heterotic group identification methods and support the development of marker-assisted selection tools. Breeders should adopt these advanced techniques to efficiently exploit genetic diversity and enhance maize productivity. Ultimately, these efforts will contribute to increased food security and improved livelihoods for small-scale farmers in Sub-Saharan Africa.

Supplementary Materials: The raw dataset for this study can be obtained from the IITA CKAN repository at Datasets - IITA. In addition, the following supporting information can be downloaded at Editorial Manager®: Table S1: Assignment of inbreds into clusters based on optimum sub-population at K= 2 using 1904 SNP markers; Table S2: Assignment of inbreds into clusters based on optimum sub-population at K= 6 using 1904 SNP markers; Table S3: Gene flow based on population structure and pedigree information of the 376-elite white inbreds; Table S4: Pedigree information of the 376 maize inbred lines genotyped using 1904 SNP markers; Figure S1: Pedigree-based PCA scatter plot of the 376 early maturing maize inbred lines along PC1 and PC2 using 1904 SNP markers; Figure S2: Optimal sub-population based on Elbow method (upper), Silhouette method (middle), and Gap statistics method (lower).

Author Contributions: Conceptualization, H.M.M., B.B.-A, J.D,; methodology, H.M.M., B.B.-A, I.I.A; supervisions, B.B.-A., J.D., A.A.,; formal analysis, I.I.A.; writing—original draft preparation, H.M.M.,; writing—review and editing, B.B.-A, J.D., I.I.A. and A.A.,. All authors have read and agreed to the published version of the manuscript.

Funding: This research was partly funded by African Union through Pan African University scholarship grant to H.M and the Bill and Melinda Gates Foundation for covering the publication through the Accelerated Genetic Gains and Stress Tolerant Maize for Africa Projects [OPP1134248].

Data Availability Statement: The datasets used in the present study have been deposited at the IITA CKAN repository.

Acknowledgments: The authors are grateful to the staff of the Maize Improvement Unit of the International Institute of Tropical Agriculture, Ibadan, Nigeria, the African Union Commission, and the African Development Bank through the Pan African University of Life and Earth Science Institute (PAULESI) for providing funds for the research. The authors are also grateful for the funding support of the Bill & Melinda Gates Foundation under the Accelerated Genetic Gains in Maize and Wheat (AGG) Projects.

Conflicts of Interest: The authors declare no conflict of interest

REFERENCES

Adu, G. B., Badu-Apraku, B., Akromah, R., Garcia-Oliveira, A. L., Awuku, F. J., & Gedil, M. (2019). Genetic diversity and population structure of early-maturing tropical maize inbred lines using SNP markers. PLoS ONE, 14(4). https://doi.org/10.1371/journal.pone.0214810

Agbaje, S. A., Badu-Apraku, B., & Fakorede, M. A. B. (2008). Heterotic patterns of early maturing maize inbred lines in Striga-free and Striga-infested evironments.

Akinwale, R. O., Badu-Apraku, B., Fakorede, M. A. B., & Vroh-Bi, I. (2014). Heterotic grouping of tropical early-maturing maize inbred lines based on combining ability in Striga-infested and Striga-free environments and the use of SSR markers for genotyping. Field Crops Research, 156, 48–62. https://doi.org/10.1016/J.FCR.2013.10.015

Amiteye, S. (2021). Basic concepts and methodologies of DNA marker systems in plant molecular breeding.

Badu-Apraku, B. M. G. A. L. G.-O. (2021). Genetic diversity and population structure of early and extra-early maturing maize germplasm adapted to sub-Saharan Africa. BMC Plant Biology, 21(96).

Baffour Badu-Apraku, & Fakorede, M. A. B. (2017). Advances in Genetic Enhancement of Early and Extra-Early Maize for Sub-Saharan Africa. Springer.

Chen, Z. L., Meng, J. M., Cao, Y., Yin, J. L., Fang, R. Q., Fan, S. B., Liu, C., Zeng, W. F., Ding, Y. H., Tan, D., Wu, L., Zhou, W. J., Chi, H., Sun, R. X., Dong, M. Q., & He, S. M. (2019). A high-speed search engine pLink 2 with systematic evaluation for proteome-scale identification of cross-linked peptides. Nature Communications, 10(1). https://doi.org/10.1038/s41467-019-11337-z

Erenstein, O., Jaleta, M., Sonder, K., Mottaleb, K., & Prasanna, B. M. (2022). Global maize production, consumption and trade: trends and R&D implications. In Food Security (Vol. 14, Issue 5, pp. 1295–1319). Springer Science and Business Media B.V. https://doi.org/10.1007/s12571-022-01288-7

Evanno, G., Regnaut, S., & Goudet, J. (2005). Detecting the number of clusters of individuals using the software STRUCTURE: A simulation study. Molecular Ecology, 14(8), 2611–2620. https://doi.org/10.1111/j.1365-294X.2005.02553.x

Gao, H., Williamson, S., & Bustamante, C. D. (2007). A Markov chain Monte Carlo approach for joint inference of population structure and inbreeding rates from multilocus genotype data. Genetics, 176(3), 1635–1651. https://doi.org/10.1534/genetics.107.072371

Gedil, M., Maazou, A. R. S., Zebire, D. A., Garcia-Oliveira, A. L., Unachukwu, N., Petroli, C., Hearne, S., Everett, L. A., Kim, S. K., & Menkir, A. (2023). Genetic structure analysis and identifying key founder inbred lines in diverse elite sub-tropical maize inbred lines. Scientific Reports, 13(1). https://doi.org/10.1038/s41598-023-38980-3

Hake, S., & Ross-Ibarra, J. (2015). Genetic, evolutionary and plant breeding insights from the domestication of maize. ELife, 2015(4). https://doi.org/10.7554/eLife.05861.001

Hartl, L., & Clark, G. (2007). Principles of Population Genetics D. L. Hartl, A. G. Clark. 2007. Principles of Population Genetics. 4th Edition Sinauer Associates. xv +. 628 18.5 × 24 cm, hardcover, US$93.95. ISBN: 978-0-878-93308-2. Ecoscience, 14(4), 544–545. https://doi.org/10.2980/1195-6860(2007)14 [544b: popg]2.0.co;2

Huang, C., Nie, X., Shen, C., You, C., Li, W., Zhao, W., Zhang, X., & Lin, Z. (2017). Population structure and genetic basis of the agronomic traits of upland cotton in China revealed by a

genome-wide association study using high-density SNPs. Plant Biotechnology Journal, 15(11), 1374–1386. https://doi.org/10.1111/pbi.12722

Iqbal, M., Saleem, M., & Rashid, O. (2001). Inter-racial heterosis in maize hybrids. Pakistan Journal of Scientific and Industrial Research, 44(4), 239–243.

Kilian, A., Wenzl, P., Huttner, E., Carling, J., Xia, L., Blois, H., Caig, V., Heller-Uszynska, K., Jaccoud, D., Hopper, C., Aschenbrenner-Kilian, M., Evers, M., Peng, K., Cayla, C., Hok, P., & Uszynski, G. (2012). Diversity arrays technology: A generic genome profiling technology on open platforms. Methods in Molecular Biology, 888, 67–89. https://doi.org/10.1007/978-1-61779-870-2_5

Kondwakwenda, A., Sibiya, J., Amelework, A. B., & Zengeni, R. (2020). Diversity analysis of provitamin A maize inbred lines using single nucleotide polymorphism markers. Acta Agriculturae Scandinavica Section B: Soil and Plant Science, 70(4), 265–271. https://doi.org/10.1080/09064710.2020.1718198

Lê, S., Josse, J., Rennes, A., & Husson, F. (2008). FactoMineR: An R Package for Multivariate Analysis. In JSS Journal of Statistical Software (Vol. 25). http://www.jstatsoft.org/

Li, D., Zhou, Z., Lu, X., Jiang, Y., Li, G., Li, J., Wang, H., Chen, S., Li, X., Würschum, T., Reif, J. C., Xu, S., Li, M., & Liu, W. (2021). Genetic Dissection of Hybrid Performance and Heterosis for Yield-Related Traits in Maize. Frontiers in Plant Science, 12. https://doi.org/10.3389/fpls.2021.774478

Melchinger, A. E., & Gumber, R. K. (1998). Concepts and Breeding of Heterosis in Crop Plants. In CSSA Special Publication (Issue 25).

Mohammadi, S. A., & Prasanna, B. M. (2003). Review & interpretation Analysis of Genetic Diversity in Crop Plants-Salient Statistical Tools and Considerations.

Moll, R. H., Lonnquist, J. H., Fortuno, E. Z., & Johnson, E. C. (1965). the relationship of heterosis and genetic divergence in maize. https://academic.oup.com/genetics/article/52/1/139/5988102

Okou, C., Spray, J., & Filiz, D. (2022). Staple Food Prices in Sub-Saharan Africa: An Empirical Assessment, WP/22/135, July 2022.

Padilla, G., Cartea, M. E., & Ordás, A. (2007). Comparison of Several Clustering Methods in Grouping Kale Landraces.

Paradis, E., & Schliep, K. (2019). Ape 5.0: An environment for modern phylogenetics and evolutionary analyses in R. Bioinformatics, 35(3), 526–528. https://doi.org/10.1093/bioinformatics/bty633

Peakall, R., & Smouse, P. E. (2012). GenALEx 6.5: Genetic analysis in Excel. Population genetic software for teaching and research-an update. Bioinformatics, 28(19), 2537–2539. https://doi.org/10.1093/bioinformatics/bts460

Ranum, P., Peña-Rosas, J. P., & Garcia-Casal, M. N. (2014). Global maize production, utilization, and consumption. Annals of the New York Academy of Sciences, 1312(1), 105–112. https://doi.org/10.1111/nyas.12396

R core team. (2017). A Language and Environment for Statistical Computing.

Semagn, K., Magorokosho, C., Vivek, B. S., Makumbi, D., Beyene, Y., Mugo, S., Prasanna, B. M., & Warburton, M. L. (2012). Molecular characterization of diverse CIMMYT maize inbred lines from eastern and southern Africa using single nucleotide polymorphic markers. BMC Genomics, 13(1). https://doi.org/10.1186/1471-2164-13-113

Tal, I., & Vardy, A. (2015). List Decoding of Polar Codes. IEEE Transactions on Information Theory,

61(5), 2213–2226. https://doi.org/10.1109/TIT.2015.2410251
Wright, S. (1930). Evolution in Mendelian populations.
Wu, X., Li, Y., Li, X., Li, C., Shi, Y., Song, Y., Zheng, Z., Li, Y., & Wang, T. (2015). Analysis of genetic differentiation and genomic variation to reveal potential regions of importance during maize improvement. BMC Plant Biology, 15(1). https://doi.org/10.1186/s12870-015-0646-7
Yu, G., Smith, D. K., Zhu, H., Guan, Y., & Lam, T. T. Y. (2017). ggtree: an r package for visualization and annotation of phylogenetic trees with their covariates and other associated data. Methods in Ecology and Evolution, 8(1), 28–36. https://doi.org/10.1111/2041-210X.12628
Zhang, X., Zhang, H., Li, L., Lan, H., Ren, Z., Liu, D., Wu, L., Liu, H., Jaqueth, J., Li, B., Pan, G., & Gao, S. (2016). Characterizing the population structure and genetic diversity of maize breeding germplasm in Southwest China using genome-wide SNP markers. BMC Genomics, 17(1). https://doi.org/10.1186/s12864-016-3041-3
Zhu, X. M., Shao, X. Y., Pei, Y. H., Guo, X. M., Li, J., Song, X. Y., & Zhao, M. A. (2018). Genetic diversity and genome-wide association study of major ear quantitative traits using high-density SNPs in maize. Frontiers in Plant Science, 9. https://doi.org/10.3389/fpls.2018.00966

Click here to access/download;Figure;Manuscript_figures.pptx

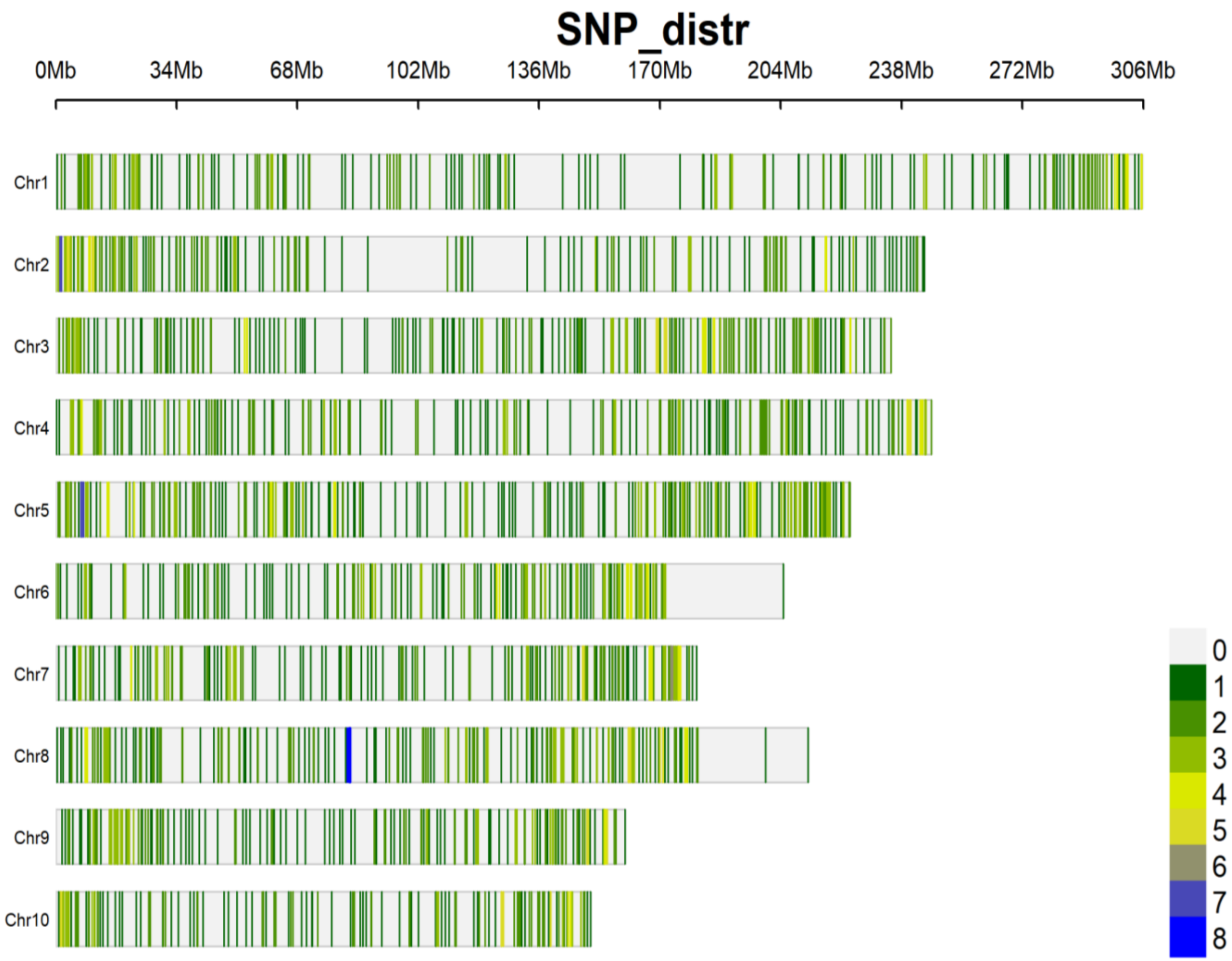

Chromosome	Number of SNP markers
CHR1	201
CHR2	186
CHR3	222
CHR4	201
CHR5	254
CHR6	171
CHR7	172
CHR8	194
CHR9	160
CHR10	143
Total	1904

Figure 1: Distribution of the 1904 SNP marker across the 10 chromosomes of the maize genome. The number in the key indicated the number of SNP marker corresponding to the color patterns depicted in the SNP distribution plot. Grey color means zero SNP marker, dark to light green indicated between one and three SNPs, light to dark yellow indicated between 4 and 6 SNPs, light and deep blue colorations indicated 7 and eight SNPs, respectively.

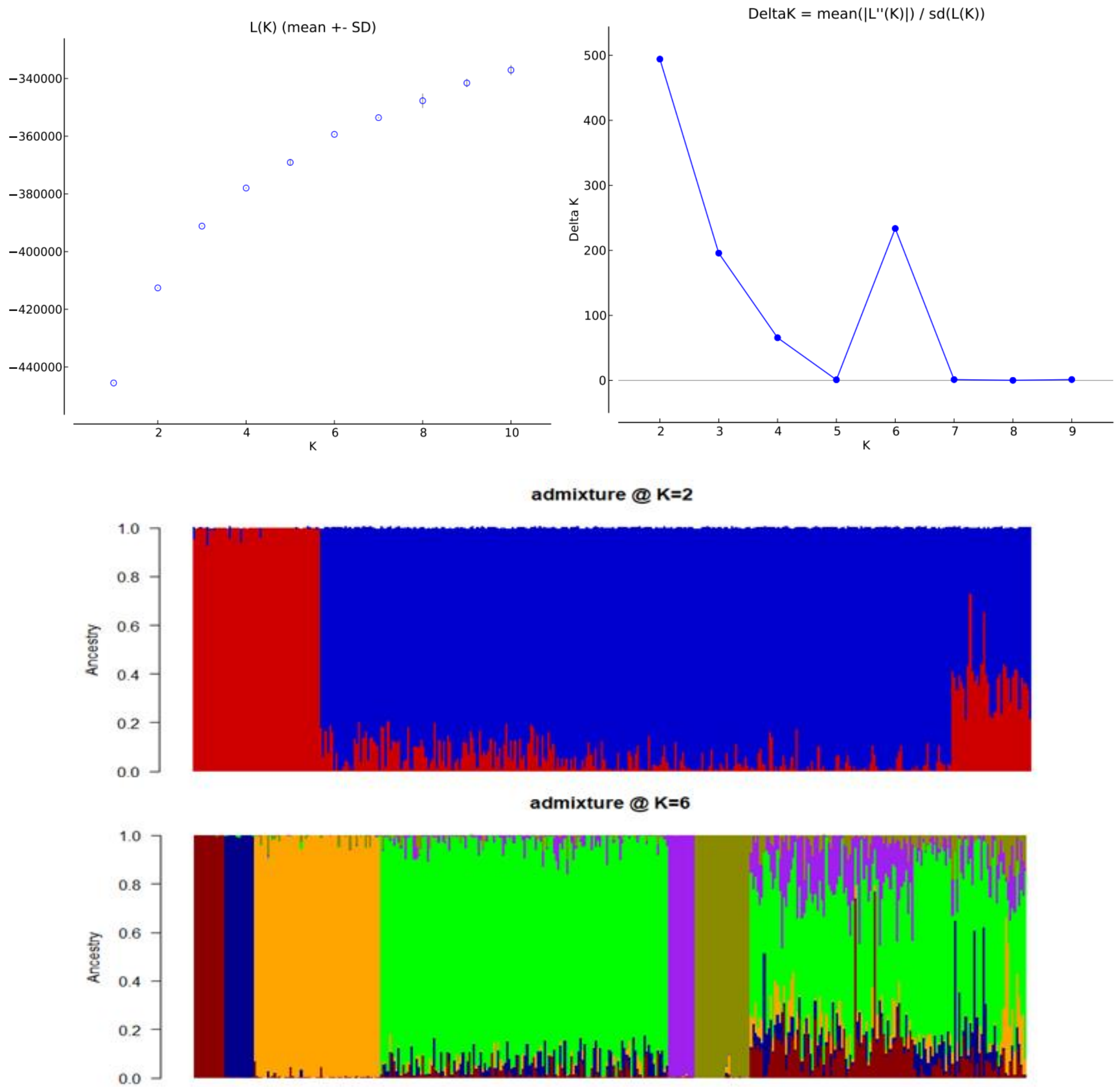

Figure 2: Population structure of 376 elite maize inbreds using 1904 DArTtag derived SNP markers revealed two inflections at two and six. (a) LnP(D), (b) K inflection showing optimum sub-populations, (c) plot view of optimum sub-population @ K=2 with red and blue representing sub-populations 1 and 2, respectively, and (d) plot view of optimum sub-population @ K=6 with red, blue, orange, green, purple, and yellow representing sub-populations 1,2,3,4,5 and 6, respectively.

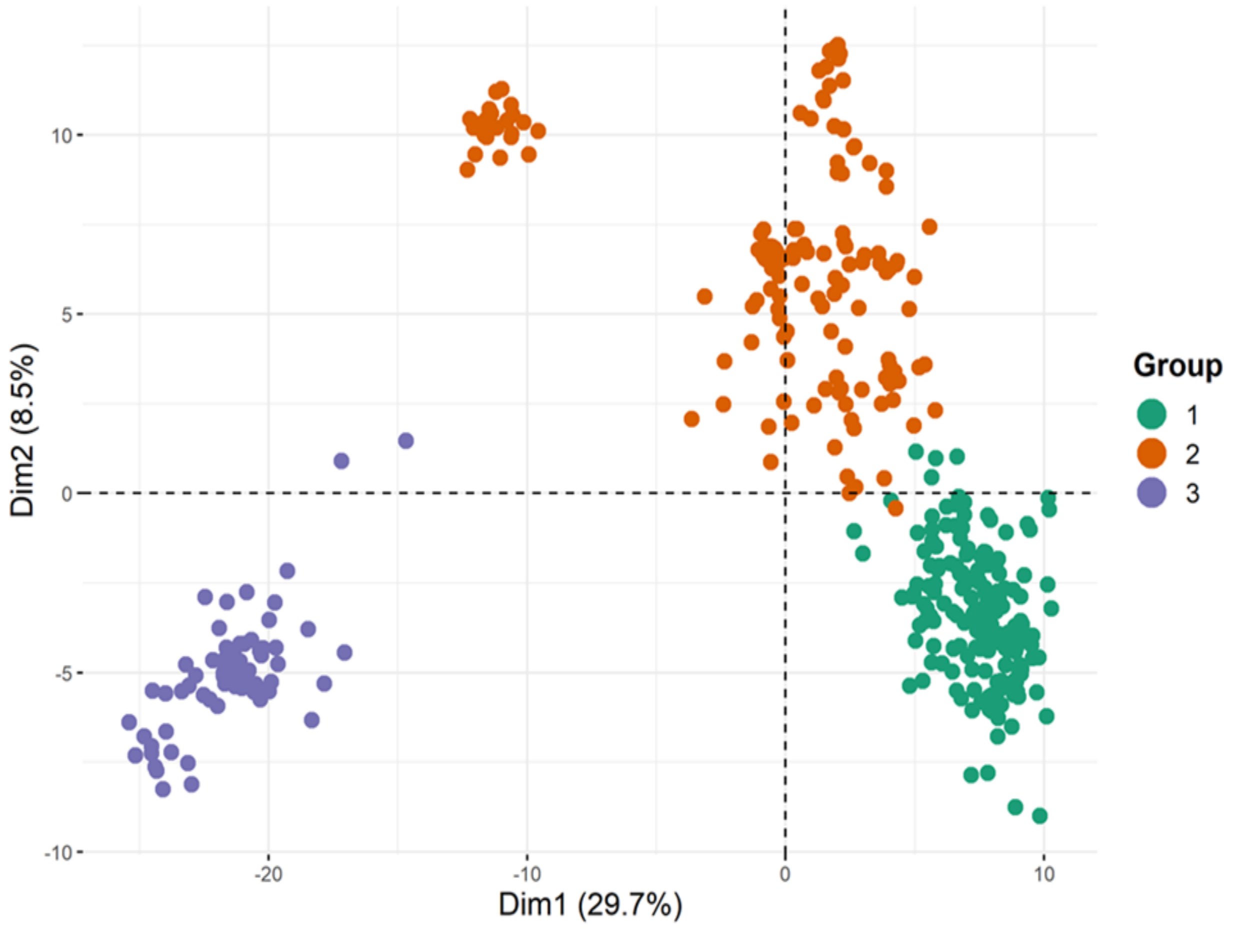

Figure 3: Principal component analysis showing the three groups characterizing the 376 maize inbreds assessed with 1904 SNP markers. Group one (green) and two (red) showed closer genetic distance with distinct sub-groups observed in sub-population two.

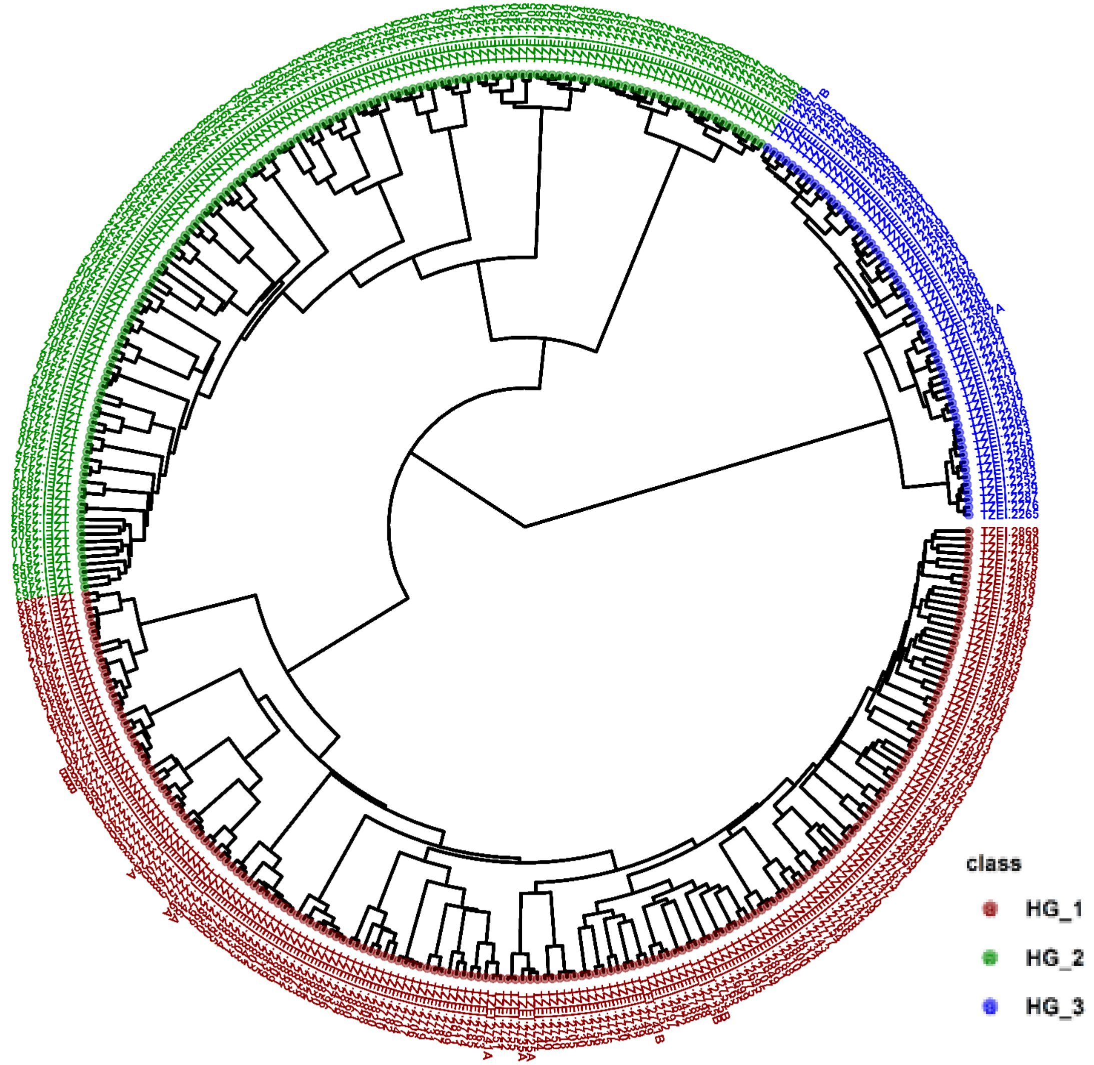

Figure 4: Heterotic grouping of 376 early-maturing white maize inbreds revealing three distinct groups. HG_1 means heterotic group one, HG_2 means heterotic group two, and HG_3 means heterotic group three.

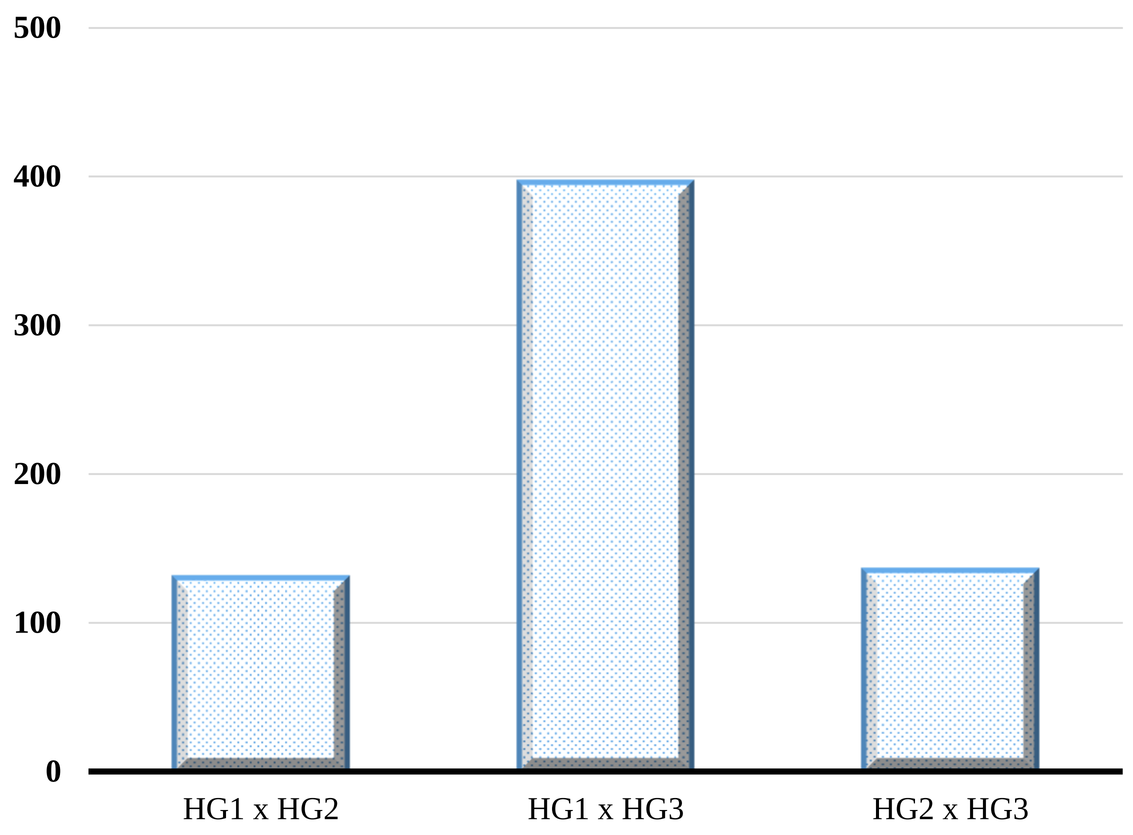

Figure 5: Potential hybrid combinations from opposing heterotic groups based on genetic dissimilarity of 35%. HG_1, heterotic group one; HG_2, heterotic group two; and HG_3, heterotic group three.

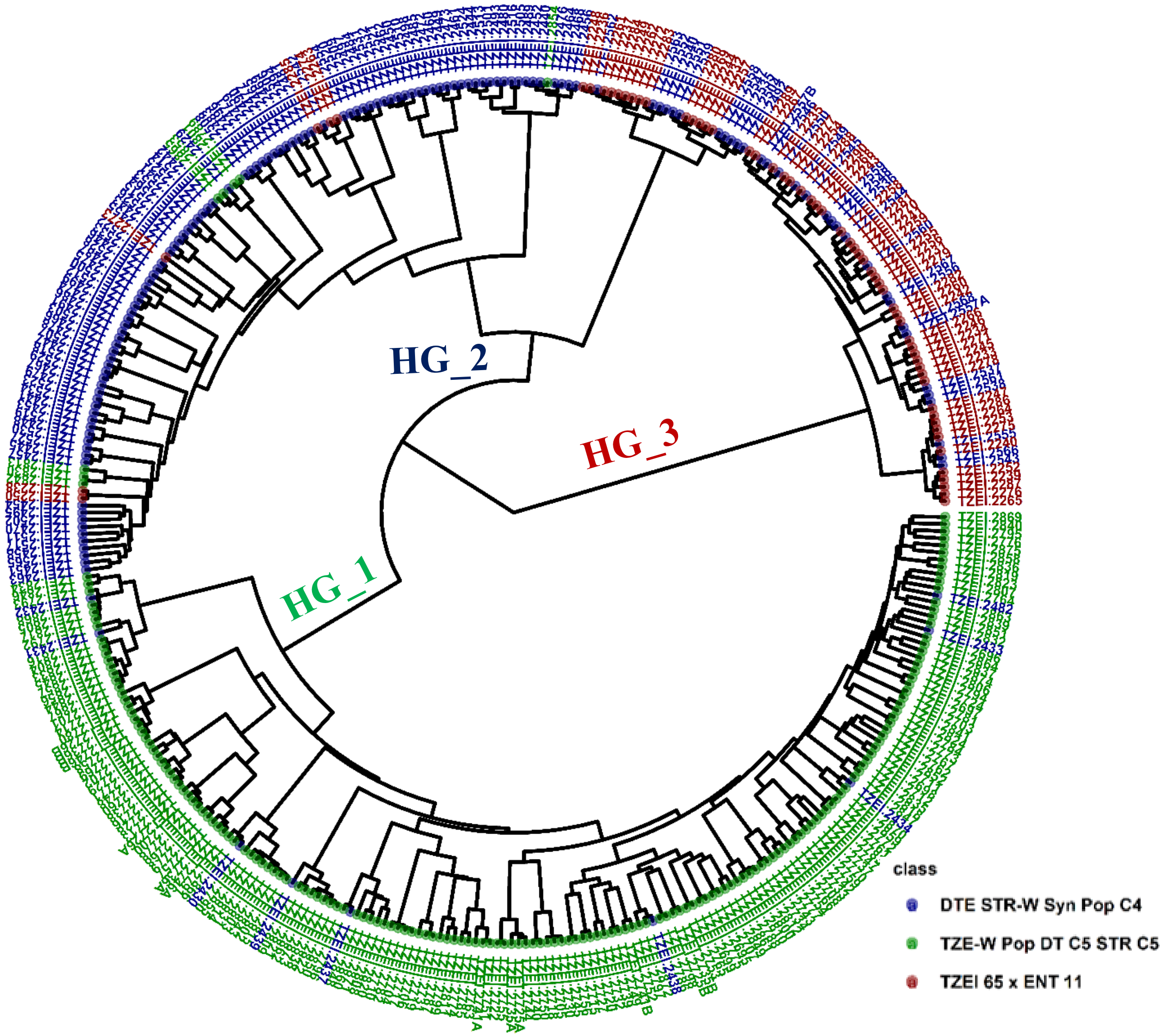

Figure 6: Phylogenetic relatedness among 376 early-maturing white maize inbreds based on the source populations from which the inbreds were developed. HG_1 means heterotic group one, HG_2 means heterotic group two, while HG_3 means heterotic group three.
